# The relationships of the Euparkeriidae and the rise of Archosauria

**DOI:** 10.1098/rsos.150674

**Published:** 2016-03-23

**Authors:** Roland B. Sookias

**Affiliations:** 1 School of Geography, Earth and Environmental Sciences, University of Birmingham, Edgbaston, Birmingham B15 2TT, UK; 2 GeoBio-Center, Ludwig-Maximilians-Universität München, Richard-Wagner-Straße 10, 80333 Munich, Germany

**Keywords:** Euparkeriidae, Archosauria, Triassic, Archosauriformes, phylogeny

## Abstract

For the first time, a phylogenetic analysis including all putative euparkeriid taxa is conducted, using a large data matrix analysed with maximum parsimony and Bayesian analysis. Using parsimony, the putative euparkeriid *Dorosuchus neoetus* from Russia is the sister taxon to Archosauria + Phytosauria. *Euparkeria capensis* is placed one node further from the crown, and forms a euparkeriid clade with the Chinese taxa *Halazhaisuchus qiaoensis* and ‘*Turfanosuchus shageduensis*’ and the Polish taxon *Osmolskina czatkowicensis*. Using Bayesian methods, *Osmolskina* and *Halazhaisuchus* are sister taxa within Euparkeriidae, in turn sister to ‘*Turfanosuchus shageduensis*’ and then *Euparkeria capensis*. *Dorosuchus* is placed in a polytomy with Euparkeriidae and Archosauria + Phytosauria. Although conclusions remain tentative owing to low node support and incompleteness, a broad phylogenetic position close to the base of Archosauria is confirmed for all putative euparkeriids, and the ancestor of Archosauria +Phytosauria is optimized as similar to euparkeriids in its morphology. Ecomorphological characters and traits are optimized onto the maximum parsimony strict consensus phylogeny presented using squared change parsimony. This optimization indicates that the ancestral archosaur was probably similar in many respects to euparkeriids, being relatively small, terrestrial, carnivorous and showing relatively cursorial limb morphology; this *Bauplan* may have underlain the exceptional radiaton and success of crown Archosauria.

## 1. Introduction

Archosauria, the diapsid clade represented today by birds and crocodilians and including the extinct dinosaurs, is highly speciose (with over 9000 species of extant bird and crocodilian [1]). Archosaurs filled most major terrestrial ecological niches for over 150 million years [2–7], from the Middle Triassic to the end of the Cretaceous. The ‘rise’ of the archosaurs to this position of ecological dominance took place coincident with the extinction or decline of many therapsid taxa at the end of the Permian and during the course of the Early–Middle Triassic, which had previously filled most large-bodied terrestrial niches [2,3,8–14]. The rise of the archosaurs is one of the landmark terrestrial faunal transitions and is an outstanding example of a large-scale adaptive radiation in the fossil record [2,15,16], with archosaurs diversifying into carnivorous, herbivorous, aquatic, terrestrial and volant forms of greatly varying sizes [2,14–17].

Understanding this adaptive radiation requires a thorough knowledge of archosaur phylogeny, of the morphological changes seen during this radiation and the sequence of these changes. The archosaur radiation must also be seen in its wider context as part of a radiation of archosauromorphs (those taxa more closely related to crown Archosauria than crown Lepidosauria), with aspects of the archosaur body plan beginning to develop within this stem lineage, and setting the stage for the unprecedented success of crown Archosauria. One such example is the development of cursorial locomotion [18], which may have allowed archosaurs to radiate into carnivorous niches following the extinction of therapsid carnivores.

**Figure 1.**
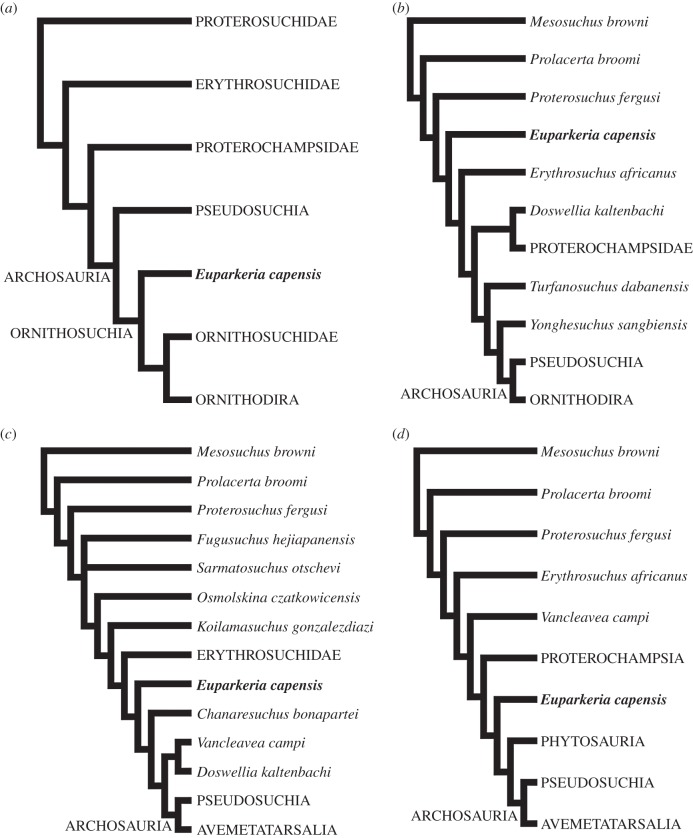
Previous phylogenetic positions found for *Euparkeria capensis*. (*a*) [20]; (*b*) [21]; (*c*) [22] and (*d*) [23].

The family Euparkeriidae has been historically composed of a number of small, gracile archosauriform taxa that have often been placed immediately outside or close to the base of Archosauria [19] (figure 1). They have often been discussed as potentially representing a morphology very close to that of the ancestral archosaur [24], although this idea has not been quantitatively investigated. However, irrespective of whether it represented a true phylogenetically intermediate step, the gracile, cursorial morphology of euparkeriids is roughly intermediate between more ‘sprawling’ early archosauromorph taxa and fully erect, and often bipedal, archosaurs [14,18,25]. Furthermore, given their phylogenetic position alone, these animals have the potential to shed light on the patterns seen during the archosaur radiation, and the factors that underlay archosaur success. The only taxon to be assigned with certainty to Euparkeriidae however is *Euparkeria capensis*, and the monophyly of the family has remained largely untested until recently. Here, for the first time, a phylogenetic analysis is conducted including all putative euparkeriids and a representative subset of stem and crown archosaurs, incorporating both new characters and those taken from previous analyses.

## 2. Previous phylogenetic work

### 2.1. Composition of Euparkeriidae

Following recent revisions, there are four valid species that may represent euparkeriids: *Euparkeria capensis* [26,27] (the type genus and species of the family Euparkeriidae), *Halazhaisuchus qiaoensis* (see [28]) and *Osmolskina czatkowicensis* (see [29,30]). Moreover, the holotype specimen of the nomen dubium ‘*Turfanosuchus shageduensis*’ has also been considered possibly referable to the clade [28]. Recent phylogenetic work has recovered *Halazhaisuchus qiaoensis*, ‘*Turfanosuchus shageduensis*’ and *Euparkeria capensis* within a euparkeriid clade, with the former two taxa being sister taxa to the exclusion of *Euparkeria capensis* [28], whereas *Dorosuchus neoetus* has been placed outside Euparkeriidae, one node closer to the crown, as the sister taxon to Archosauria + Phytosauria [31]. The only previous analysis to include *Osmolskina czatkowicensis* recovered it further down the archosaur stem than *Euparkeria capensis* [22], although this analysis was carried out before full description of the former taxon. No previous phylogenetic analysis has simultaneously included all of these species.

### 2.2. Position of Euparkeriidae

Most recent work has placed *Euparkeria capensis* and other euparkeriids close to the base of, but outside, Archosauria (see for example the summaries in [32,33], the placement hypothesized by Borsuk-Białynicka & Evans [30], and the phylogenetic analyses of Benton & Clark [34], Sereno & Arcucci [35], Sereno [36], Juul [37], Bennett [38], Benton [39], Parker & Barton [40], Nesbitt *et al*. [41], Dilkes & Sues [21], Nesbitt [23], Brusatte *et al*. [2], Ezcurra *et al*. [22], Desojo *et al*. [42], Dilkes & Arcucci [43], Schoch & Sues [44], Sookias *et al*. [28,31], Parrish [45]; figure 1*b*–*d*). This contrasts with the placement of *Euparkeria capensis* within the crown, as the sister taxon to Ornithosuchidae + Ornithodira (within ‘Ornithosuchia’), found in an early analysis by Gauthier [20] (figure 1*a*).

Less consensus has been reached regarding the relationships between euparkeriids and other stem archosaurs. Several analyses have placed *Euparkeria capensis* as the sister taxon to Archosauria [34,38,41,45] or Phytosauria + Archosauria [23,28,31]. However, many previous analyses have also placed proterochampsids, or proterochampsids + doswelliids, closer to the crown than is *Euparkeria capensis* [2,22,35–37,42] (Benton [39] presents this relationship, but the opposite topology is no less parsimonious; figure 1*b*,*c*).

All previous phylogenetic work has placed doswelliids more crownward than euparkeriids ([21,22,40,42,44]; figure 1*b*,*c*), either as the sister taxon to ([21,44] when excluding *Tarjadia ruthae*; figure 1*b*), in a polytomy with ([40,44] when including *Tarjadia ruthae*), or more derived than [22,42] (figure 1*d*) proterochampsids.

The analysis of Dilkes & Sues [21] (figure 1*b*) was unique in placing *Erythrosuchus africanus* closer to the crown than *Euparkeria capensis*, whereas Ezcurra *et al*. [22] placed erythrosuchids closer to the crown than *Osmolskina czatkowicensis*. All other phylogenetic analyses to date [22,23,28,31,40–42,44,46] have placed *Euparkeria capensis* and other euparkeriids or taxa until recently considered euparkeriids (namely *Dorosuchus neoetus*, *Halazhaisuchus qiaoensis*, ‘*Turfanosuchus shageduensis*’) *crownwards* of erythrosuchids, including those expanding the dataset of Dilkes & Sues [21] (e.g. [22,42]), and several questions have been raised regarding the validity of some of the scorings in the matrix of Dilkes & Sues [21], including by the first author [43].

## 3. Material and methods

A large, phylogenetic dataset for early archosauromorphs was created, based on the modified matrix of Nesbitt [23] used by Sookias *et al*. [28] (matrix in electronic supplementary material, and archived on Dryad; which includes the euparkeriid *Halazhaisuchus qiaoensis* and the holotype specimen of the nomen dubium ‘*Turfanosuchus shageduensis*’). Several crown taxa were pruned because they were members of clades adequately or better represented by other taxa, and because it was unnecessary to include a large taxon sample of groups within which putative euparkeriids would undoubtedly not be placed (e.g. Dinosauria). Interrelationships of crown taxa were one of the key focuses of the analysis of Nesbitt [23], and thus including even highly incomplete crown taxa and a large sample of each crown group was of relevance, but this is not the case for the current analysis. Instead, it was important to adequately represent all crown groups and character states in these groups. Given the large number of additional characters added, maintaining all crown taxa in the original matrix would also have substantially delayed completion of the work, and thus unnecessarily held back our knowledge of the placement of putative euparkeriid taxa. The following crown taxa were pruned: all theropod dinosaurs except *Herrerasaurus ischigualaestensis*, *Eoraptor lunensis* and *Coelophysis bauri*; all ornithischian dinosaurs except *Lesothosaurus diagnosticus* and *Heterodontosaurus tucki*; the non-dinosaurian dinosauromorphs *Dromomeron gregorii*, *Dromomeron romerii*, *Eucoelophysis baldwini* and *Sacisaurus agudoensis*; the non-crocodylomorph pseudosuchians CM 73372, *Polonosuchus silesiacus*, *Postosuchus alisonae*, *Sillosuchus longicervix*, *Shuvosaurus inexpectatus*, *Lotosaurus adentus* and *Poposaurus gracilis*; and crocodylomorphs except *Sphenosuchus acutus* and *Dromicosuchus grallator*.

To improve taxonomic sampling in the area of the phylogeny of particular relevance to Euparkeriidae, a number of taxa generally thought to be placed on the archosaur stem were added: the erythrosuchids *Garjainia prima* and *Shansisuchus shansisuchus*; the doswelliids *Doswellia kaltenbachi*, *Archeopelta arborensis* and *Jaxtasuchus salomoni*; the proterochampsid *Proterochampsa barrionuevoi*; and the early archosauromorphs *Protorosaurus speneri* and *Trilophosaurus buettneri*. *Youngina capensis* was also added as an outgroup, and the enigmatic archosauriform *Koilamasuchus gonzalezdiazi* was added, as what is known of its anatomy is broadly similar to that of *Euparkeria capensis* [22]. In order to test the monophyly of Euparkeriidae, *Dorosuchus neoetus* and *Osmolskina czatkowicensis* were added. The Russian archosauriform *Dongusuchus efremovi* (see [47,48]) was included in the matrix because it has been suggested to have euparkeriid affinities, but was excluded from the main analysis presented here; it is highly incomplete, and greatly reduced the resolution of the phylogeny (see below).

Although the referral of specimens to the hypodigm of *Dorosuchus neoetus* remains tentative, this analysis included all referred material as a single terminal taxon following Sookias *et al.* [31]. Similarly, *Osmolskina czatkowicensis* was scored as a single terminal taxon based on all referred material; the holotype consists only of an anterior maxilla [29], meaning analysing it alone is unlikely to be informative, and there is no reason to doubt the referral of any of the referred elements more than that of any others, making analysing subcombinations of material similarly uninformative. It must be noted strongly, however, that referral of all material other than the maxilla to *Osmolskina czatkowicensis* remains extremely uncertain and thus do conclusions based on the referred material of this taxon; no autamorphic euparkeriid characters unite the referred material, and at least one other archosauriform is found in the same assemblage.

All characters of Butler *et al*. [46] which ceased to be phylogenetically informative (i.e. all taxa, or all taxa bar one, were scored identically) following taxon pruning were removed, along with characters 100, 156 and 157 of Nesbitt [23] because it was considered that the variation referred to in their states could not be reliably observed (see [31]). An additional 50 characters from other datasets were added, including characters from Dilkes [49], Ezcurra [22], Desojo *et al*. [42], Dilkes & Arcucci [43] and Ezcurra *et al*. [50] (see electronic supplementary material). Five new characters were added based on personal observations, yielding a total of 405 characters. These new characters, their distribution and delineation are outlined in the electronic supplementary material, also archived on Dryad.

The main analysis was however conducted excluding one of these characters (character 93 in the dataset). This character was based on the observations of Borsuk-Białynicka & Evans [30] for *Osmolskina czatkowicensis* (‘Pterygoid, ridge along posteromedial corner, separating palatal flange from neck’). This character is potentially of particular relevance to euparkeriid phylogeny, as it was identified as a synapomorphy uniting *Euparkeria capensis, Osmolskina czatkowicensis* and other archosauromorph taxa; however, the state delimitation and scoring for this character were found to be very challenging, so it was not included in the main phylogenetic analysis presented.

The dataset was analysed in TNT v. 1.1 [51,52] using equally weighted parsimony. An initial ‘new technology search’ (with sectorial search, ratchet and tree-fusing options with default parameters) was carried out. Those characters that were treated as ordered in the source matrices were also treated as ordered here (all ordered characters were from Nesbitt [23]) along with one of the new characters (shape of iliac preacetabular process: character 268). The trees were stored in the random access memory (RAM) after minimum tree length had been obtained for 1000 replicates, and a heuristic tree bisection–reconnection (TBR) branch-swapping search was conducted. Standard bootstrap values and decay indices (Bremer support; using the Bremer script) were calculated for each node. The effect on decay indices of excluding incompletely scored taxa was investigated, with *Koilamasuchus gonzalezdiazi,* ‘*Turfanosuchus shageduensis*’, *Halazhaisuchus qiaoensis* and *Dorosuchus neoetus* successively excluded in decay index calculation, followed by the exclusion of all taxa less than 50% completely scored. Unambiguous synapomorphies were mapped for all nodes.

Additionally, a Bayesian analysis was carried out using MrBayes v. 3.2.2 [53]. An Mk + gamma model was used as this has been shown to be less affected by the absence of autapomorphies [54], which were not included in the dataset. The analysis used four chains sampled every 100 generations, with the analysis set to stop once it reached a standard deviation of split frequencies of 0.01. The first quarter of the generations were discarded as burn in.

A reduced consensus analysis failed to improve resolution of the tree, and is thus not presented. Although majority rule consensus trees did yield fewer polytomies than a strict consensus analysis, given that the strict consensus was relatively well resolved, that a strict consensus is the most conservative approach, and that the cut-off point in a majority rule consensus is somewhat arbitrary, and given the fact that a majority rule excludes a number of relationships which are equally parsimonious to those seen in the tree, these were not presented. The number of phylogenetic characters in the dataset characterizing fully quantitative variation was deemed insufficient to warrant quantitative delineation of character states using methods such as gap weighting [55].

To examine the likely morphology of the common ancestor of Archosauria and Phytosauria, character states were optimized using squared change parsimony onto the parsimony phylogeny presented here, including two additional ecological descriptors—femoral length (as a proxy for body size), and terrestriality (whether generally considered to have been terrestrial, aquatic or volant).

### 3.1. Institutional abbreviations

AMNH, American Museum of Natural History, New York, USA.

BP, Evolutionary Studies Institute (formerly Bernard Price Institute), University of the Witwatersrand, Johannesburg, South Africa.

GPIT, see IFGT.

IFGT, Institute for Geosciences, Eberhard-Karls-Universität Tübingen, Tübingen, Germany (formerly Geologisch-Paläontologisches Institut Tübingen, GPIT).

IGM, Institute of Geology, Ulaanbaatar, Mongolia.

ISI, Indian Statistical Institute, Kolkata, India.

IVPP, Institute of Vertebrate Paleontology and Paleoanthropology, Beijing, China.

MACN-Pv, Museo Argentino de Ciencias Naturales ‘Bernardino Rivadavia’, Paleontología de Vertebrados, Buenos Aires, Argentina.

MCZ, Museum of Comparative Zoology, Harvard University, Cambridge, USA.

NHMUK PV, Natural History Museum, London, UK.

PIN, Paleontological Institute of the Russian Academy of Sciences, Moscow, Russia.

PULR, Universidad Nacional de La Rioja, Paleontología, La Rioja, Argentina.

PVL, Instituto Miguel Lillo, Universidad Nacional de Tucumán, Tucumán, Argentina.

PVSJ, División de Paleontología, Museo de Ciencias Naturales de la Universidad Nacional de San Juan, Argentina.

SAM, Iziko South African Museum, Cape Town, South Africa.

SMNS, Staatliches Museum für Naturkunde, Stuttgart, Germany.

UCMP, University of California Museum of Paleontology, Berkeley, USA.

UMZC, University Museum of Zoology, University of Cambridge, Cambridge, UK.

ZPAL, Institute of Paleobiology, Polish Academy of Sciences, Warsaw, Poland.

## 4. Results

The parsimony analysis yielded 16 most parsimonious trees of 1330 steps, with a consistency index of 0.358 and a retention index of 0.688. *Dorosuchus neoetus* was the sister taxon to Archosauria + Phytosauria, and all other euparkeriids formed a clade which was the sister taxon to *Dorosuchus neoetus* + (Archosauria + Phytosauria). Within the euparkeriid clade, *Halazhaisuchus qiaoensis*, ‘*Turfanosuchus shageduensis*’ and *Osmolskina czatkowicensis* formed a subclade to the exclusion of *Euparkeria capensis*. One additional step was required to place *Dorosuchus neoetus* as the sister taxon to the other euparkeriids. Character states optimizing as autapomorphies for each taxon and synapomorphies supporting Euparkeriidae, the subclade within Euparkeriidae, and the position of *Dorosuchus neoetus* are listed in table 1, and a full synapomorphy list for all clades is given in the electronic supplementary material.

**Table 1.** Synapomorphies supporting key clades and autapormorphies of euparkeriid taxa. Numbers refer to character numbers and state changes.

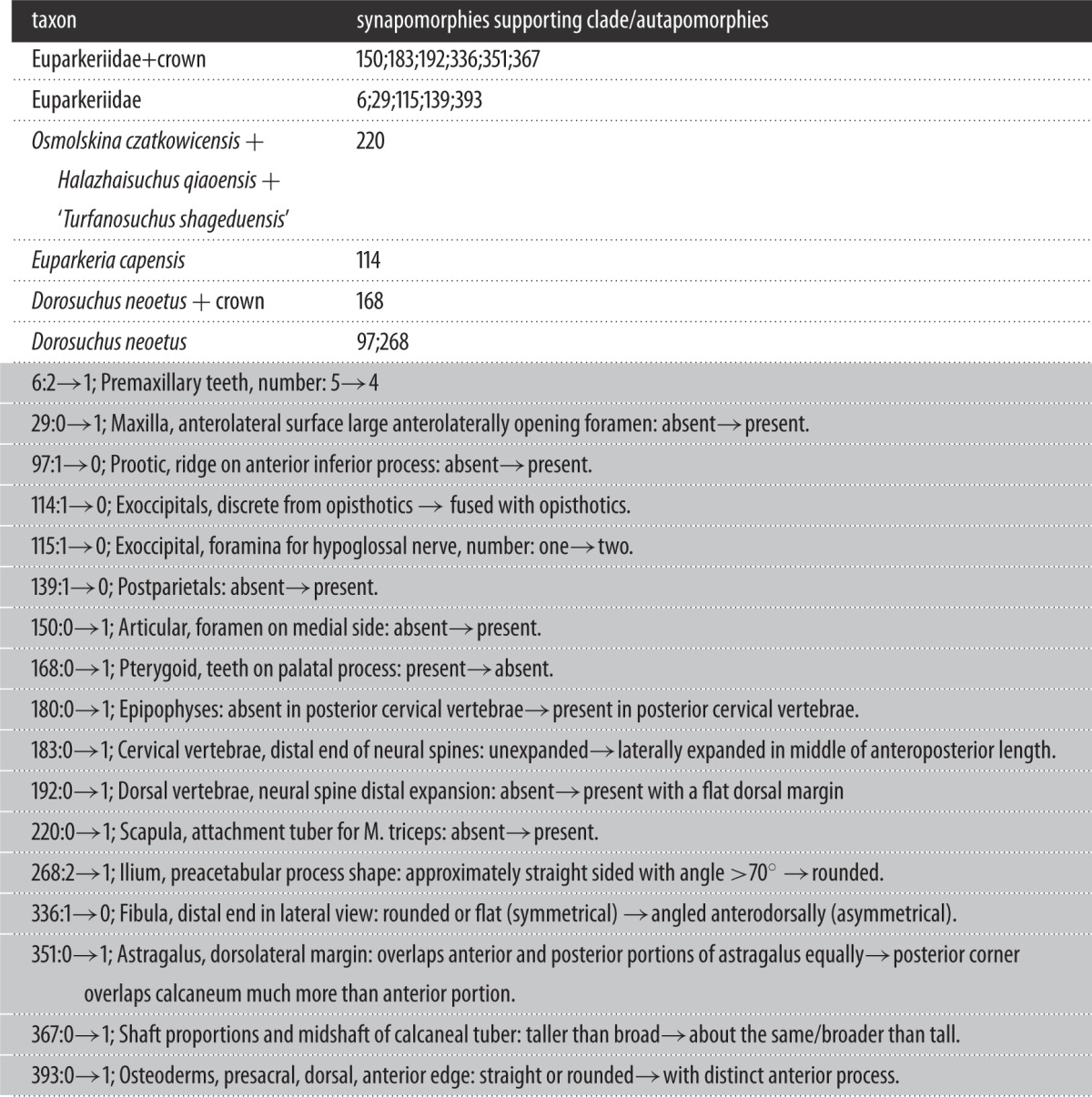

**Figure 2.**
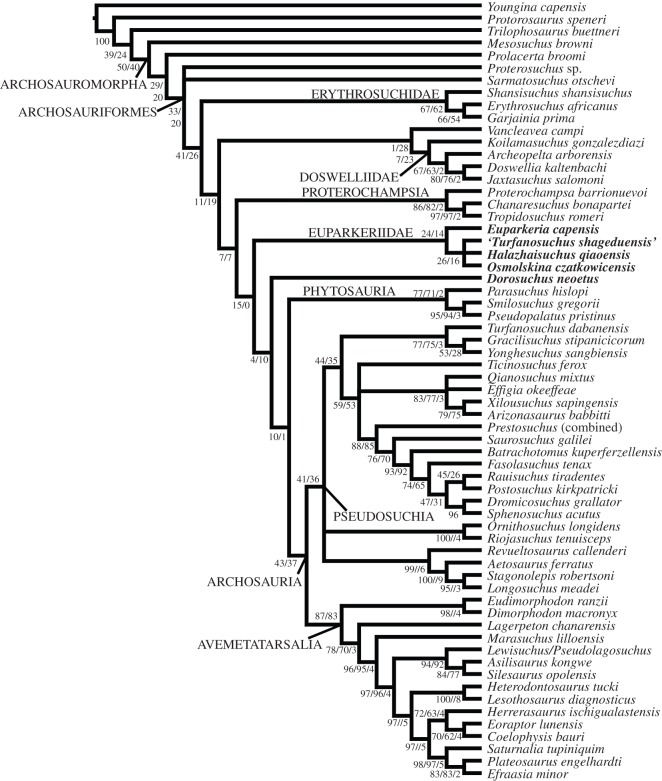
Strict consensus tree of 16 most parsimonious trees, showing the position of all putative euparkeriid taxa (bold). Numbers at nodes are standard bootstrap values (before first slash), GC bootstrap values (after first slash; only given if different from standard bootstrap value) and decay (=Bremer) indices (after second slash; only given if greatet than 1).

Bootstrap and decay indices supporting the position of Euparkeriidae and that of *Dorosuchus neoetus* were very low (bootstrap less than 20; decay index of 1), as were those supporting Euparkeriidae and the subclade within Euparkeriidae (bootstrap less than 30; decay index of 1) (figure 2). Decay indices did not change for these nodes with successive exclusion of *Koilamasuchus gonzalezdiazi* and incompletely scored putative euparkeriids (*Halazhaisuchus qiaoensis*, ‘*Turfanosuchus shageduensis*’, *Dorosuchus neoetus*), nor when all taxa in the dataset with more than 50% missing data were excluded. When the additional character (character number 93 in the dataset) was included, resolution was greatly reduced, though the recovered topology was similar to that recovered when the character was excluded (see electronic supplementary material, figure S1). Similarly, with *Dongusuchus efremovi* included, resolution was greatly reduced and *Dongusuchus efremovi* was placed as part of a large polytomy including *Sarmatosuchus otschevi* and all stem taxa crownward of *Proterosuchus* in the main phylogeny presented here, as well as phytosaurs (see electronic supplementary material, figure S2).

**Figure 3.**
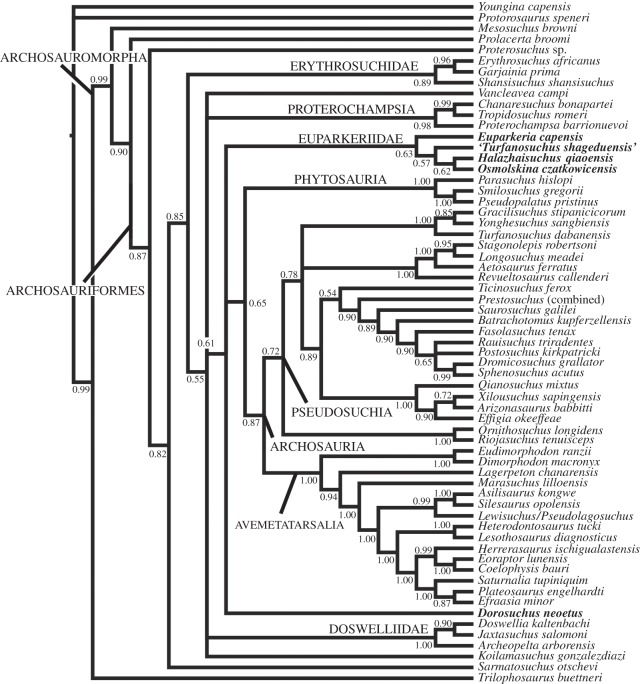
Bayesian consensus tree, showing the position of all putative euparkeriid taxa (bold). Numbers at nodes are posterior probabilities.

The Bayesian analysis yielded a topology very similar to the strict consensus tree of the parsimony analysis, with the positions of and relationships among euparkeriids differing only in that *Halazhaisuchus qiaoensis* and *Osmolskina czatkowicensis* were found to be sister taxa to the exclusion of ‘*Turfanosuchus shageduensis*’, and that *Dorosuchus neoetus* was placed in a polytomy with Euparkeriidae and Phytosauria + Archosauria (figure 3) rather than as the sister taxon to the latter clade. The Bayesian analysis showed two more polytomies than the parsimony analysis, with the polytomies being larger, including several clades (table 2).

**Table 2.** Table listing polytomies found with parsimony versus Bayesian analyses.

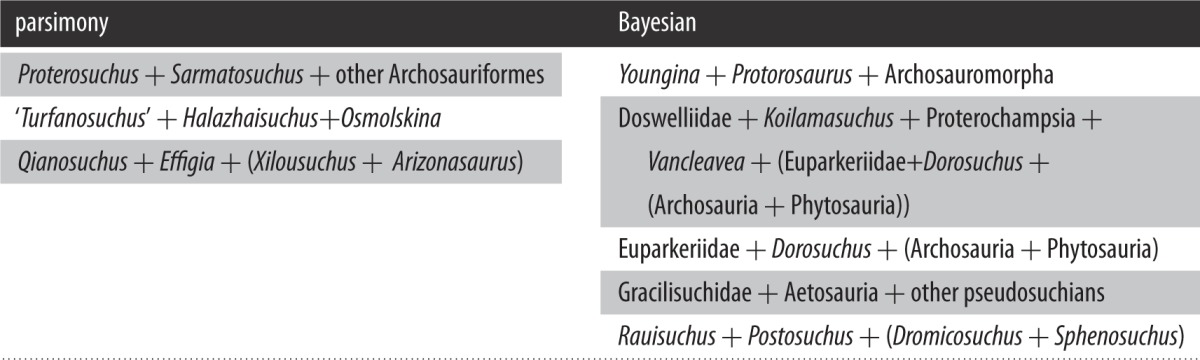

A total of 575 000 generations were required to reach a standard deviation of split frequencies of 0.01. The posterior probability of a monophyletic Euparkeriidae was low (0.61), as were the posterior probabilities of the relationships within Euparkeriidae (less than or equal to 0.60), of a monophyletic Phytosauria + Archosauria to the exclusion of *Dorosuchus neoetus* (0.67), and of the clade (Euparkeriidae, *Dorosuchus neoetus* (Phytosauria + Archosauria)) to the exclusion of proterochampsids and other stem taxa (0.62).

Based on the results of the optimization, the ancestor of crown Archosauria and Phytosauria was a relatively small (215 mm estimated femoral length), cursorial and gracile (e.g. fourth trochanter present—character 310, state 1—indicating relatively upright locomotion; femoral distal condyles not projecting markedly beyond shaft—character 313, state 1—contrasting with the robust femora of erythrosuchids and proterosuchids), terrestrial (optimized as terrestrial, and lacking pit and ridge dermal ornamentation—character 33, state 0—which is typical of aquatic taxa) and carnivorous (possessing serrated, bladelike, mediolaterally compressed marginal teeth—character 160, state 1, character 164, state 0 and character 165, state 1, respectively) animal (see electronic supplementary material, figures S3–10 showing optimizations).

### 4.1. Systematic palaeontology

#### 4.1.1

Diapsida Osborn, 1903 [56].

#### 4.1.2

Archosauromorpha Huene, 1946 [57] *sensu* Gauthier *et al.*, 1988 [58].

#### 4.1.3

Archosauriformes Gauthier *et al.,* 1988 [58] *sensu* Nesbitt, 2011 [23].

#### 4.1.4

Euparkeriidae Huene, 1920 [59] *sensu* Sookias & Butler, 2013 [19].

##### 4.1.4.1

*Phylogenetic definition.* Stem-based definition—the most inclusive clade containing *Euparkeria capensis* Broom, 1913 [26] but not *Crocodylus niloticus* Laurenti, 1768 [60] or *Passer domesticus* Linnaeus, 1758 [61] (from Sookias & Butler [19]).

##### 4.1.4.2

*Included taxa and specimens*. *Euparkeria capensis* Broom, 1913 [26] (=*Browniella africana* [27]), *Halazhaisuchus qiaoensis* Wu, 1982 [62], *Osmolskina czatkowicensis* Borsuk-Białynicka & Evans, 2003 [29], holotype of ‘*Turfanosuchus shageduensis*’ Wu, 1982 [62].

##### 4.1.4.3

*Excluded taxa historically referred to Euparkeriidae*. *Dorosuchus neoetus* Sennikov, 1989 [63], ‘*Wangisuchus tzeyii*’ Young, 1964 [64], *Turfanosuchus dabanensis* Young, 1973 [65], *Xilousuchus sapingensis* Wu, 1981 [66], *Platyognathus hsui* Sennikov, 1989 [67], *Dongusia colorata* Huene, 1940 [68].

##### 4.1.4.4

*Distribution*. Late Early Triassic (late Olenekian) of Poland, early Middle Triassic (early Anisian) of South Africa, Early or Middle Triassic (late Olenekian or early Anisian) of China.

##### 4.1.4.5

*Diagnostic synapomorphies.* Non-crown archosauriforms with the following local synapomorphies: four premaxillary teeth; two foramina for hypoglossal nerve on exoccipital; vertically orientated parabasisphenoid; discrete interparietals (=postparietals); two rows of keeled dorsal paramedian osteoderms with distinct anterior point/process (all differentiating euparkeriids from many stem taxa and some crown taxa).

##### 4.1.4.6

*Further differential diagnosis*. Absence of ossified astragalocalcaneal canal (differentiating euparkeriids from non-archosauriform archosauromorphs and proterosuchids); large anterolaterally opening foramen on anterolateral surface of maxilla at base of anterodorsal process; calcaneal tuber deflected approximately between 20° and 50° posteriorly; incompletely ossified medial wall of vestibule (=otic capsule); field of pointed, recurved teeth on pterygoid (all differentiating euparkeriids primarily from crown taxa).

##### 4.1.4.7

**Figure 4.**
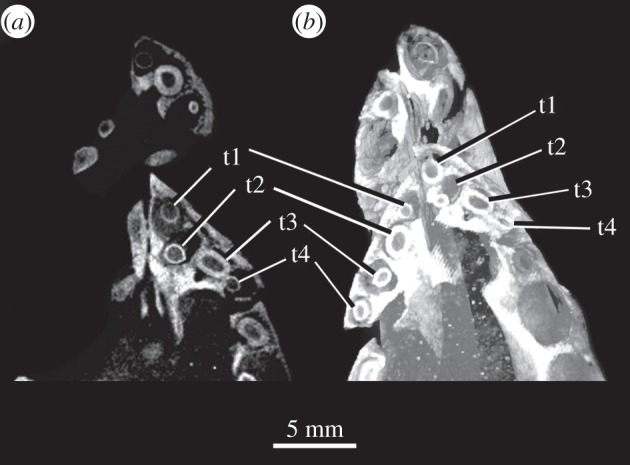
*Euparkeria capensis* SAM-PK-6047A premaxillary teeth as (*a*) CT slice and (*b*) volume reconstruction from CT data. t, tooth.

*Comments*. *Osmolskina czatkowicensis* has four premaxillary teeth [29,30] and, contra Nesbitt [23], *Euparkeria capensis* has four, not three, premaxillary teeth (figure 4). Five premaxillary teeth are seen in many stem archosaurs including erythrosuchids [69], *Vancleavea campi* [41], proterochampsids [23,70] and in several pseudosuchian taxa (e.g. *Turfanosuchus dabanensis* [71], *Xilousuchus sapingensis* [66]). Proterosuchids (e.g. *Proterosuchus goweri*—NMQR 880), phytosaurs (e.g. *Smilosuchus gregorii*—UCMP 27200), and other taxa with elongated premaxillae (e.g. *Qianosuchus mixtus*—IVPP V 13899 [23]) have more than five premaxillary teeth, whereas some taxa lack premaxillary teeth entirely (*Effigia okeeffeae* [72]). A large number of crown archosaurs show four premaxillary teeth [23] (e.g. *Batrachotomus kupferzellensis* [73], *Herrerasaurus ischigualastensis* [74]).

Presence of two foramina for the hypoglossal nerve differentiates euparkeriids from erythrosuchids [75], proterochampsids [43], doswelliids [42] and many crown taxa (e.g. *Batrachotomus kupferzellensis* [76], *Arizonasaurus babbitti* [77]), all of which possess one. A vertically orientated parabasisphenoid is seen in most crown archosaurs [23,78], erythrosuchids [72,75] and proterochampsids [23,43], whereas that of *Mesosuchus browni*, *Prolacerta broomi*, proterosuchids [23,78], doswelliids [21,42], *Trilophosaurus buettneri* [79] and *Youngina capensis* [80] is more horizontally orientated.

Presence of a separately ossified interparietal in *Euparkeria capensis* (SAM-PK-5867) and *Osmolskina czatkowicensis* (inferred based on the form of the parietals [30]) differentiates euparkeriids from crown taxa (e.g. *Batrachotomus kupferzellensis* [73], *Herrerasaurus ischigualastensis* [74]), proterochampsids (e.g. *Chanaresuchus bonapartei* [70]) and doswelliids (*Doswellia kaltenbachi* [21]), but is the same condition seen in many stem taxa including erythrosuchids (e.g. *Erythrosuchus africanus* [69]) and proterosuchids (e.g. *Proterosuchus fergusi* [81]) [23].

**Figure 5.**
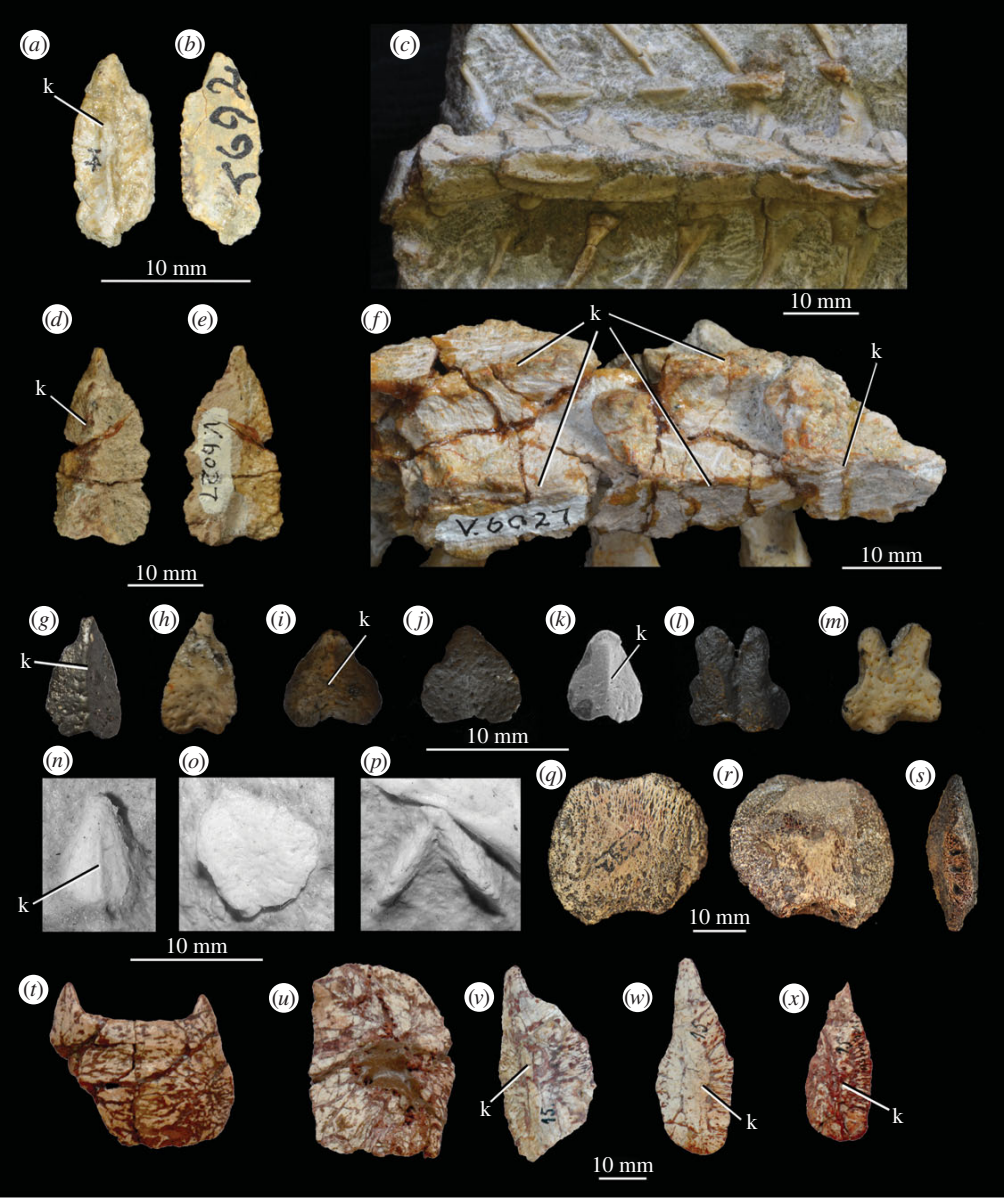
Osteoderms of euparkeriid taxa compared to other basal archosauriforms. *Euparkeria capensis* UMZC T.692 in (*a*) dorsal and (*b*)ventral view; *Euparkeria capensis* SAM-PK-6049A in (*c*) dorsal view; *Halazhaisuchus qiaoensis* IVPP V6027-9 in (*d*) dorsal and (*e*) ventral view; *Halazhaisuchus qiaoensis* IVPP V6027-2 in (*f*) dorsal view; *Osmolskina czatkowicensis* ZPAL RV/1339 in (*g*) dorsal and (*h*) ventral views; *Osmolskina czatkowicensis* ZPAL RV/1335 in (*i*) dorsal and (*j*) ventral views; *Osmolskina czatkowicensis* ZPAL RV/1338 in (*k*) dorsal view; Archosauriformes indet. ZPAL RV/1337 from Czatkowice 1 in (*l*) dorsal and (*m*) ventral views; different *Koilamasuchus gonzalezdiazi* MACN-Pv 18119 paramedian osteoderms in (*n*,*o*) dorsal, and (*p*) anterior or posterior view; putative osteoderm of *Erythrosuchus africanus* NHMUK PV R3592 in (*q*,*r*) dorsal or ventral and (*s*) medial/lateral/posterior/anterior view; *Rauisuchus tiradentes* osteoderms in dorsal view: BSPG AS XXV cervical osteoderm (*t*); BSPG AS XXV 97 anterior dorsal osteoderm (*u*); BSPG AS XXV 94 posterior dorsal osteoderm (*v*); BSPG AS XXV 121 caudal osteoderms (*w*,*x*). Image (*k*) courtesy of M. Borsuk-Białynicka and ZPAL; images (*n*–*p*) courtesy of M.D. Ezcurra; images (*t*–*x*) courtesy of S. Lautenschlager/O. Rauhut and the Linnean Society of London. k, median keel.

Two rows of anteriorly pointed paramedian osteoderms are seen in many crown pseudosuchians (e.g. *Ticinosuchus ferox* [82], *Rauisuchus tiradentes* [83]; figure 5). Most ornithodirans (e.g. *Herrerasaurus ischigualastensis* [74], *Silesaurus opolensis* [84]) and many stem archosaurs (e.g. erythrosuchids and proterosuchids [23]—although some controversy remains regarding erythrosuchids as discussed below; *Mesosuchus browni* [49]) lack dorsal osteoderms entirely. The osteoderms of proterochampsids differ in that they are rounded anteriorly, and form a single paramedian row [23,70], and those of phytosaurs differ in that they are rounded anteriorly and rugose [23]. The osteoderms of *Vancleavea campi* are similar in shape to those of euparkeriids, but cover much of the body rather than forming only paramedian rows [41]. A single osteoderm pertaining to *Koilamasuchus gonzalezdiazi* [22] is broadly similar to those of euparkeriids, but is more rounded anteriorly than those of *Euparkeria capensis*.

**Figure 6.**
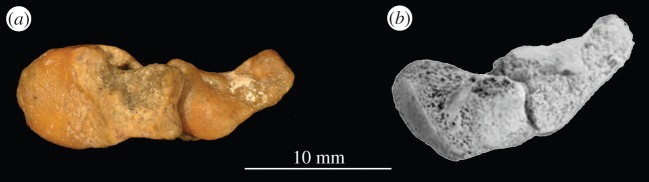
Articulated astragalus and calcaneum of (*a*) *Euparkeria capensis* UMZC T.692 and (*b*) *Osmolskina czatkowicensis* (astragalus ZPAL RV/811, calcaenum ZPAL RV/1253) in proximal view.

Non-archosauriform archosauromorphs and proterosuchids possess an ossified astragalocalcaneal canal, which is absent in all other archosauriforms [23,78,85]. A large anteriorly opening foramen (=anterior maxillary foramen of Modesto & Sues [86]) on the lateral surface of the maxilla at the base of the anterodorsal process (which is definitively homologous with that of *Euparkeria capensis*—see below and [23] pp. 66–67) is absent in all crown taxa except *Lotosaurus adentus* but is present in stem taxa excluding proterochampsids, some erythrosuchids, *Vancleavea campi* [23] and *Youngina capensis* [87]. The calcaneal tuber of stem archosaurs except proterochampsids is deflected by less than 20°, whereas that of crown archosaurs is deflected more than 50° posteriorly; like that of phytosaurs and proterochampsids, the calcaneal tuber of euparkeriids is deflected between 20° and 50° posteriorly (figure 6).

The medial wall of the vestibule in stem archosaurs is incompletely ossified, whereas that in many (but not all—*Silesaurus opolensis* and *Plateosaurus engelhardti* show partial ossification [23]) crown archosaurs is fully ossified [23,76]. All crown taxa lack pointed, recurved pterygoid teeth, though some crown taxa (e.g. *Eoraptor lunensis* [88]) do show blunt pterygoid teeth. *Erythrosuchus africanus* [69] and potentially herbivorous taxa such as *Trilophosaurus buettneri* [89] and derived rhynchosaurs such as *Hyperodapendon gordoni* [90] are the only stem taxa to lack pterygoid teeth entirely, although those of *Mesosuchus browni* are blunt (SAM-PK-6536).

##### 4.1.4.8

*Comments*. Euparkeriidae was divided into the subfamilies Euparkeriinae [59] and Dorosuchinae by Sennikov [63,91]. Euparkeriinae was composed of *Euparkeria capensis* and *Browniella africana*, but given that *Browniella africana* is considered synonymous with *Euparkeria capensis* this would render Euparkeriinae monospecific. Following the results of the phylogenetic analysis presented here, Dorosuchinae as conceived by Sennikov [63,91] is not monophyletic, as *Halazhaisuchus qiaoensis* and ‘*Turfanosuchus shageduensis*’ are recovered within Euparkeriidae whereas *Dorosuchus neoetus* is not, while the other two proposed members of this subfamily—‘*Wangisuchus tzeyii*’ and *Turfanosuchus dabanensis*—are considered a nomen dubium [28] and a pseudosuchian [23,46], respectively. Thus, employment of these subfamilies is not considered to be useful. A subclade is found within Euparkeriidae by the current analysis (grouping *Halazhaisuchus qiaoensis* and *Osmolskina czatkowicensis* to the exclusion of *Euparkeria capensis*), but given the low support and paucity of diagnostic characters for this clade, a clade name is not erected.

Borsuk-Białynicka & Evans [30, p. 240] further diagnosed Euparkeriidae as differing from erythrosuchids in the ‘lighter construction of the skeleton, relatively smaller skull, and generally more elongate cervical vertebrae (centrum length/depth usually around 1.4–1.6 instead of 0.4–1.0 in erythrosuchids)’. It is considered here that size and a lighter skeletal construction are not sufficiently clearly delimited characteristics to be included in the diagnosis. Regarding the length : height ratio of the cervical vertebral centra, it is found that this ratio varies greatly among archosauromorph taxa, with that of *Mesosuchus browni* (2.4 [49]) or *Proterosuchus alexanderi* (1.9—NMQR 1484) differing as much from that of euparkeriids as does the upper bound of the ratio of erythrosuchids. Furthermore, several crown taxa show cervical vertebrae as short as those of erythrosuchids (e.g. 0.8 in *Batrachotomus kupferzellensis* [92]). Given this fact and that cervical vertebral length appears to correlate strongly with ecomorphology (with shorter cervical vertebrae allowing a larger, more robust head), this feature is also not included in the current diagnosis.

#### 4.1.5

*Euparkeria* Broom, 1913 [26].

#### 4.1.6

*Type species*. *Euparkeria capensis* Broom, 1913 [26].

##### 4.1.6.1

*Diagnosis*. As for the type and only species.

#### 4.1.7

*Euparkeria capensis* Broom, 1913 [26].

##### 4.1.7.1

*Holotype*. SAM-PK-5867, largely complete skeleton including cranium.

##### 4.1.7.2

*Referred material*. AMNH 2238, partial postcrania; AMNH 2239, partial skull including jaws; AMNH 5548, caudal vertebrae; AMNH 19351, scrap of bone; GPIT 1681/2, partially disarticulated cranial and postcranial material of at least two individuals; SAM-PK-1100, fragments of pectoral and pelvic limbs and girdles and some vertebrae; SAM-PK-3427, partial forelimb; SAM-PK-5883, femur; SAM-PK-6047, forelimb; SAM-PK-6047A, skull, pelvic girdle and osteoderms; SAM-PK-6047B (same individual as SAM-PK-6047 and 6047B), pectoral and pelvic limbs and part of girdles, osteoderms (same individual as SAM-PK-6047 and 6047A); SAM-PK-6048, partial pectoral and pelvic limbs, girdles and vertebrae; SAM-PK-6049, pelvic region including sacral ribs and osteoderms; SAM-PK-6050, jaws and other cranial elements; SAM-PK-6557, scraps of bone; SAM-PK-7411, partial limb bones; SAM-PK-7696, most of skeleton including skull and braincase; SAM-PK-7699, pectoral limb and girdles, mandible; SAM-PK-7700, humerus and scapula; SAM-PK-7702, vertebrae; SAM-PK-7703, scraps of bone including vertebrae; SAM-PK-7704, forelimbs, vertebrae and ribs; SAM-PK-7705, partial hindlimb, ilium, osteoderms and vertebrae; SAM-PK-7706, forelimbs, vertebrae and ribs; SAM-PK-7709, caudal vertebrae; SAM-PK-7710, much of postcranial skeleton including girdles; SAM-PK-7712, limbs, girdles and other postcranial material; SAM-PK-7713, gastralia; SAM-PK-7868, forelimb, osteoderms, ribs; SAM-PK-7868, forelimb, osteoderms, ribs; SAM-PK-8309, pes; SAM-PK-10011, osteoderms, ribs, teeth; SAM-PK-10671, femur; SAM-PK-13664, palatine, pterygoid, ectopterygoid; SAM-PK-13665, anterior skeleton including skull; SAM-PK-13666, forelimb including manus, skull, caudal vertebrae; SAM-PK-13667, jaws and anterior postcrania; SAM-PK-K335, fragementary disarticulated postcrania; SAM-PK-K8050, three skeletons in large block; SAM-PK-K10010, forelimb, ribs, vertebrae; SAM-PK-K10012, partial forelimb; SAM-PK-K10548, scraps of bone; GPIT 1681/1, hindlimb and girdle, caudal vertebrae; UMZC T.692, block with remains of much of skeleton of two individuals.

##### 4.1.7.3

*Occurrence*. Single accumulation close to Aliwal North, Eastern Cape, South Africa, within the Burgersdorp Formation, Beaufort Group, within Subzone B of the *Cynognathus* Assemblage Zone (early Middle Triassic: early Anisian).

##### 4.1.7.4

*Diagnosis*. Euparkeriid distinguished from other taxa by the following autapomorphies: low peak on premaxilla projecting into external naris roughly at anteroposterior midpoint of naris, posterodorsal process (=postnarial process) of premaxilla primarily vertical, and rounded at its posterodorsal tip. Distinguished from *Osmolskina czatkowicensis* by possessing exoccipitals discrete from the opisthotics, a premaxilla lacking any slight overhang/downturn and which has a more vertical posterodorsal process (approx. 90° versus 50° in *Osmolskina czatkowicensis*) and is clearly attached by a facetted articulation to the maxilla (unlike the weak attachment in *Osmolskina czatkowicensis*) and not separated from it by an additional antorbital space (unlike in *Osmolskina czatkowicensis*), a tapered and posteriorly curved nasal process of the maxilla (contrasting with the subquadrangular process of *Osmolskina czatkowicensis*), and distinguished from *Osmolskina czatkowicensis* and *Halazhaisuchus qiaoensis* in lacking a tuber for the attachment of the m. triceps on the scapula.

##### 4.1.7.5

*Synonymy*. *Browniella africana* [27] is considered a junior subjective synonym of *Euparkeria capensis*, following Ewer [93]. No substantial morphological differences can be identified to distinguish the taxa, with the second pubic foramen and thinner, narrower pubis identified in *Euparkeria capensis* by Broom [27] being artefacts of damage (SAM-PK-5867) and also reflecting overall size difference between the specimens. The ventral part of the ischium of the holotype of ‘*Browniella africana*’ (SAM-PK-6047A) is damaged, and the greater constriction of the ilium near its middle in this taxon than in *Euparkeria capensis* identified by Broom [27] is thus also seemingly due to damage and/or unassessable.

#### 4.1.8

*Osmolskina* Borsuk-Białynicka and Evans, 2003 [29].

#### 4.1.9

*Type species*. *Osmolskina czatkowicensis* Borsuk-Białynicka and Evans, 2003 [29].

##### 4.1.9.1

*Holotype.* ZPAL R-I/77, anterior maxilla including anterodorsal process.

##### 4.1.9.2

*Referred material*. Catalogued material: 200 cranial and mandibular bones including braincase; 30 vertebrae from cervical, dorsal, sacral and caudal regions; five ilia; 30 limb bones. Other referred material: several hundred incomplete bones and teeth.

##### 4.1.9.3

*Diagnosis*. As for the type and only species.

#### 4.1.10

*Osmolskina czatkowicensis* Borsuk-Białynicka and Evans, 2003 [29].

##### 4.1.10.1

*Occurrence*. Karstic deposits of Czatkowice locality, Małopolska, Poland (late Early Triassic: late Olenekian).

##### 4.1.10.2

*Diagnosis (based on holotype only)*. The holotype maxilla is distinguished from that of *Euparkeria capensis* by possession of a subquadrangular nasal process (contrasting with the tapered process of *Euparkeria capensis*) which reaches less far posteriorly and is less posteriorly curved, and a relatively larger anterior maxillary foramen.

##### 4.1.10.3

*Further diagnosis based on referred material*. Distinguished from *Euparkeria capensis* by the features listed in the diagnosis of *Euparkeria capensis*. Distinguished from *Halazhaisuchus qiaoensis* and *Euparkeria capensis* by the possession of a relatively less elongated humerus with larger offset between the angles of its proximal and distal ends, and a strongly anterior position of the coracoid foramen. Distinguished from *Halazhaisuchus qiaoensis* by possession of a relatively mediolaterally thicker scapular blade and a less pronounced and less regularly circular attachment scar for the m. triceps on the scapula, lacking a clear central depression.

##### 4.1.10.4

*Comments*. Borsuk-Białynicka & Evans [30] additionally diagnosed *Osmolskina czatkowicensis* as differing from *Euparkeria capensis* in possessing a mandible that does not increase in depth posteriorly, and a more twisted femur (55° versus 32° offset between proximal and distal ends). It is considered here however that the mandible of *Osmolskina czatkowicensis* is not sufficiently well known to be certain as to whether it increased in depth posteriorly or not, and the offset between proximal and distal ends of the femur in *Euparkeria capensis* is relatively variable (e.g. approx. 45° in SAM-PK-6047B and 30° in SAM-PK-5883), whereas that in *Osmolskina czatkowicensis* is often not easily characterized owing to most femora being only partially preserved. Thus, these characteristics are excluded from the current diagnosis.

#### 4.1.11

*Halazhaisuchus* Wu, 1982 [62].

#### 4.1.12

*Type species*. *Halazhaisuchus qiaoensis* Wu, 1982 [62].

##### 4.1.12.1

*Diagnosis*. As for the type and only species.

#### 4.1.13

*Halazhaisuchus qiaoensis* Wu, 1982 [62].

##### 4.1.13.1

*Holotype* (from [28])*.* IVPP V6027, posterior three cervical and anterior three dorsal vertebrae in articulation with osteoderms and incomplete ribs (V6027-1), seven dorsal vertebrae in articulation with osteoderms (V6027-2), left (V6027-3) and right (V6027-4) scapulae, left (V6027-3) and partial right (V6027-4) coracoids, right humerus (V6027-5), ulna (V6027-6) and radius (V6027-7), an isolated left cervical rib (V6027-8), and an isolated median osteoderm (V6027-9). All material probably pertains to a single individual.

##### 4.1.13.2

*Occurrence*. Fugu County, Shaanxi Province, China, within the lower Ermaying Formation (Lower or Middle Triassic: late Olenekian to early Anisian).

##### 4.1.13.3

*Diagnosis*. Euparkeriid distinguished from other taxa by the following autapomorphies: (i) strongly pronounced tuber on the scapula, for attachment of the scapular head of the m. triceps, that is circular in outline when the scapula is in lateral view, with the apex of the tuber slightly depressed; (ii) pronounced muscle attachment scar on the scapula in the form of a depressed strip on the lateral surface of the blade running from anterodorsal to posteroventral, beginning at an abrupt kink in the anterior margin at around midlength of the blade (from Sookias *et al*. [28]). Similar m. triceps tubera in other taxa differ in shape, with that in *Osmolskina czatkowicensis* being less regularly circular and lacking a central depression, and those in crown archosaurs being teardrop shaped and lacking a depression (e.g. *Batrachotomus kupferzellensis* [92]). No similar muscle scar is seen in other euparkeriids or early archosauromorphs. Also differentiated from other euparkeriids in possessing epipophyses on the cervical vertebrae.

##### 4.1.13.4

*Comments*. Sookias *et al*. [28, p. 9], further diagnose *Halazhaisuchus qiaoensis* from other stem and crown archosaurs by a unique combination of characters ‘two rows of paramedian scutes with exposed surfaces at least twice as long as wide when articulated, tapering anterior processes and broad, rounded posterior margins, each having a longitudinal keel closer to the medial margin than the lateral one; large flattened flange projecting from the proximal part of the anterior margin of each cervical rib; presence of a tuber on the scapula for attachment of the scapular head of the m. triceps; presence of dorsal intercentra; epipophyses on cervical vertebrae’.

#### 4.1.14

‘*Turfanosuchus shageduensis*’ Wu, 1982 [62].

[Nomen dubium].

##### 4.1.14.1

*Holotype* (from [28])*.* IVPP V6028, mostly complete right mandible (V6028-1), six cervical vertebrae missing upper neural arches and neural spines (V6028-2), right scapula (V6028-3), coracoid (V6028-3), humerus (V6028-4), radius (V6028-7/8/9; note that the correct subnumbers for the radius, ulna and fibula are uncertain), ulna (V6028-7/8/9), femur (V6028-5), tibia (V6028-6) and fibula (V6028-7/8/9). All material probably pertains to a single individual.

##### 4.1.14.2

*Occurrence*. Jungar Banner, Nei Mongol Autonomous Region, China, from the lower Ermaying Formation (Lower or Middle Triassic: late Olenekian or early Anisian).

##### 4.1.14.3

*Comments*. Found to be undiagnostic beyond Archosauriformes indet. [28]. Placed within Euparkeriidae in the current phylogenetic analysis and thus potentially remains referable to the clade. Shows no scoring differences with *Halazhaisuchus qiaoensis*, but lack of autapomorphies uniting the taxa mean that they cannot be synonymized (see [28]).

## 5. Discussion

### 5.1. Euparkeriid monophyly and the composition of Euparkeriidae

#### 5.1.1. Support for Euparkeriidae

The results of the current analysis support recent work examining euparkeriid phylogeny (see [28,31]), with *Halazhaisuchus qiaoensis* and ‘*Turfanosuchus shageduensis*’ forming a clade with *Euparkeria capensis* and *Osmolskina czatkowicensis*, whereas *Dorosuchus neoetus* is alternatively placed outside Euparkeriidae as the sister taxon to Phytosauria + Archosauria or in a polytomy with Euparkeriidae and Phytosauria + Archosauria (see [31]; figures 2 and 3). Five unambiguous local synapomorphies support Euparkeriidae (table 1), but no unique apomorphies could be identified for the clade (see section Systematic palaeontology).

Euparkeriidae has relatively weak support (figures 2 and 3), and only one of its unambiguous synapomorphies is scorable for all taxa in the clade other than ‘*Turfanosuchus shageduensis*’ (which is not diagnosable—see [28] and below): distinct anterior process on paramedian osteoderms (table 1; character 393, state 1). The shapes of the osteoderms of *Euparkeria capensis* and *Halazhaisuchus qiaoensis* are very similar, with both showing anteroposteriorly elongated osteoderms with an anterior process (figure 5*a*–*f*), but similar osteoderms are also seen in crown taxa (e.g. *Rauisuchus tiradentes*—figuer 5*v*). Thus, osteoderm shape cannot be considered alone to be diagnostic of Euparkeriidae. Furthermore, the osteoderms referred to *Osmolskina czatkowicensis* (figure 5*i*–*k*) differ from those of *Euparkeria capensis* and *Halazhaisuchus qiaoensis* in that they are blunter anteriorly and less elongated. However, osteoderms more similar to those of *Euparkeria capensis* have been identified from the same deposits as those referred to *Osmolskina czatkowicensis* (figure 5*g*,*h*; alongside unusual, seemingly medially fused osteoderm pairs—figure 5*l*,*m*), and these may well pertain to the latter taxon.

The other four synapomorphies supporting Euparkeriidae can only be scored for *Euparkeria capensis* and *Osmolskina czatkowicensis*—the two most completely known euparkeriid taxa: presence of large anterolaterally opening foramen on anterolateral surface of maxilla (character 29, state 1); presence of four premaxillary teeth (character 6, state 1); presence of two foramina for cranial nerve XII in the exoccipital (character 115, state 0); and separately ossified interparietal (character 139, state 0). The distribution of these characters is discussed further in the Diagnosis.

#### 5.1.2. Support for clades within Euparkeriidae

**Figure 7.**
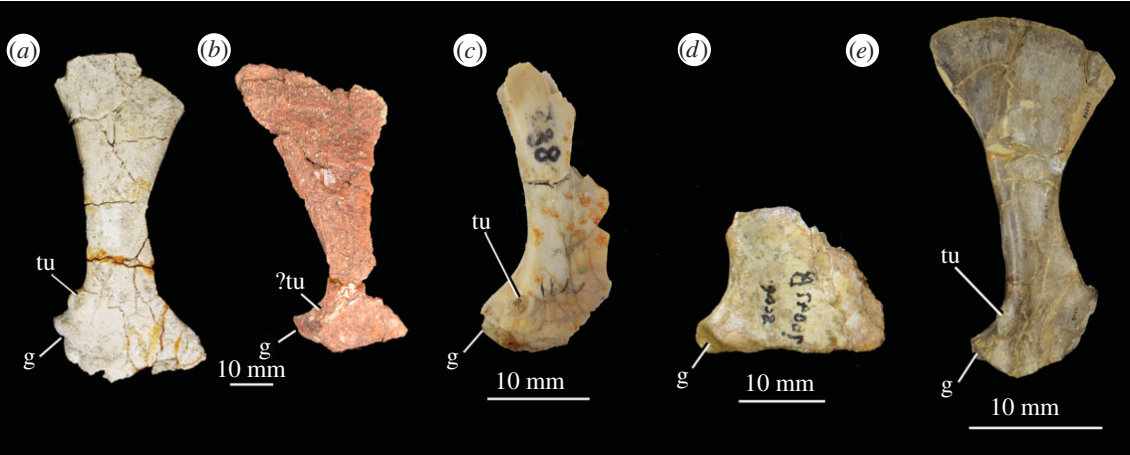
Scapulae of euparkeriids and a crown pseudosuchian showing m. triceps attachment tubera. (*a*) Left scapula and fragment of coracoid of *Halazhaisuchus qiaoensis* IVPP V6027-3 in lateral view; (*b*) left scapula of ‘*Turfanosuchus shageduensis*' IVPP V6028-3 in lateral view; (*c*) right scapula of *Osmolskina czatkowicensis* ZPAL RV/883 in lateral view (image mirrored for comparison); (*d*) proximal end of left scapula of *Euparkeria capensis* SAM-PK-6047B in lateral view (image mirrored for comparison); (*e*) right scapula of *Batrachotomus kupferzellensis* SMNS 80271 in lateral view. The same scale applied to (*a*) and (*b*). ?, uncertainty in identification; g, glenoid; tu, m. triceps attachment tuber.

A single unambiguous synapomorphy supports the clade within Euparkeriidae composed of *Osmolskina czatkowicensis*, *Halazhaisuchus qiaoensis* and ‘*Turfanosuchus shageduensis*’: presence of a pronounced tuber for the m. triceps on the scapula (character 220, state 1). This character is scored as present in ‘*Turfanosuchus shageduensis*’, but the tuber is damaged in this taxon (figure 7*b*) [28] and whether its morphology approached that of *Halazhaisuchus qiaoensis* and *Osmolskina czatkowicensis* is uncertain. The shape of the m. triceps tuber in *Halazhaisuchus qiaoensis* differs from that of pseudosuchians in that it is circular in lateral view, rather than teardrop shaped (figure 7*a*,*e*) [28]; although poorly preserved, that of *Osmolskina czatkowicensis* (figure 7*c*) is seemingly more similar to that of *Halazhaisuchus qiaoensis* than that of pseudosuchians. Given that this clade is also recovered by the Bayesian analysis, this topology appears to be the most robust interpretation of the currently available data. An additional clade comprising *Halazhaisuchus qiaoensis* and *Osmolskina czatkowicensis*, which is resolved only by the Bayesian analysis, is poorly supported, and it is not found by the parsimony analysis.

#### 5.1.3. The affinities of *Dorosuchus neoetus*

The position of *Dorosuchus neoetus* crownward of the euparkeriid clade, as the sister taxon to Phytosauria + Archosauria, is based on a single synapomorphy: absence of pterygoid teeth (character 168, state 1). Given that presence/absence of pterygoid teeth is a labile character (for example, some erythrosuchids lack palatal teeth, whereas some crown taxa possess them [46,69,88]), and that the Bayesian analysis did not resolve the node separating Euparkeriidae and *Dorosuchus neoetus*, it is felt that the more conservative interpretation—that the position of *Dorosuchus neoetus* relative to Euparkeriidae and more derived archosauriforms cannot currently be resolved (i.e. placement in a polytomy)—is preferable.

*Dorosuchus neoetus* is a highly incomplete taxon (79% missing data), and the assignment of all of the material referred to *Dorosuchus neoetus* is not certain. Not all material assigned to *Dorosuchus neoetus* is from the same block (the mandible and pterygoid are from a different block to the braincase, ilium and hind limb—see [7]), and only the ilium and hind limb (the holotype) were found in articulation [7]. While size, close proximity of discovery and compatible morphology were deemed enough to maintain referral of all material to a single taxon [7], uncertainty remains.

#### 5.1.4. The affinities of *Osmolskina czatkowicensis*

**Figure 8.**
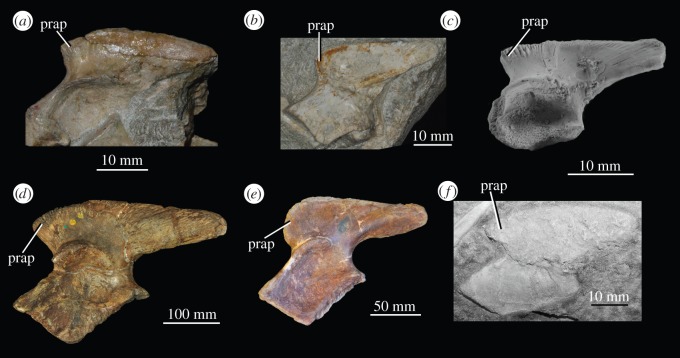
Ilia of euparkeriids, erythrosuchids and *Koilamasuchus gonzalezdiazi* showing degree of prominence of preacetabular processes. (*a*) left ilium of *Euparkeria capensis* SAM-PK-6049 in lateral view; (*b*) left ilium of *Euparkeria capensis* GPIT 1681/1 in medial view (image mirrored for comparison); (*c*) right ilium of *Osmolskina czatkowicensis* ZPAL RV/678 in lateral view; (*d*) right ilium of *Erythrosuchus africanus* NHMUK PV R3592 in lateral view (image mirrored for comparison); (*e*) left ilium of *Garjainia prima* PIN 951/8 in lateral view; (*f*) right ilium of *Koilamasuchus gonzalezdiazi* MACN-Pv 18119 in medial view (image mirrored for comparison). prap, preacetabular process.

The results presented here support the phylogenetic hypothesis presented, but not tested numerically, by Borsuk-Białynicka & Evans [29] that *Osmolskina czatkowicensis* is a euparkeriid, and contradicts those of Ezcurra *et al*. [22] (figure 1*c*) who found *Osmolskina czatkowicensis* to be placed further down the archosaur stem, stemward of erythrosuchids and *Koilamasuchus gonzalezdiazi*. In the analysis of Ezcurra *et al*. [22], the node separating *Koilamasuchus gonzalezdiazi* from *Osmolskina czatkowicensis* was supported by a single synapomorphy: well-developed preacetabular process of the ilium. However, while the development of the preacetabular process of *Osmolskina czatkowicensis* (figure 8*c*) is slightly less pronounced than in *Erythrosuchus africanus* [69] (figure 8*d*) or *Koilamasuchus gonzalezdiazi* [22] (figure 8*f*), the difference is marginal. Furthermore, *Euparkeria capensis* (SAM-PK-6049, GPIT 1681; figure 8*a*,*b*), which is the only other taxon scored by Ezcurra *et al*. [22] as poorly developed, is found closer to the crown than a number of taxa scored as well developed, whereas the preacetabular process of the erythrosuchid *Garjainia prima* (PIN 951/8; figure 8*e*) is no more strongly developed (and is more rounded) than that of *Euparkeria capensis* or *Osmolskina czatkowicensis*. As a result, the phylogenetic informativeness of this character is questionable.

**Figure 9.**
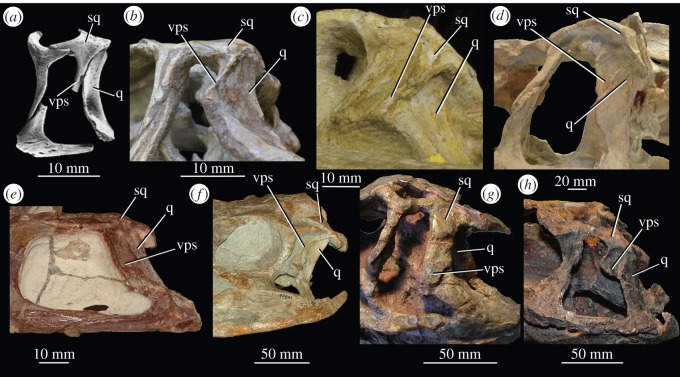
Squamosal and quadrate of euparkeriids, crown archosaurs and *Chanaresuchus bonapartei*, showing form of ventral ramus of squamosal. (*a*) Left squamosal articulated with quadrate, postorbital and jugal from different individuals (ZPAL RV/871, 872, 319 and 272, respectively) of *Osmolskina czatkowicensis* in lateral view; (*b*) posterodorsal region of left of skull in *Euparkeria capensis* SAM-PK-5867 in lateral view; (*c*) posterodorsal region of right of skull (image mirrored for comparison) of *Ornithosuchus longidens* NHMUK PV R2409 in lateral view; (*d*) posterodorsal region of right of skull (image mirrored for comparison) of *Batrachotomus kupferzellensis* (museum mount, squamosal and quadrate based on SMNS 80260 and SMNS 52970, respectively) in lateral view; (*e*) posterodorsal region of left of skull of *Chanaresuchus bonapartei* MCZ 4039 in lateral view; (*f*) posterodorsal region of right (image mirrored for comparison) of skull of *Plateosaurus engelhardti* SMNS 12949 in lateral view; (*g*) posterodorsal region of right (image mirrored for comparison) of skull of *Riojasuchus tenuisceps* PVL 3827 in lateral view; (*h*) posterior region of left of skull of *Herrerasaurus ischigualaestensis* PVSJ 407 in lateral view. Image (*a*) courtesy of M. Borsuk-Białynicka and Institute of Paleobiology of the Polish Academy of Sciences; images (*e–h*) courtesy of M.D. Ezcurra. q, quadrate; sq, squamosal; vps, ventral process of squamosal.

In the current analysis, only the distinction between presence and absence of a preacetabular process was included, whereas an attempt was made to delimit the *shape* of the preacetabular process in another character (see electronic supplementary material). The only scoring difference between *Euparkeria capensis* and *Osmolskina czatkowicensis* in the matrix of Ezcurra *et al*. [22] was that the posterior end of the squamosal does not extend posterior to the head of the quadrate in *Osmolskina czatkowicensis,* whereas it does in *Euparkeria capensis*; however, based on the reconstruction and attempt at articulating the relevant elements made by Borsuk-Białynicka & Evans [29,30] (figure 9*a*), this scoring is incorrect, with the condition in *Osmolskina czatkowicensis* appearing identical to that in *Euparkeria capensis* (SAM-PK-6047A; figure 9*b*). Furthermore, the postcranium and details of the cranium of *Osmolskina czatkowicensis* were unpublished at the time the scorings of the matrix of Ezcurra *et al*. [22] were conducted, and thus their dataset contained considerably more missing data for *Osmolskina czatkowicensis* than in the current matrix; thus, the current analysis is likely to have yielded a more accurate placement of the taxon.

All of the individual elements of *Osmolskina czatkowicensis* were collected in isolation [29,30,94], and were referred to a single taxon based on size, frequency and compatible morphology. This makes the referral controversial and potentially doubtful, as no synapomorphy set or autapomorphies were able to be identified to unite the material in a single taxon. Analysing individual elements separately might have circumvented this difficulty. However, given the paucity of information provided by each isolated element this approach was not considered to be worthwhile; even the holotype of *Osmolskina czatkowicensis* consists only of the anterior portion of a single maxilla (ZPAL R-I/77) [29] and thus analysing it alone would have been of little value.

#### 5.1.5. The affinities of *Halazhaisuchus qiaoensis* and ‘*Turfanosuchus shageduensis*’

Both *Halazhaisuchus qiaoensis* and ‘*Turfanosuchus shageduensis*’ are resolved within Euparkeriidae. Both taxa are highly incomplete (89% and 84% missing data, respectively), with the only cranial material consisting of a poorly preserved mandible of ‘*Turfanosuchus shageduensis*’, and osteoderms—an element important in diagnosing Euparkeriidae—are only preserved in *Halazhaisuchus qiaoensis*. Furthermore, as discussed by Sookias *et al*. [31], although differing in none of its scorings from *Halazhaisuchus qiaoensis*, ‘*Turfanosuchus shageduensis*’ lacks any autapomorphies which are able to diagnose it beyond Archosauriformes indet. Thus, although ‘*Turfanosuchus shageduensis*’ was included in the analysis presented here as a separate operational taxonomic unit, and was resolved within Euparkeriidae in a polytomy with *Osmolskina czatkowicensis* and *Halazhaisuchus qiaoensis*, it can only be tentatively considered a euparkeriid.

#### 5.1.6. The affinities of *Koilamasuchus gonzalezdiazi*

The holotype specimen of the enigmatic and highly incomplete taxon *Koilamasuchus gonzalezdiazi* [22] includes a number of osteoderms, one of which (figure 5*n*) resembles relatively closely those of *Euparkeria capensis* (figure 5*a*–*c*) and *Halazhaisuchus qiaoensis* (figure 5*f*) in that it is anteriorly tapered (but not to the same degree as in pseudosuchians such as *Turfanosuchus dabanensis*—IVPP V3237) and bears a median keel. Despite these similarities, the single keeled osteoderm of *Koilamasuchus gonzalezdiazi* is in fact more rounded anteriorly than those of *Euparkeria capensis* (SAM-PK-6047A), and it is more mediolaterally symmetrical. By contrast, the median keels of the osteoderms of *Euparkeria capensis* (SAM-PK-6047A) curve medially anteriorly and the anterior point also curves medially. However, the morphology of the keeled osteoderm of *Koilamasuchus gonzalezdiazi* osteoderm does closely resemble those assigned to *Osmolskina czatkowicensis* (ZPAL RV/1338; figure 5*k*) [94], although the latter are slightly shorter anteroposteriorly. The other preserved osteoderms of *Koilamasuchus gonzalezdiazi* (figure 5*o*,*p*) are rounded and lack a keel, contrasting markedly with those of euparkeriids.

The sole unambiguous synapomorphy supporting the sister relationship of *Koilamasuchus gonzalezdiazi* to doswelliids in the current analysis is a convex dorsal margin of the ilium with broadly rounded anterior and posterior ends; this contrasts with the ilia of *Euparkeria capensis* (GPIT 1681) and *Osmolskina czatkowicensis* (ZPAL RV/678), which have much straighter dorsal margins and less rounded corners (figure 8). Furthermore, while the anterior end of the ilium in *Euparkeria capensis* is consistently less rounded than that of *Koilamasuchus gonzalezdiazi*, the dorsal margin is variable in its convexity in *Euparkeria capensis* (e.g. that of SAM-PK-6049 is more convex than in GPIT 1681—figure 8*a*,*b*). The incomplete nature of *Koilamasuchus gonzalezdiazi* makes reaching certainty about its relationships difficult, but there is no strong evidence that it bears euparkeriid affinities.

### 5.2. Position of Euparkeriidae and the relationships of other stem archosaurs

#### 5.2.1. Erythrosuchidae

The placement of *Erythrosuchus africanus* closer to the crown than *Euparkeria capensis* that was uniquely recovered by Dilkes & Sues (figure 1*b*; see Previous phylogenetic work) [21] was supported by the following synapomorphies: ‘simple vertical or diagonal contact between the premaxilla and maxilla … a reversal to the absence of an anterior surangular foramen … mostly dichocephalous trunk ribs … and a non-bifurcate second sacral rib’ [21, p. 74]. The first synapomorphy corresponds in terms of scoring to character 29 in the present analysis, namely the presence/absence of an ‘anterolaterally opening foramen’ on the lateral surface of the maxilla (originally formulated by Modesto & Sues [86]), although whether the character of Dilkes & Sues [21] (reformulated from Dilkes [49]) was intended to capture the same variation as the character in the current matrix is unclear, with the distribution and delimitation of this character not discussed in detail by Dilkes [49] or Dilkes & Sues [21]. While this is not present in *Erythrosuchus africanus*, proterochampsids, doswelliids and most crown taxa, such a foramen is present in the erythrosuchid *Garjainia prima* (PIN 2394 5-1) and in the poposauroid *Lotosaurus adentus* [23], and the opening seen in most crown archosaur taxa between the premaxilla and maxilla may well have carried the same vessels and may be potentially homologous (character 13 in this analysis; see [23, pp. 61–63]).

**Figure 10.**
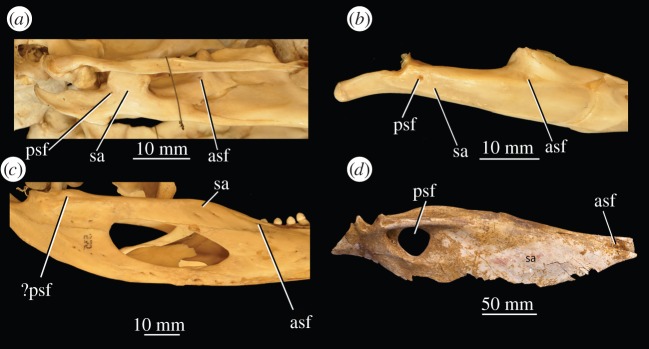
Mandibles of extant archosaurs and a lepidosaur showing surangular foramina. (*a*) Right mandible of *Anser* anser, specimen 162 of the Biological Sciences Collection of the University of Birmingham, Birmingham, UK (BSCUB), in lateral view; (*b*) right mandible of *Varanus salvator*, specimen 218 of the BSCUB, in lateral view; (*c*) left (image mirrored for comparison) mandible of *Alligator* sp., specimen 238 of the BSCUB, in lateral view; (*d*) left surangular of *Alioramus altai* IGM 100/1844 in lateral view (image mirrored for comparison). Image (*d*) courtesy of S. Brusatte. ?, uncertainty in identification; asf, anterior surangular foramen; psf, posterior surangular foramen; sa,surangular.

While an anterior surangular foramen is indeed present in *Euparkeria capensis*, *Osmolskina czatkowicensis, Proterosuchus fergusi* and *Prolacerta broomi*, and is apparently absent in taxa both crownward and stemward of *Proterosuchus* spp. and *Prolacerta broomi* in this dataset, the foramen is actually also present in very well-preserved fossil archosaurs (e.g. *Alioramus altai*—IGM 100/1844; figure 10*d*) and in modern birds (e.g. *Anser anser*—specimen 162 of the Biological Sciences Collection of the University of Birmingham, henceforth BSCUB; figure 10*a*), probably crocodilians (e.g. *Alligator* spp.—specimen 238 of the BSCUB; figure 10*c*), and in lepidosaurs (e.g. *Varanus salvator*—BSCUB 218; figure 10*b*). The feature is also not observable in some examples of *Prolacerta broomi* [86]. As such, preservation may easily obscure the presence of this foramen, especially as it is often positioned immediately adjacent to the surangular–dentary suture, meaning it is often obscured by damage, compression and/or displacement of the bones. For these reasons, this character has been excluded from this dataset, and caution is recommended in conclusions drawn based upon it.

The state ‘mostly dichocephalous trunk ribs’ is in fact also observed in *Euparkeria capensis*, with the majority of the dorsal ribs being dichocephalous [93]. Similarly, the second sacral rib of *Euparkeria capensis* is fundamentally non-bifurcate (SAM-PK-6049), with only a very slight depression present by comparison with the true bifurcation seen in other taxa (e.g. *Mesosuchus browni* [49]), as is also seen in *Erythrosuchus africanus* (NHMUK PV R3592).

Dilkes & Sues [21] used the presence of dorsal osteoderms to unite *Erythrosuchus africanus* with *Euparkeria capensis*, proterochampsids and doswelliids. As argued by subsequent authors [23,41,43], the two ‘osteoderms’ of *Erythrosuchus africanus* which were described by Gower [69] (figure 5*q*–*s*) are not identifiable with certainty as osteoderms. The trabecular nature of the bone even on the external surfaces (NHMUK PV R3592; figure 5*s*; osteoderms usually have cortical bone on their external surfaces [95]) and the scarcity of potential osteoderms count against the presence of osteoderms in the taxon. If not osteoderms, these fragments may simply be scraps of bone from other areas of the skeleton which have been weathered to broadly resemble osteoderms in shape. Furthermore, although this does not preclude presence of osteoderms in *Erythrosuchus africanus*, no osteoderms are currently identifiable in any other erythrosuchid (including a new, well-preserved specimen of *Shansisuchus shansisuchus* [96]). For these reasons, Nesbitt [23] is followed here in scoring dorsal osteoderms as absent in *Erythrosuchus africanus*.

Dilkes & Sues [21, p. 74] are correct in their statement that many of the characters historically used to place *Euparkeria capensis* more crownward than *Erythrosuchus africanus* have been problematic often owing to poor character definition. However, in the present analysis, the position of proterochampsids, doswelliids, *Vancleavea campi* and euparkeriids as more crownward than erythrosuchids is supported by the following unambiguous synapomorphies: loss of intertuberal plate on the parabasisphenoid; loss of postaxial intercentra (reversed in euparkeriids); reduction in expansion of distal condyles of femur; and presence of osteoderms.

#### 5.2.3. Doswelliidae

The placement of doswelliids in the current analysis, further from the crown than proterochampsids or euparkeriids, differs from some previous work which places doswelliids more crownward than euparkeriids, and either as the sister taxon to, in a polytomy with, or more derived than, proterochampsids (see Previous phylogenetic work). In the current analysis, one unambiguous synapomorphy which is scorable for doswellids (though only for *Jaxtasuchus salomoni*) supports proterochampsids and euparkeriids as more crownward than doswelliids: posterior expansion of the posterior part of the proximal portion of the fibula in lateral view.

Further unambiguous synapomorphies placing proterochampsids one node crownward to the clade which in this analysis is composed of doswelliids, *Vancleavea campi* and *Koilamasuchus gonzalezdiazi* (all only scorable for *Vancleavea campi*) are: posterior of squamosal extends posterior to head of quadrate; tibial and fibular articulations on astragalus continuous (not separated by gap/notch); and calcaneal tuber deflected 21–49° posteriorly (as opposed to less than 20°).

Of the unambiguous characters found to support doswelliids + proterochampsids as the sister taxon to Archosauria by Dilkes & Sues [21], the following were scorable in doswelliids: (i) loss of semilunar depression on parabasisphenoid; (ii) loss of posterior surangular foramen; (iii) loss of postaxial cervical intercentra; (iv) loss of dorsal intercentra; and (v) loss of deep excavation on mid-dorsal neural arches. Character state (i) is generally difficult to score (for example, it is not clearly visible in SAM-PK-5867, the holotype of *Euparkeria capensis*, despite the character state being clearly visible in a referred specimen, SAM-PK-7696), and a similar, potentially homologous feature may be present in crown taxa (e.g. *Saturnalia tupiniquim*—MCP 3845-PV). Regarding character state (ii), the posterior surangular foramen was scored by Dilkes & Sues [21] as absent in *Doswellia kaltenbachi* and the suprageneric taxon Proterochampsidae, but on close inspection of many well-preserved proterochampsid surangulars a foramen appears to be present (S. Nesbitt 2015, personal communication; although contested by the recent assessment of *Proterochampsa barrionuevoi* by Dilkes & Arcucci [43]), and a posterior surangular foramen is present in taxa phylogenetically bracketing stem archosaurs (lepidosaurs, crocodilians and birds; figure 10).

It was thus considered here that the apparent lack of the foramen in some stem archosaurs is probably a preservational artefact, and the posterior surangular foramen was not included as a character in the current analysis, with the variation between ‘large’ and ‘small’ foramina identified by Nesbitt [23] also found to be very difficult to reliably characterize. Regarding character states (iii) and (iv), although indeed absent in proterochampsids and doswelliids, as noted by Nesbitt [23] intercentra appear to be present only in some specimens of *Euparkeria capensis* (SAM-PK-6047A and SAM-PK-6047B; Gauthier [20] suggests that these individuals may represent juveniles, but evidence to support this suggestion is limited); *Halazhaisuchus qiaoensis* does however show large intercentra [28].

Regarding character state (v), while proterochampsids and doswelliids do, indeed, appear to lack the fossa at the base of the neural spine seen in *Euparkeria capensis* (UMZC T.692), *Mesosuchus browni* (SAM-PK-6046) and *Erythrosuchus africanus* [69], very similar, probably homologous structures are seen in most crown taxa (see [97]), and thus the importance of this character in placing proterochampsids and doswelliids nearer the crown than *Euparkeria capensis* is highly questionable; rather, reduction in development of vertebral laminae and fossae seems to be a unique feature of doswelliids and proterochampsids [97], possibly correlated with an aquatic lifestyle.

Ezcurra *et al*. [22] found two unambiguous synapomorphies supporting *Doswellia* + *Vancleavea* in a position crownward to proterochampsids: occipital condyle at the same level as (as opposed to anterior to) craniomandibular joint (only scored in *Doswellia kaltenbachi*); and astragalar posterior (=ventral) groove present (only scorable for *Vancleavea campi*). The variation referred to by the first character is very much continuous: e.g. *Chanaresuchus bonapartei*—MCZ 4037, *Erythrosuchus africanus*—BPI 5207 and *Euparkeria capensis*—SAM PK 5867 all have an occipital condyle anterior to the craniomandibular joint, but the offset between the two is progressively smaller. Scoring is also easily affected by post-mortem deformation: for example, on the left-hand side of the skull of *Doswellia kaltenbachi* (USNM 437574; which is scored as having the joint level with the occipital condyle [21]) the joint is slightly anterior to the occipital condyle, and on the right-hand side it is roughly level with it owing to distortion. Furthermore, the character reflects the entire morphology and mechanics of the skull (e.g. taxa with shallower, longer skulls, e.g. *Chanaresuchus bonapartei*—MCZ 4037, *Nicrosaurus kampffi*—NHMUK PV 42743, tend to have the occipital condyle further anterior to the craniomandibular joint than those with deeper, shorter skulls, e.g. *Euparkeria capensis*—SAM-PK-5867, *Erythrosuchus africanus*—BPI 5207) and its states are very labile (states 0 and 2 are found in non-crown taxa and crown pseudosuchians and avemetatarsalians, whereas 1 is found in some pseudosuchians and some non-crown taxa). This character's phylogenetic usefulness is thus highly questionable and it is excluded from the current analysis. The presence/absence of an astragalar posterior groove is highly labile both within and outside the crown, and its phylogenetic usefulness is thus also questionable, although the states are more easily delimited and the character is included in the current analysis.

Desojo *et al*. [42] found the following unambiguous synapomorphies to unite doswelliids with more crownward taxa to the exclusion of *Chanaresuchus bonapartei*: (i) absence of semilunar depression; (ii)ventral process of postorbital ends close to/at ventral margin of orbit; and (iii) ventral process of squamosal anteroventrally projected, constricting the infratemporal fenestra at midheight. While there is, indeed, no semilunar depression observable in most taxa crownward of this node, the presence/absence of the semilunar depression is problematic as a character (see above). Regarding character state (ii), on inspection of the taxa scored by Desojo *et al*. [42], character delimitation was found to be very difficult—for this reason and because it overlapped with character 67 of Nesbitt [23] (relative contributions of jugal and postorbital to postorbital bar), this character was excluded from this analysis.

**Figure 11.**
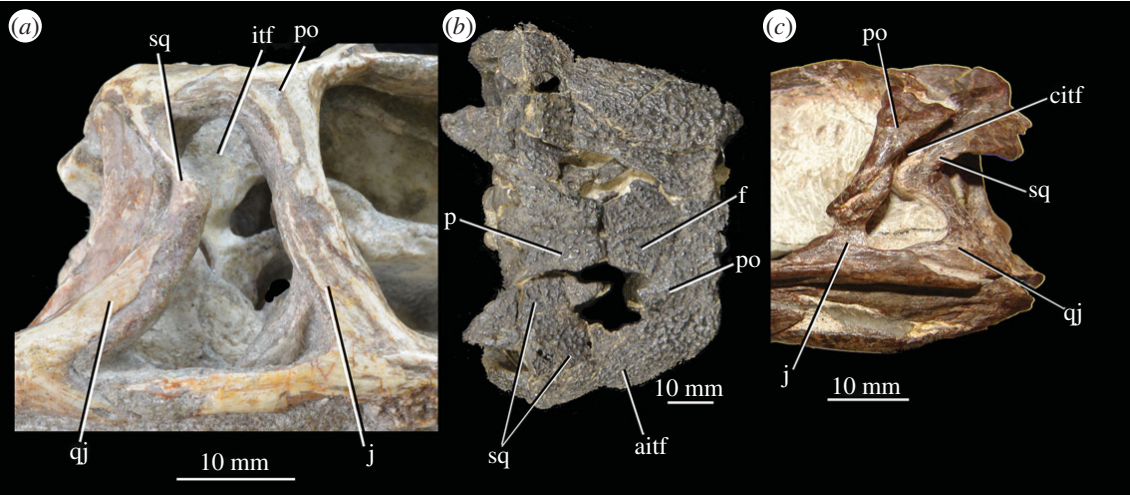
Temporal region of *Euparkeria capensis*, *Doswellia kaltenbachi* and *Gracilisuchus stipanicicorum* showing present, open infratemporal fenestra in *Euparkeria capensis*, absent infratemporal fenestra in *Doswellia kaltenbachi* and dorsally closed infratemporal fenestra in *Gracilisuchus stipanicicorum*. (*a*) *Euparkeria capensis* SAM-PK-5867 right-hand side in lateral view; (*b*) *Doswellia kaltenbachi* USNM 437574 both sides in dorsal view; (*c*) *Gracilisuchus stipanicicorum* MCZ 4117 left-hand side in lateral view. aitf, absent infratemporal fenestra; citf, closed infratemporal fenestra; itf, infratemporal fenestra; j, jugal; p, parietal; po, postorbital; qj, quadratojugal; sq, squamosal.

Character state (iii) was excluded from the current analysis, because it overlaps with character 52 of Nesbitt [23]; whereas the whole infratemporal fenestra is absent in *Doswellia kaltenbachi* (figure 11*b*), the morphology seen is quite distinct from the anterior projection seen in crown taxa (where the squamosal constricts the fenestra at midheight to form two triangular sections or separate fenestrae; figure 11*c*) and the two may not be homologous.

The node separating (Archosauria + (Proterochampsidae + Doswelliidae)) from *Euparkeria capensis* in the analysis of Schoch & Sues [44] was supported by the following synapomorphies scorable for doswelliids: (i) absence of posterior surangular foramen; (ii) no/shallow excavation on neural arches of mid-dorsals; (iii) loss of notch between clavicular articular facets on interclavicle; and (iv) presence of expansion on posterior part of interclavicle. Character states (i) and (ii) are discussed above. Contra Schoch & Sues [44], there is no notch between the articular facets for the clavicles on the interclavicle of *Euparkeria capensis*, and the interclavicle expands posteriorly to the same extent as in *Doswellia kaltenbachi* (SAM-PK-5867) [93]. The node separating *Doswellia kaltenbachi*, *Vancleavea campi*, *Chanaresuchus bonapartei* and crown taxa from *Euparkeria capensis* in the analysis of Parker & Barton [40] is supported only by absence of postaxial intercentra (see discussion above).

#### 5.2.4. Proterochampsidae

Contrary to the results of this analysis, many previous analyses have placed proterochampsids, or proterochampsids + doswelliids (see above), closer to the crown than is *Euparkeria capensis* (see Previous phylogenetic work). In the current analysis, six unambiguous synapomorphies unite Euparkeriidae with the crown to the exclusion of proterochampsids (table 1): (i) presence of a foramen on the medial side of the articular; (ii) expanded distal ends of cervical neural spines; (iii) expanded, flat distal ends of dorsal vertebrae; (iv) symmetrical distal end of fibula; (v) posterior corner of astragalus overlaps calcaneum more than anterior corner; and (vi) distinct anterior process on paramedian osteoderms. Conclusions regarding the position of Euparkeriidae with respect to proterochampsids should however remain tentative as synapomorphies (ii) and (iii) are related, the character states in the characters yielding synapomorphies (iv) and (v) are not easy to delimit, and synapomorphy (i) is easily obscured by preservation.

Characters reviewed by Sereno & Arcucci [35] which had been used previously to unite proterochampsids with Archosauria to the exclusion of *Euparkeria capensis* include loss of the postfrontal, opisthotic–exoccipital coossification, loss of postaxial intercentra and adjacent crural facets on the astragalus. Opisthotic–exoccipital coossification is however seen in *Erythrosuchus africanus* [75] and in *Osmolskina czatkowicensis* (ZPAL RV424), and preservation often makes assessment of the presence of intercentra difficult. Additionally, as noted by Gauthier [20], these characters are found in juveniles of crown taxa, and it is possible that *Euparkeria capensis* displays paedomorphic characteristics in accordance with its relatively small size. It is however unlikely that at least the larger specimens of *Euparkeria capensis* are juveniles, because palaeohistological evidence indicates that the bones were mature [98]. Were these characteristics found in euparkeriids owing to immaturity this would count against their usage in a phylogenetic analysis because it would be likely that these taxa would be mistakenly placed more basally than otherwise, but if owing to paedomorphosis in adult taxa then they remain informative.

Additional characters scorable for proterochampsids in subsequent analyses supporting a more crownward position for proterochampsids than *Euparkeria capensis* include: (i) subnarial process of premaxilla terminates ventral (not posterior) to external naris; (ii) metatarsal III greater than 40% of length of tibia [2]; (iii) posterior surangular foramen absent/extremely small [2,22,42,44]; (iv) loss of interparietal; (v) ventral acetabular wall projection at midlength of acetabulum (rather than anteriorly displaced); (vi) loss of hooked proximal end of metatarsal V (all [22]); (vii) reversal to more horizontal basisphenoid; (viii) loss of semilunar depression [21,22]; (ix) loss of vomerine teeth [22,42,44]; (x) no/ shallow excavation of mid-dorsal neural arches [21,22,42,44]; (xi) depression on descending process of postorbital [21,42]; (xii) shelf/ridge along dorsal margin of antorbital fenestra; and (xiii) phalanges of pedal digit V absent (both [21]).

Synapomorphies (iii) and (x) are discussed above, while the characters yielding synapomorphies (iv), (vi) and (xiii) are included in the current analysis but outweighed. The character yielding synapomorphy (i) (character 5 in the current analysis) was scored incorrectly for proterochampsids by Brusatte *et al*. [2] (*Chanaresuchus bonapartei*—PULR 07; [23]). Regarding synapomorphy (ii), the current analysis uses the formulation of Nesbitt [23] for this character, which is the relative length of the longest metatarsal being greater than 50% of the length of the tibia; this makes a clearer distinction between that in dinosauromorphs and that in all other stem and crown archosaurs, which was the original reason for the formulation of the character.

The character yielding synapomorphy (v) is excluded from the current analysis, but was incorrectly scored for *Euparkeria capensis* by Ezcurra *et al*. [22], with the ventralmost point of the iliac contribution to the ilium at approximately the anteroposterior midpoint of the acetabulum (figure 8*a*,*b*; as in *Stagonolepis robertsoni* [99] but contrasting with *Erythrosuchus africanus* [69]), whereas that in proterochampsids is anteriorly displaced (*Chanaresuchus bonapartei*—MCZ 4035). Regarding synapomorphy (viii), the vomers of *Chanaresuchus bonapartei* (the only proterochampsid included in the analyses) are denticulated [70,100].

Contra Ezcurra *et al.* [22] and Dilkes [49], the parabasiphenoid of *Chanaresuchus bonapartei* (PVL 4647) [70] and *Tropidosuchus romeri* (PVL 4604) [50] is not horizontally orientated as in *Proterosuchus fergusi* (BP/1-3993) [87] or *Mesosuchus browni* (SAM-PK-6536) [49]. Ezcurra *et al*. [22] score the proximal end of metatarsal V as not being hooked in proterochampsids, but Nesbitt [23] and Arcucci [70] both consider the proximal end to be hooked; in the absence of the opportunity to extensively inspect the material personally, the latter two authors were followed here. The character yielding synapomorphy (x) was excluded from the current analysis as the original morphological feature in *Yonghesuchus sangbiensis* by Wu *et al*. [101] is, in fact, a misinterpretation of part of the squamosal as belonging to the postorbital [46]. That yielding synapomorphy (xi) was also excluded as delimitation was problematic (a pronounced ridge is not apparent in all specimens of, for example, *Chanaresuchus bonapartei*, nor in all proterochampsid taxa [70], and a blunt ridge is seen in *Euparkeria capensis*—SAM-PK-5867).

#### 5.2.5. Crown Archosauria

This analysis concurs with most recent work on stem and early archosaur phylogeny in that it places *Euparkeria capensis* and other euparkeriids close to the base of, but outside, Archosauria. However, it contrasts with the placement of *Euparkeria capensis* within the crown, as the sister taxon to Ornithosuchidae + Ornithodira (within ‘Ornithosuchia’), uniquely found by Gauthier [20] (figure 1*a*; see Previous phylogenetic work), and with Broom [26,27] who suggested an affinity between *Euparkeria capensis* and ornithosuchids. Given that this position has not been found by any more recent analysis, these results are not discussed further here, but the characters supporting placement outside the crown are discussed in detail in the electronic supplementary material.

#### 5.2.6. Vancleavea campi

The placement of *Vancleavea campi* as the sister taxon to doswelliids in the current analysis agrees with the placement of several previous analyses [22,43,44] (Parker & Barton [40] find *Vancleavea campi* in a polytomy with *Doswellia kaltenbachi*; figure 1*c*), but contrasts with Desojo *et al*. [42], who place *Vancleavea campi* further down the stem than doswelliids (and also than *Erythrosuchus africanus* and *Euparkeria capensis*), and the findings of Nesbitt *et al*. [41] and Nesbitt [23] (figure 1*d*), who placed *Vancleavea campi* further down the stem than proterochampsids and *Euparkeria capensis*. The phylogenetic position of *Vancleavea campi* is not directly relevant to euparkeriid phylogeny, but is discussed further in the electronic supplementary material.

### 5.3. Parsimony versus Bayesian analysis results

Results from parsimony and Bayesian analyses were broadly very similar, but more, and larger polytomies were found with the Bayesian analysis (table 2). This may reflect the effective downweighting of homoplastic characters in the Bayesian analysis caused by the assignment of faster rates of evolution to these characters (see [54]). Which of the two phylogenies is more accurate is fundamentally uncertain given the current state of knowledge regarding the application of these methods to morphological data. Recent work has indicated, based on simulated data, that Bayesian analyses may yield higher accuracy [102]. However, there is controversy as to the justifiability of the necessary input parameters for the models used in Bayesian analysis [103,104], and a lower stratigraphic fit has been found for Bayesian analyses of fossils [105].

Interestingly, the Bayesian analysis presented here does not show greater resolution than the parsimony analysis, contra many previous fossil studies [104], and is thus more conservative. While a continued complaint regarding Bayesian phylogenies has been that they inflate clade support values [106], it is felt here that it is not meaningful to compare bootstrap and posterior probability values directly, with a more useful approach being simply to compare relative support for clades within each tree; posterior probabilities should not be interpreted as realistic probabilities that clades exist, but rather relatively in relation to each other within the tree. However, caution may be warranted regarding the relative probabilities assigned to placement of highly incomplete taxa, as these specifically may be inflated by Bayesian methods [107]. Overall, given the lack of knowledge regarding the effectiveness of both methods, it is felt that a conservative approach, treating polytomies in both consensus trees as areas of major uncertainty, is the most prudent.

### 5.4. Euparkeriidae and the evolution and spread of the archosaur body plan

The current analysis confirms recent work indicating that Euparkeriidae consisted of more than a single genus [28], but also confirms findings that a ‘euparkeriid grade’ of stem archosaur morphological features appears to have existed, including Euparkeriidae, *Dorosuchus neoetus* [31] and, in some respects, proterochampsids. This grade, including Euparkeriidae, shows a combination of crown and stem archosaur features. For example, the limbs are more adapted for semi-upright, cursorial locomotion than those of stem taxa such as erythrosuchids and proterosuchids, but do not yet show the adaptions to fully upright cursorial locomotion seen in crown taxa. Like crown taxa [23,92] and proterochampsids [23,70], the femora of *Euparkeria capensis*, *Osmolskina czatkowicensis* and *Dorosuchus neoetus* are sigmoid and gracile, the fourth trochanter is reduced and ventrally displaced (in comparison with, for example, *Erythrosuchus africanus* [69]), and the proximal and distal ends are more extensively ossified than in erythrosuchids or proterosuchids [7,23]. However, the femora are not fully adapted for upright locomotion (see [18]), lacking either the fully dorsal femoral articulation with the acetabulum seen in crown pseudosuchians [23,92] or the medially directed femoral head seen in ornithodirans [23,108].

Similarly, the ilia of euparkeriid taxa and *Dorosuchus neoetus*, along with those of many basal archosaurs (e.g. *Gracilisuchus stipanicicorum*—MCZ 4118; *Batrachotomus kupferzellensis* [92]), and erythrosuchids [69], doswelliids [21] and most proterochampsids [70], show a relatively short, but present preacetabular process, contrasting with the absent process in more basal taxa (e.g. *Mesosuchus browni* [49], *Proterosuchus alexanderi*—NMQR 1484) and longer process in more derived crown archosaurs (e.g. *Herrerasaurus ischigualastensis* [109]). *Osmolskina czatkowicensis* and *Euparkeria capensis* also share a straight-sided preacetabular process with the dorsal and anterior edges meeting at an angle greater than 70°, as in proterochampsids [70] and some phytosaurs [110], but contrasting with the more acute angle in many crown taxa (e.g. *Gracilisuchus stipanicicorum*—MCZ 4118). The preacetabular process of *Dorosuchus neoetus* is rounded, approaching more closely the morphology of some erythrosuchids (e.g. *Garjainia prima*—PIN 951/8). Increased length of the preacetabular process is thought to be associated with movement to increasedly upright locomotion [111], and thus euparkeriids again appear to show an intermediate morphology between that of ‘sprawling’ and ‘erect’ taxa.

Based on the results of character state optimization (see above), it is reasonable to conclude that the ancestor of crown Archosauria and Phytosauria was relatively small, gracile and cursorial (e.g. fourth trochanter present; femoral distal condyles not projecting markedly beyond shaft), terrestrial and carnivorous—thus, very similar overall to a euparkeriid or *Dorosuchus neoetus*. Proterochampsids also show a similar locomotor morphology to the euparkeriid taxa and *Dorosuchus neoetus*, but other aspects of their morphology, especially their elongated and flattened skull, appear to indicate a semiaquatic habitus [70]. *Proterochampsa barrionuevoi* (PVL 2063) also possesses pronounced dermal ornamentation, which is also seen in phytosaurs (*Smilosuchus gregorii*—UCMP 27200) and crocodilians [112]. *Dongusuchus efremovi* also shows a gracile femoral morphology, and is also placed close to the base of the archosaur crown (although its incompleteness means that it greatly reduces resolution, and its placement is very uncertain). Although *Dongusuchus efremovi* was excluded from the tree onto which character states were optimized owing to the reduction in resolution it created, the morphology of the taxon certainly does not weaken the hypothesis that the ancestral archosaur was cursorial and gracile, as discussed by Niedźwiedzki *et al*. [48].

Indeed, the optimization of ecological characters onto the phylogeny is of some interest precisely because several other taxa on the archosaur stem show aquatic adaptations (proterochampsids, doswelliids, *Vancleavea campi*), and phytosaurs are also aquatic, so an aquatic ancestor of Phytosauria + Archosauria would not be unexpected. In terms of its body plan, however, *Euparkeria capensis* and other euparkeriids certainly more closely approach the crown archosaur taxa which went on to radiate on land than they do doswelliids or phytosaurs, and the development of the ‘euparkeriid grade’ body plan can in this light be potentially looked upon as a key innovation which allowed radiation into terrestrial, cursorial niches.

Euparkeriidae shows a wide geographical spread, being found across northern and southern parts of the supercontinent Pangaea, potentially reflecting limited barriers to dispersal in the Early and Middle Triassic (see [113]). Furthermore, euparkeriid-like morphology appears to have been yet more widespread globally, with the similar locomotor grade of proterochampsids and *Dorosuchus neoetus* extending the range of this morphology to South America and Russia, respectively. Small, cursorial, carnivorous taxa similar to *Euparkeria capensis* thus appear to have made up a minor but ubiquitous part of Middle Triassic ecosystems, coexisting with crown archosaurs (e.g. gracilisuchids [46], *Dongusuchus efremovi* [48]) and relatively small-bodied carnivorous therapsids (e.g. *Cynognathus* [114]) which probably filled similar ecological niches.

## 6. Conclusion

Overall, the phylogenetic work presented here helps to clarify our understanding of the early evolution and rise of Archosauria. The existence of a globally distributed euparkeriid clade is supported, thus in itself representing a successful and important radiation within the wider radiation of archosauromorphs and archosauriforms seen during the Early and Middle Triassic. A gracile, cursorial morphology intermediate between that of many stem taxa and the fully erect stance seen in crown taxa was yet more widespread, with *Dorosuchus neoetus* potentially representing an independent lineage with very similar morphology to that displayed by euparkeriids.

Even if support for euparkeriid monophyly is not strong, the broader phylogenetic position of euparkeriids and of *Dorosuchus neoetus* appears to be relatively stable, and it can be confidently concluded that a gracile, terrestrial, cursorial and small-bodied morphology is very likely to have been possessed by the ancestor of Archosauria. This morphology may have underlain subsequent archosaur success and radiation, allowing dinosaurs to reach their exceptional sizes, and Archosauria to become the most speciose and one of the most ecomorphologically diverse terrestrial vertebrate clades.

## Supplementary Material

Supplementary Information.pdf - supplementary figures, text and references.

## Supplementary Material

Matrix and character lists.xlsx - phylogenetic matrix and character lists.

## Supplementary Material

Matrix.nex - phylogenetic matrix as nexus file.

## Data accessibility

All data have been made available on Dryad (http://dx.doi.org/10.5061/dryad.b449r) and in the form of the electronic supplementary material with the manuscript.
