# Supplementary material for: The relationships of the Euparkeriidae and the rise of Archosauria
Source: R Soc Open Sci. 2016 Mar 23;3(3):150674. doi: 10.1098/rsos.150674 (PMC4821269; doi:10.1098/rsos.150674)
Supplement: Supplementary Information.pdf - supplementary figures, text and references. [file rsos150674supp1.pdf]

Supplementary Information

Cladistic analysis including character 93 – ridge separating palatal flange of pterygoid and pterygoid neck

Number of most parsimonious trees: 24

Tree length: 1339

Consistency index: 0.356

Retention index: 0.687

See Fig. S1

Cladistic analysis including *Dongusuchus efremovi*

Number of most parsimonious trees: 192

Tree length: 1334

Consistency index: 0.357

Retention index: 0.686

See Fig. S2

Description and explanation of new characters

[1] 12. Premaxilla, procumbency: (0) absent; (1) present, so that majority of premaxilla ventral border is exposed; (2) present but slight, such that at most anteriormost two teeth project beyond maxilla; (3) present, but anterior to alveoli (see Fig. S11).

This character differs from character 8, which captures whether the premaxilla is downturned, in that it refers simply to the degree of procumbency of the premaxilla beyond the dentary. *Youngina capensis* (Camp 1945) and *Erythrosuchus africanus* (Gower 2003) show a strongly procumbent maxilla such that the majority of the premaxillary alveoli are exposed ventrally, but its premaxilla is not markedly downturned. The premaxilla of proterosuchids (Ezcurra et al. 2015) and ornithosuchids (Walker 1964; Bonaparte 1971) is strongly procumbent and strongly downturned. The premaxilla of some gracilisuchids (Romer 1972a; Wu and Russell 2001) is strongly procumbent but not downturned. The premaxilla of proterochampsids (Sill 1967; Romer 1971; Arcucci 1991; Arcucci 2011), some pseudosuchians (in the current dataset *Dromicosuchus grallator* – Sues et al. 2003, *Yonghesuchus sangbiensis* - Wu et al. 2001 - and *Qianosuchus mixtus* – Li et al. 2006), and some theropod (in the current dataset *Herrerasaurus ischigualastensis* – Sereno and Novas 1994 - and *Eoraptor lunensis* - Sereno et al. 2012) dinosaurs is slightly procument and not downturned. Aetosaurs (Walker 1961; Schoch 2007) show a strongly procumbent premaxilla, but the alveoli are set posteriorly such that much of the procumbence is anterior to the alveoli. The erythrosuchids *Garjainia prima* (Ochev 1958; Ochev 1981) and *Shansisuchus shansisuchus* (Wang et al. 2013) show a slightly downturned premaxilla, but a strongly and slightly procumbent premaxilla respectively. Other taxa, including *Euparkeria capensis* (SAM-PK-6047A), do not show a procumbent premaxilla; *Osmolskina czatkowicensis* is not scored for this character because of uncertainty regarding the exact position of the dentary

with respect to the premaxilla when in articulation.

[2] 33. Skull, dermal bones, irregular pit-and-ridge ornamentation: (0) absent; (1) present (see Fig. S12).

Vancleavea campi (Nesbitt et al. 2009), phytosaurs (e.g. *Parasuchus hislopi* - ISI R 42; *Smilosuchus gregorii* – UCMP 27200; *Pseudopalatus pristinus* – Mehl 1928), the doswelliid *Doswellia kaltenbachi* (Dilkes and Sues 2009;), the proterochampsid *Proterochampsa barrionuevoi* (Sill 1967) and some crocodylomorphs (not included in this dataset; including extant taxa – Buffrénil et al. 2014) show irregular pit-and-ridge ornamentation on the dermal bones of the skull. This character appears to be associated with an aquatic habitus.

[3] 81. Jugal, ridge/expansion projects ventral to rest of ventral margin at base of postorbital bar: (0) absent; (1) present (see Fig. S13).

The erythrosuchids *Garjainia prima* (PIN 2394-5) and *Erythrosuchus africanus* (Gower 2003) show an expansion of the jugal projecting below the rest of the ventral margin of the jugal immediately ventral to the base of the postorbital bar. In *Erythrosuchus africanus* this ventral expansion is also associated with a ridge on the jugal; in the erythrosuchid *Garjainia madiba* (Gower et al. 2014; not included in this dataset) this expansion is associated with a very pronounced boss. The erythrosuchid *Shansisuchus shansisuchus* lacks this expansion (Wang et al. 2013). *Rauisuchus tiradentes* (Lautenschlager and Rauhut 2014) and *Proterosuchus* (e.g. NMQR 1484; BP/1/4016) also show a similar ventral expansion. Some other taxa show a ventral expansion in a slightly different position (e.g. anteriorly - *Batrachotomus kupferzellensis*, Gower 1999), or a more gradual, less pronounced expansion in a similar position (e.g. *Herrerasaurus ischigualaestensis* – Sereno and Novas 1994).

[4] 191. Cervical ribs, anterolateral flange: (0) absent; (1) present; (2) present and elongated into process (see Fig. S14).

Stem archosaur taxa (e.g. *Protorosaurus speneri* - Gottmann-Quesada and Sander 2009; *Garjainia prima* – PIN 2394/5 ; *Euparkeria capensis* – SAM-PK-5867; *Erythrosuchus africanus* – Gower 2003; Fig. S14E,F) and ornithodires (e.g. *Silesaurus opolensis* – Dzik 2003; *Heterodontosaurus tucki* – SAM-PK-K1332; *Eoraptor lunensis* – Sereno et al. 2012; S5G) lack an anterolateral flange on the cervical ribs, with these taxa instead showing an anterior process developed to varying degrees. This process is flattened and widened to form a clear flange in many pseudosuchians (e.g. *Batrachotomus kupferzellensis* – Gower and Schoch 2009; *Gracilisuchus stipanicicorum* – Romer 1972a; S5C,D) and in *Halazhaisuchus qiaoensis* (IVPP V6027-9; S5A,B).

[5] 268. Ilium, anterior (=preacetabular, =cranial) process, shape: (0) rounded; (1) approximately straight-sided with angle >70°; (2) approximately straight-sided with angle <70°; parallel-sided, blunt end (see Fig. S14).

This character attempts to capture the variation within the shape of the preacetabular process in taxa where it is present. *Dorosuchus neoetus* (PIN 1579) and the erythrosuchids *Garjainia prima* (PIN 951/8) and *Shansisuchus shansisuchus* (Young 1964) show a rounded preacetabular process in lateral view. Many other stem taxa (e.g. *Erythrosuchus africanus* – Gower 2003; *Euparkeria capensis* – GPIT 1681/1), including some phytosaurs (e.g. *Parasuchus hislopi* – Chatterjee 1978), and some crown pseudosuchians (e.g. *Ornithosuchus longidens* – Walker 1964; *Batrachotomus kupferzellensis* - Gower and Schoch 2009), show a straight-sided preacetabular process (i.e. where the anteroventral and dorsal margins of the ilium intersect to form an angle) but with an angle of intersection of the two sides >70°. Many crown pseudosuchians (e.g. *Gracilisuchus stipanicicorum* – Romer 1972a; *Aetosaurus ferratus* – Schoch 2007) and ornithodires (e.g. *Eoraptor lunensis* – PVSJ 512; *Silesaurus opolensis* – Dzik 2003) show a

narrow, pointed preacetabular process, with the angle of intersection <70°. Some theropod (e.g. *Herrerasaurus ischigualastensis* - Novas 1993; *Coelophysis bauri* – Colbert 1989) and ornithschian dinosaurs (e.g. *Heterodontosaurus tucki* – Santa Luca 1980), and the dinosauromorph *Marasuchus lilloensis* (Sereno and Arcucci 1994) show a preacetabular process where the sides are approximately parallel and connected by a blunt end.

Supplementary discussion

Placement of euparkeriids outside of crown Archosauria

The current analysis contrasts with the placement of *Euparkeria capensis* within the crown, as the sister taxon to Ornithosuchidae+Ornithodira (within "Ornithosuchia"), uniquely found by Gauthier (1986; Fig. 1A; see *Previous phylogenetic work* in main text) and with Broom (1913a,b) who suggested an affinity between *Euparkeria capensis* and ornithosuchids. The affinity proposed by Broom (1913a,b) was however based only on overall unspecified similarity in skull shape, and the encroachment of the distal end of the ventral ramus of the squamosal and the dorsal ramus of the quadratojugal into the lateral temporal fenestra (1913a). No apomorphic features of the skull were identified to unite *Euparkeria capensis* with *Ornithosuchus longidens* to the exclusion of other crown taxa however. Furthmore, the degree of encroachment by the squamosal and quadratojugal differs substantially between the taxa, with the encroachment in *Ornithosuchus longidens* far more substantial, and approaching the condition in other pseudosuchians (e.g. gracilisuchids – Butler et al. 2014) where the squamosal contacts the postorbital bar. The characters used by Gauthier (1986) to place *Euparkeria capensis* within Archosauria were: (1) squamosal reduced and descending ramus gracile; (2) centra steeply inclined at least in first four postatlantal cervicals; (3) modifications of hindlimb and girdle correlated with semierect gait; (4) ventral flange of astragalus absent; (5) crocodile-reversed ankle joint (peg on calcaneam, socket on astragalus); (6) pedal digit V with less than four phalanges.

While the ventral ramus of the squamosal in *Euparkeria capensis* (SAM-PK-5867; Fig. 8B) and of *Ornithosuchus longidens* (Walker 1964; Fig. 8C) is indeed more gracile than that of many pseudosuchian taxa (e.g. *Batrachotomus kupferzellensis* – Gower 1999, *Prestosuchus chiniquensis* – Barberena 1978; Fig. 8D) and stem archosaurs (e.g. *Chanaresuchus bonapartei* [97]; Fig. 8E), that of the ornithosuchid *Riojasuchus tenuiceps* (Bonaparte 1971; Fig. 8G) is far more robust. Furthermore, the ventral ramus of the squamosal in *Euparkeria capensis* is nowhere near as thin relative to its length as in saurischian dinosaurs (Fig. 8F), and the character delimitation employed here (wider or narrower than one quarter of its length, following Nesbitt 2011, Yates 2003, Langer and Benton 2006) better captures the variation in this feature.

The "reduced" and "gracile" nature of the squamosal in *Euparkeria* reflects the overall gracile morphology of the skull and its size (with a thin ventral ramus of the squamosal similarly found in *Coelophysis bauri* – Colbert 1989) contrasting with the wide ramus in the larger *Herrerasaurus ischigualastenis* (Sereno and Novas 1994; Fig. 8H), and does not represent a particular novel feature of the element, meaning that its phylogenetic usefulness is highly questionable. Regarding the "steeply inclined" nature of the centra of the first four postatlantal cervical vertebrae, delimitation and characterization of the variation referred to was found to be difficult, with no clear demarcation between the state seen in *Euparkeria capensis* and that in, for example, *Erythrosuchus africanus* (Gower 2003).

Modifications correlated with a semierect gait is, as made clear by Sereno and Arcucci (1990), an extremely loosely defined character. Even if it is broken down into several more specific characters, such modifications are also found in all pseudosuchian taxa, and the only taxa in or immediately outside the crown to lack these modifications are the aquatic phytosaurs; on the stem, proterochampsids (e.g. *Chanaresuchus bonapartei* – Romer 1972b) also show the derived state. These modifications are thus certainly not a feature which convincingly unites *Euparkeria capensis*

with ornithodires to the exclusion of pseudosuchians. The ankle characters used by Gauthier (1986) to unite *Euparkeria capensis* with "ornithosuchians" were thoroughly discussed by Sereno and Arcucci (1990). After inspection of the specimens and based on the description of Cruickshank (1979), these authors came to the conclusion, which is followed here, that *Euparkeria capensis* (like *Osmolskina czatkowicensis*) does not in fact show an ornithosuchian-like "crocodile reversed" ankle joint, nor indeed any other features of the ankle which unite it with either major archosaur lineage (see Fig. 11A).

Whilst *Euparkeria capensis* does (as outlined in Sereno and Arcucci 1990), like ornithosuchids, possess three phalanges on digit V, this character appears to be very labile, with proterosuchids (Cruickshank 1972) and erythrosuchids (Young 1964) showing three phalanges, proterochampsids possessing a single phalanx (Nesbitt 2011), and some pseudosuchians showing fewer than four (e.g. *Neoaetosauroides* shows two – Bonaparte 1971). Furthermore, on inspection of the material (SAM-PK-8309), the condition in *Euparkeria capensis* is not absolutely clear due to damage.

Phylogenetic placement of *Vancleavea campi*

The placement of *Vancleavea campi* as the sister taxon to doswelliids in the current analysis agrees with the placement of several previous analyses (Ezcurra et al. 2010; Dilkes and Arcucci 2012; Schoch and Sues 2013; Parker and Barton 2008 find *Vancleavea campi* in a polytomy with *Doswellia kaltenbachi*; Fig. 1C), but contrasts with Desojo et al. (2011) who place *Vancleavea campi* further down the stem than doswelliids (and also than *Erythrosuchus africanus* and *Euparkeria capensis*), and that found by Nesbitt et al. (2009) and Nesbitt (2011; Fig. 1D) who placed *Vancleavea campi* further down the stem than proterochampsids and *Euparkeria capensis*. This placement in the current analysis is however only supported by two unambiguous synapomorphies: (1) presence of irregular pit and ridge ornamentation on dermal skull bones; (2) absence of lateral mandibular fenestra. Synapomorphy (1) however probably relates to the aquatic habitus of the taxa, as it is also shared by phytosaurs, and node support is low (Fig. 2).

Support for this node in the analysis of Ezcurra et al. (2010) rests on the following unambiguous synapomorphies: (1) absence of fourth trochanter; (2) presence of mid-dorsal neural spines situated at mid-length between the zygapophyses; (3) maximum length of iliac blade less than three times maximum height. Whether *Vancleavea campi* possesses a true fourth trochanter is however controversial (see Nesbitt 2009), and this taxon was thus scored "?" for this taxon in the current analysis. Doswelliids are scored as showing a fourth trochanter in the current analysis as, although not pronounced, a muscle attachment ridge is present in the same location (i.e. at midshaft) and has been considered to be likely to be homologous (Schoch and Sues 2013). The character yielding synapomorphy (2) was excluded from the current analysis as it was deemed to be difficult to score, and that yielding (3) was excluded as it was felt to overlap with character 276 of Nesbitt (2011), but these characters would further support the results presented here. Synapomorphy (2) of Ezcurra et al. 2010 was the same sole synapomorphy found to support this clade in the analysis of Dilkes and Arcucci (2012).

The node in Schoch and Sues (2013) uniting *Vancleavea campi* with dosweliids was supported by four unambiguous synapomorphies: (1) loss of antorbital fossa; (2) regain of prominent pubic tubercle; (3) maximum length of iliac blade less than three times maximum height; (4) midshaft diameter of metatarsal IV less than that of metatarsal III. The character yielding synapomorphy (3) is discussed above. That yielding (1) was scored as inapplicable in the current analysis as *Vancleavea campi* lacks an antorbital fenestra. That yielding (2) was excluded from this analysis due to potential overlap with presence/absence of the pectineal process (character 289) and difficulty in scoring taxa from the literature; furthermore, the erythrosuchid *Garjainia prima* (PIN 951/25) shows a well-developed pubic tubercle, meaning that conclusions drawn regarding the phylogenetic position of erythrosuchids in respect to *Vancleavea campi* based on this character may be doubtful. The character yielding (4) is scored as unknown for *Vancleavea campi* in the current analysis following Nesbitt et al. (2009) and Nesbitt (2011), because identification of metatarsals II-IV is uncertain. The sole unambiguous synapomorphy uniting the large crownward

polytomy including *Doswellia kaltenbachi* and *Vancleavea campi* in the analysis of Parker and Barton (2008) is loss of postaxial intercentra (see discussion above).

The relative position of *Vancleavea campi* in respect to *Erythrosuchus africanus* (the next taxon crownward) found by Desojo et al. (2011) is supported by the following unambiguous synapomorphies: (1) acquisition of antorbital fossa; (2) mid-cervical/mid-dorsal centrum length ratio ≤1; (3) presence of preacetabular process; (4) reduction of prominent pubic tubercle to rugosity; (5) caudoventral process of ischium large (making ischium longer than ilium); (6) presence of fourth trochanter; (7) lateral fossa on dorsal centra below neurocentral suture; (8) anterior process of jugal broad and dorsally expanded anteriorly. The characters yielding (1), (4) and (6) are discussed above. That yielding (2) was excluded from the current analysis as it was deemed difficult to score, somewhat arbitrary in its delimitation, and to be very labile with both primitive and derived states widespread within and outside the crown.

That yielding (5) was excluded as it overlapped with character 298 of Nesbitt (2011; ischium length); character 298 could not, following Nesbitt (2011), be scored for *Vancleavea campi* because no complete ilium and ischium are preserved from the same individual. Regarding (7), presence or absence of a lateral fossa on the dorsal centra was considered to be problematic to score, as several taxa scored as “absent” actually show a rudimentary fossa (e.g. *Mesosuchus browni* - SAM-PK-6046), and the feature varies greatly along the dorsal column (*Mesosuchus browni* - SAM-PK-6046; *Euparkeria capensis* - SAM-PK-6047B). Additionally, the character overlaps with presence/absence of a prezygodiapophyseal lamina, with this character deemed to better represent the variation seen. Regarding (8), slender versus broad anterior process of the jugal was deemed to be too difficult to delimit satisfactorily, and was thus not included in the current analysis.

Supplementary figures

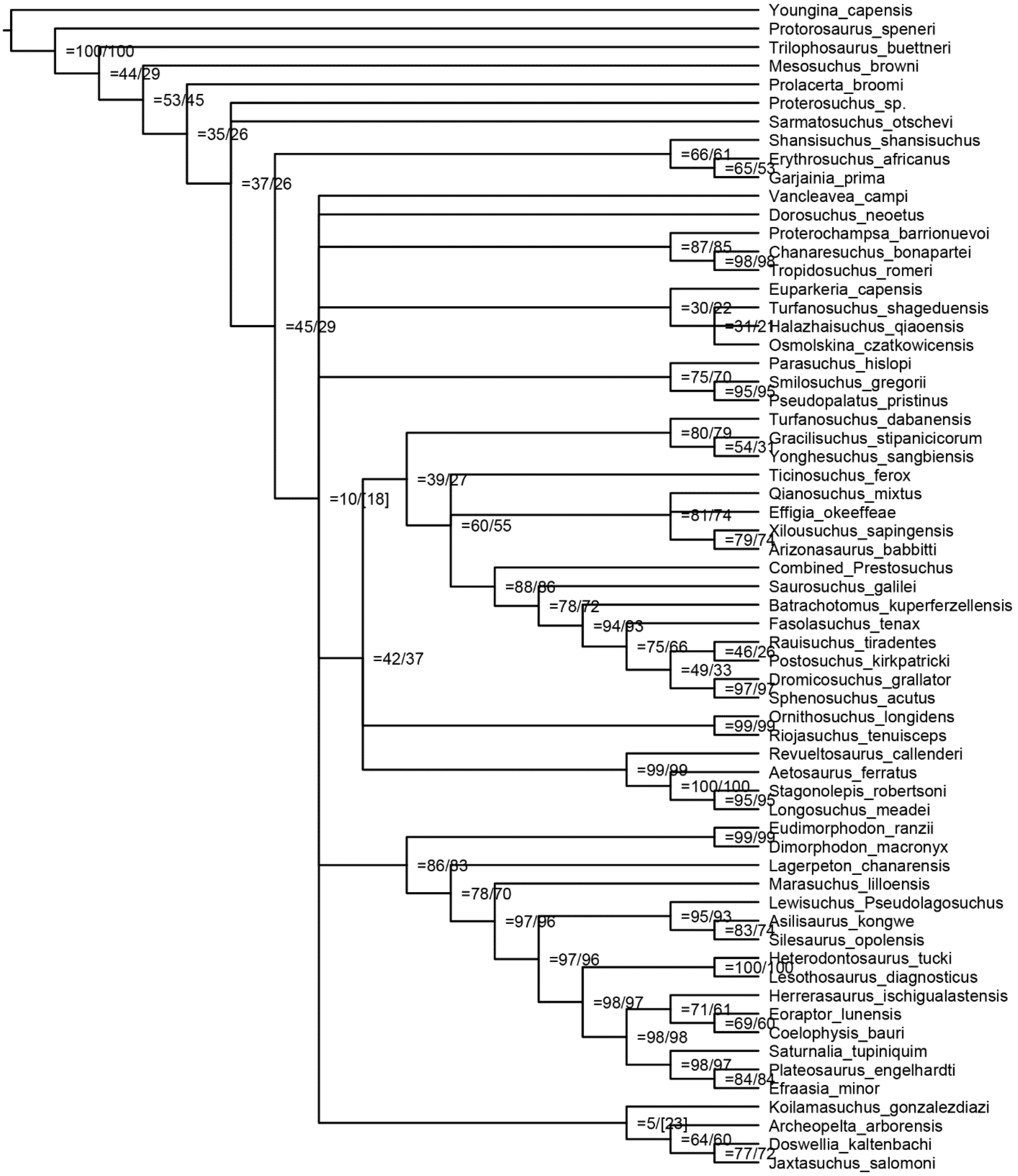

Figure S1. Strict consensus tree of most parsimonious trees found in analysis including character 93. Numbers at nodes are standard (before slash) and GC (after slash) bootstrap values.

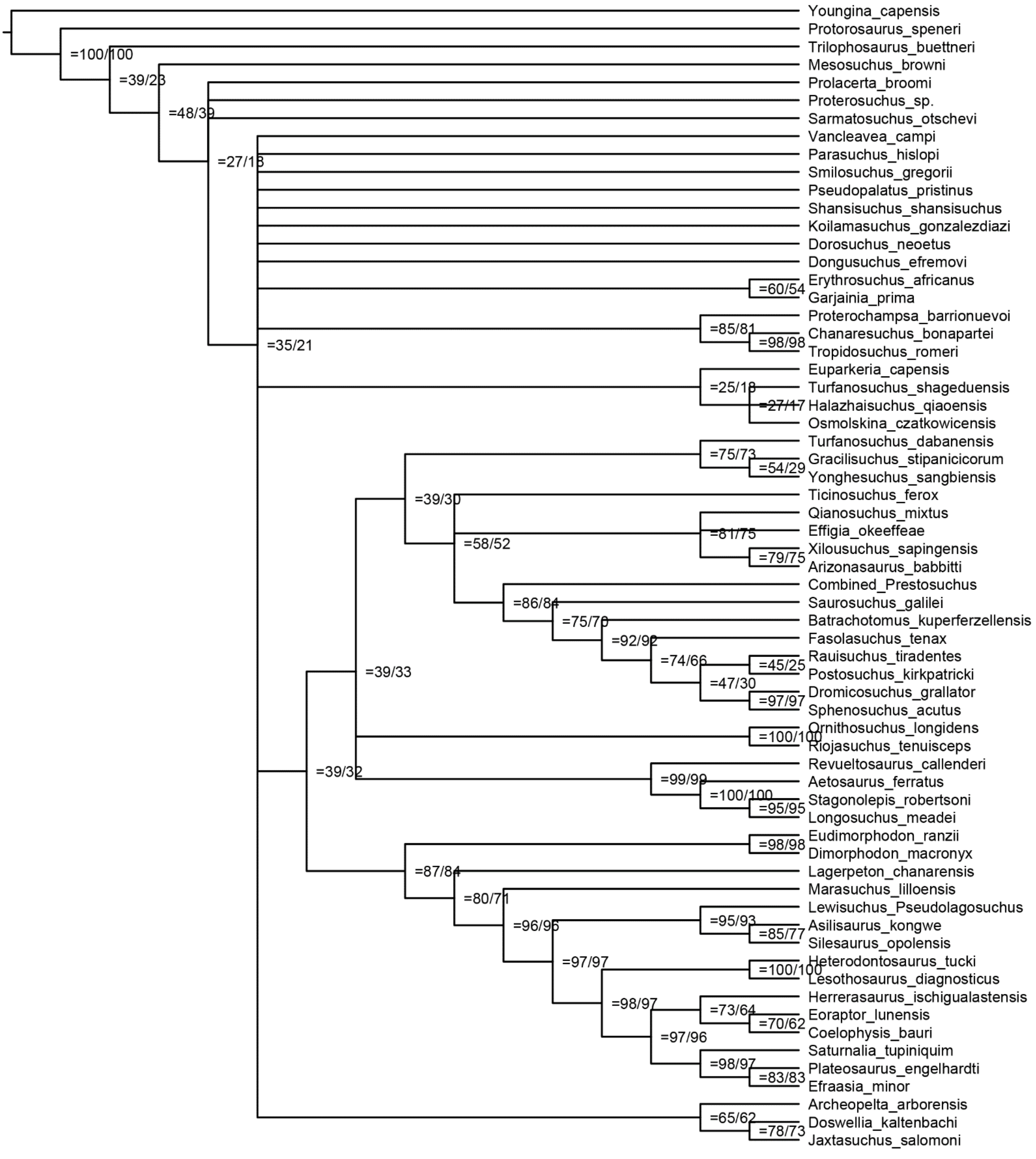

Figure S2. Strict consensus tree of most parsimonious trees found in analysis including *Dongusuchus efremovi* (and excluding character 93). Numbers at nodes are standard (before slash) and GC (after slash) bootstrap values.

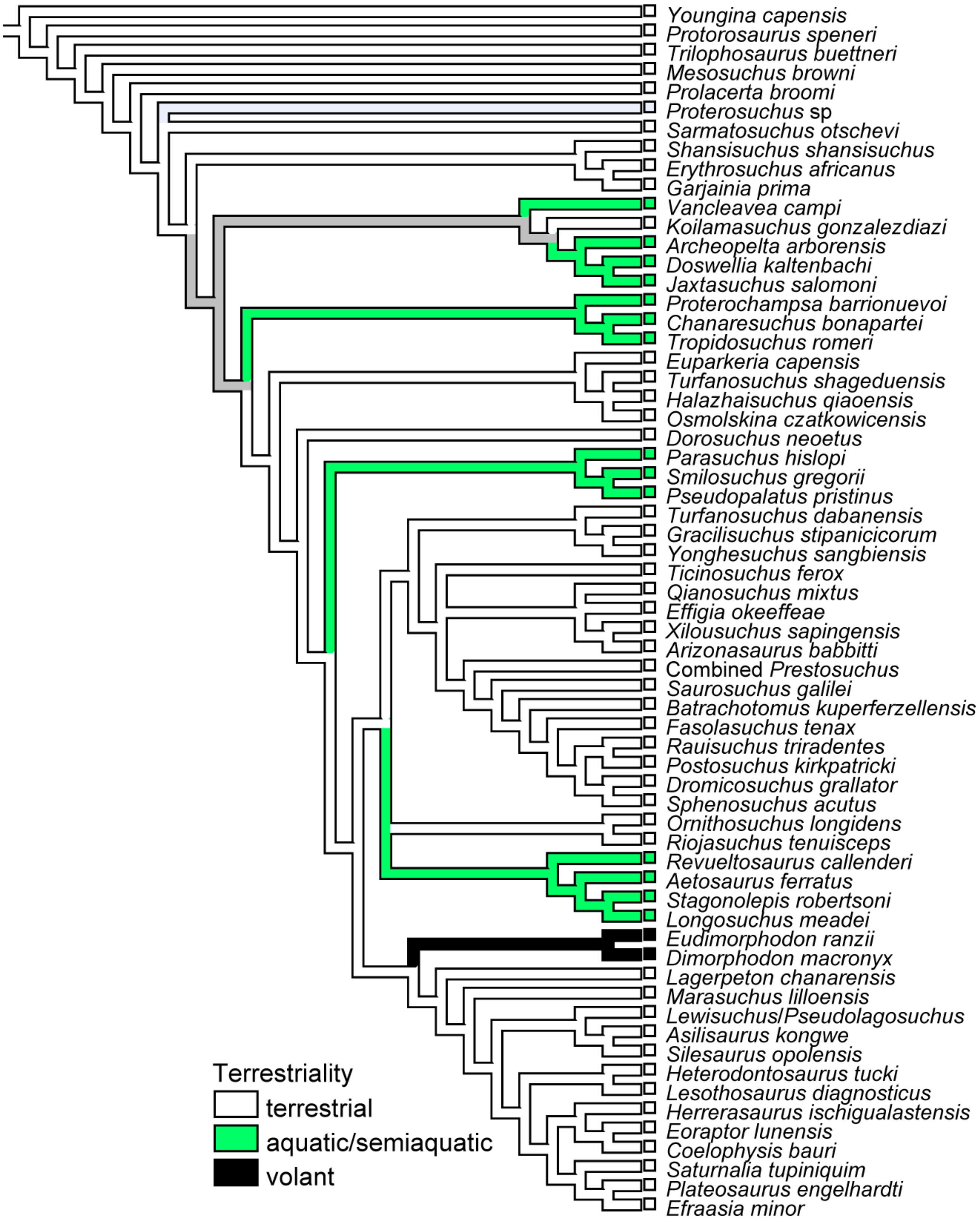

Figure S3. Strict consensus tree from the analysis presented in Chapter 6, showing optimization of degree of terrestriality using squared change parsimony.

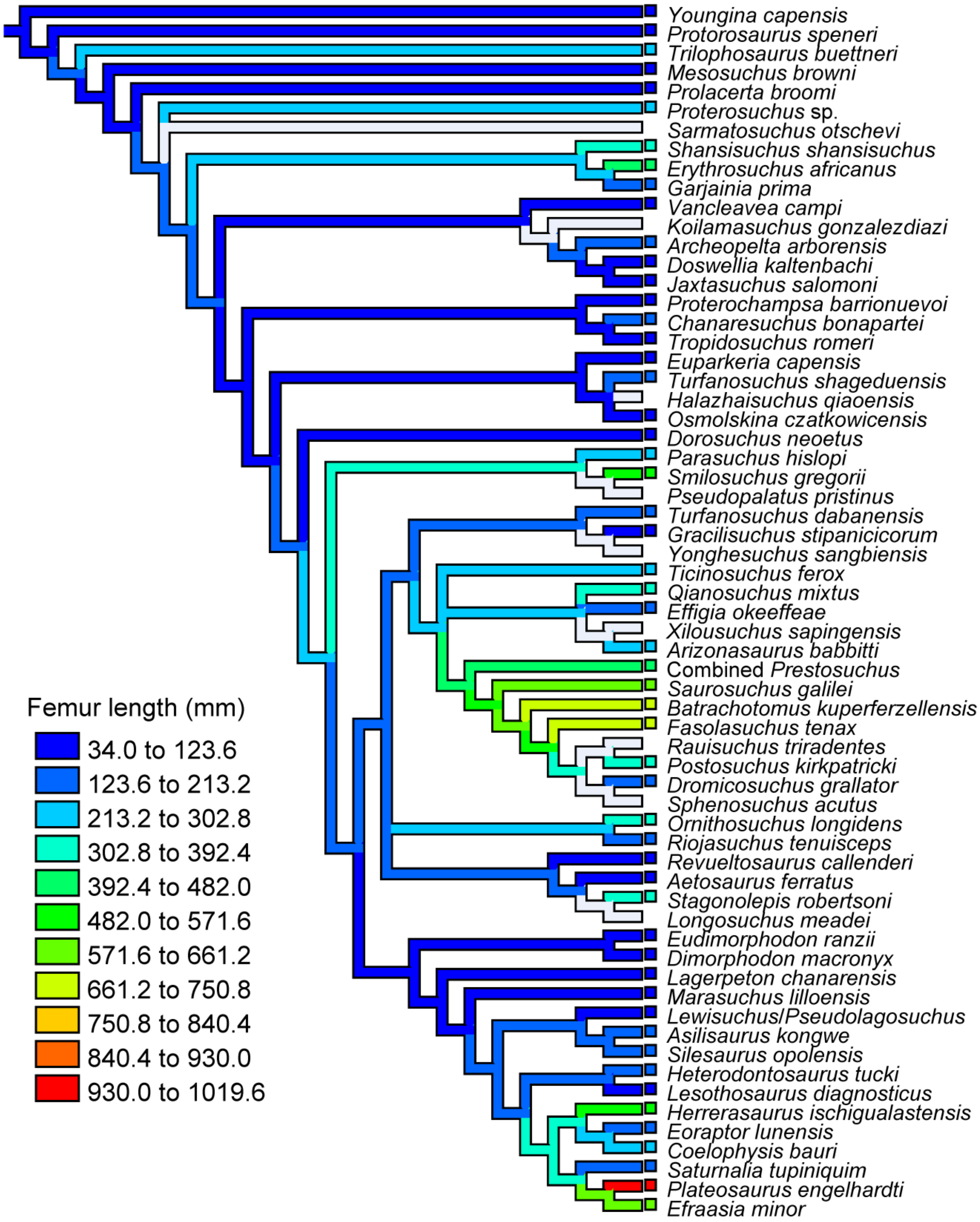

Figure S4. Strict consensus tree from the analysis presented in Chapter 6, showing optimization of femoral length using squared change parsimony.

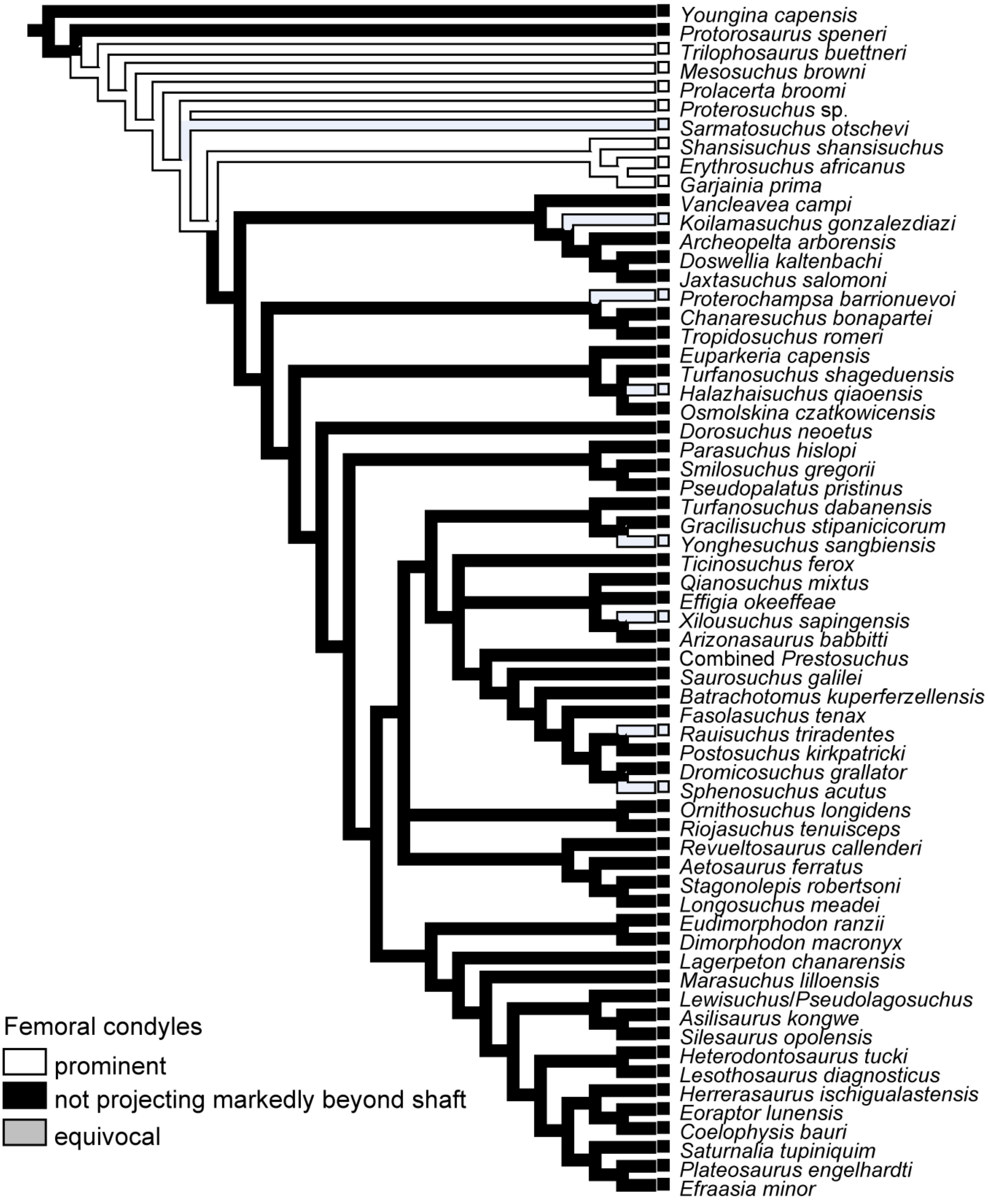

Figure S5. Strict consensus tree from the analysis presented in Chapter 6, showing optimization of degree of projection of femoral condyles (character 313 in the phylogenetic matrix) using squared change parsimony.

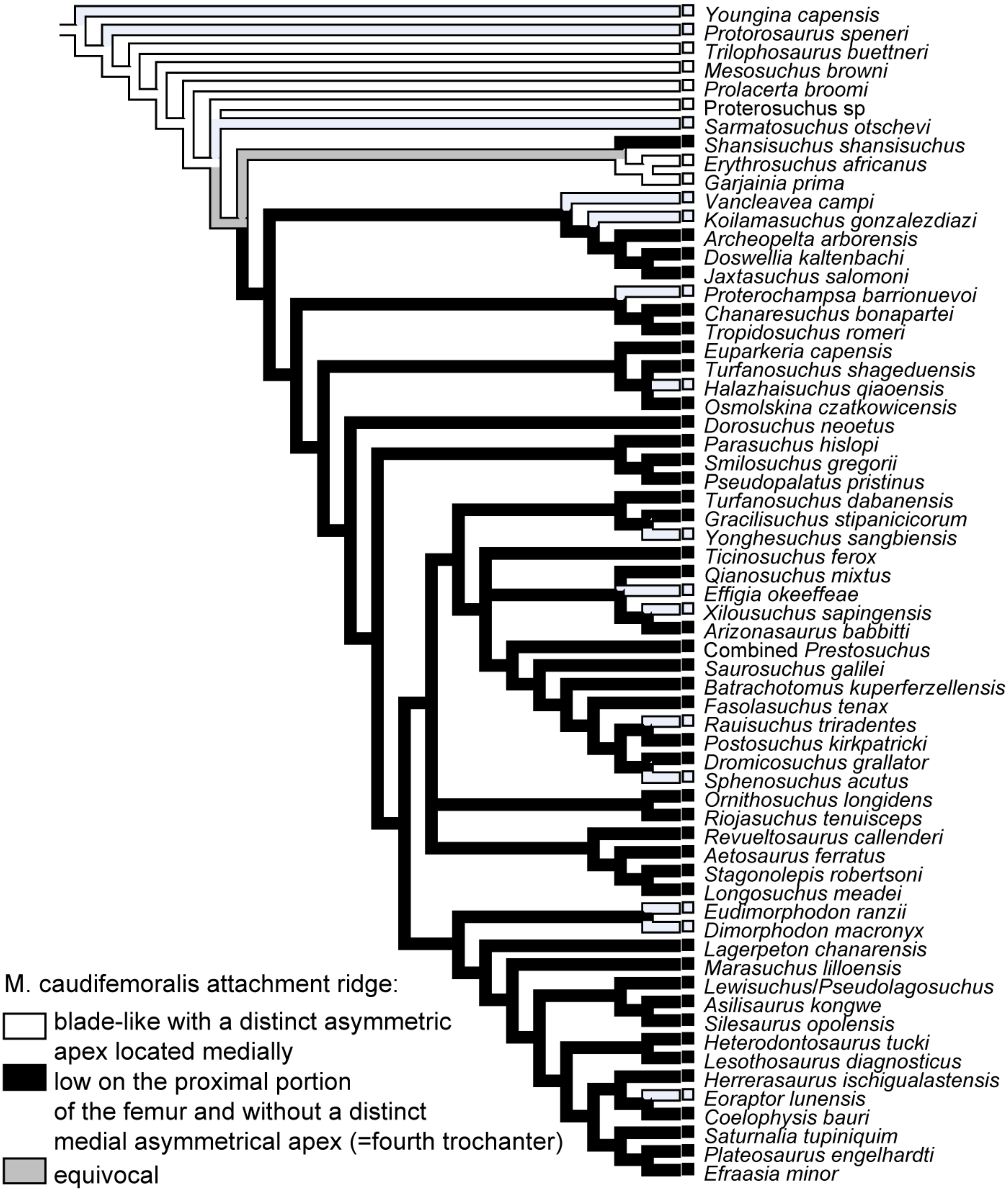

Figure S6. Strict consensus tree from the analysis presented in Chapter 6, showing optimization of form of m. caudifemoralis attachment ridge (character 310 in the phylogenetic matrix) using squared change parsimony.

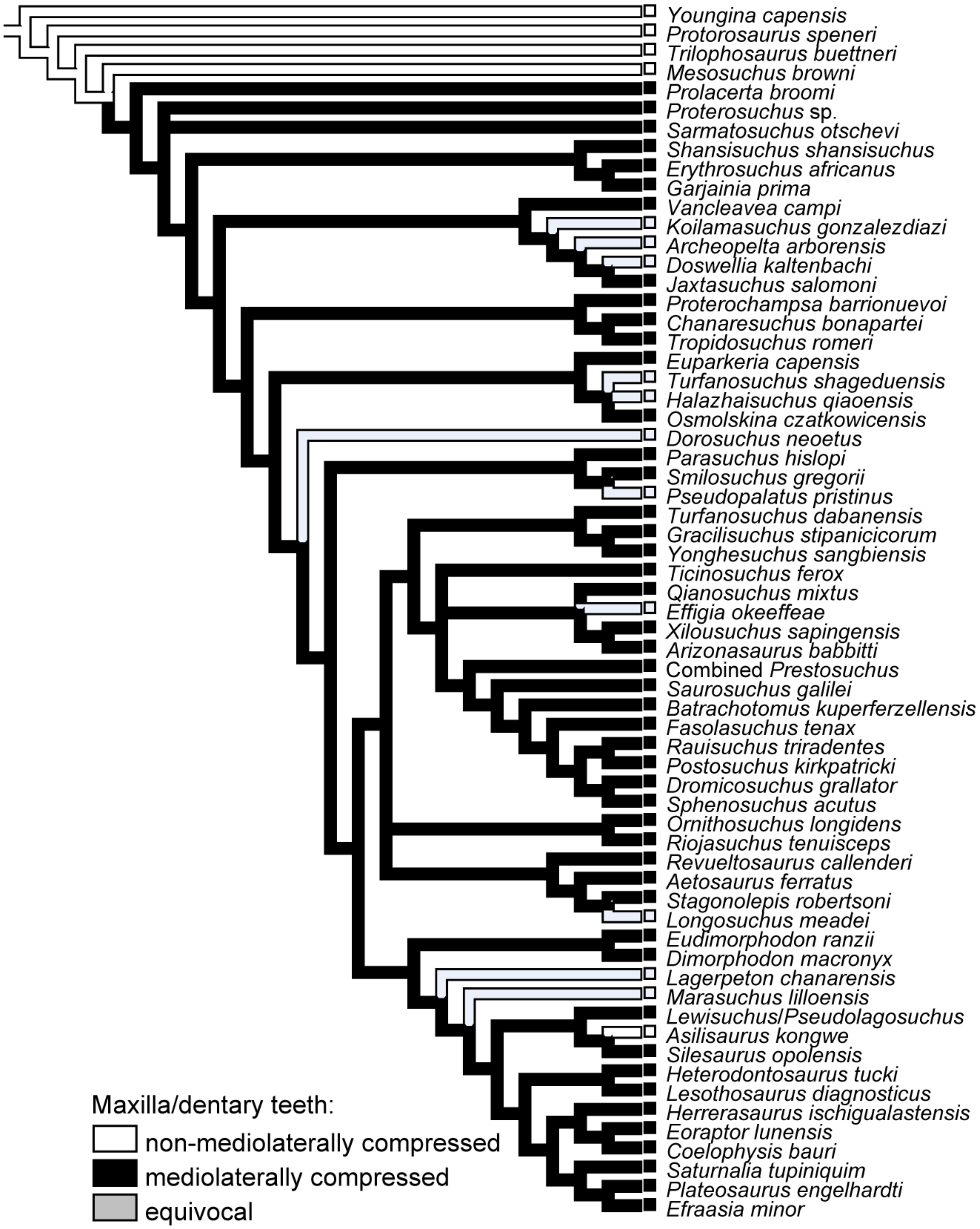

Figure S7. Strict consensus tree from the analysis presented in Chapter 6, showing optimization of shape of maxilla/dentary teeth (character 165 in the phylogenetic matrix) using squared change parsimony.

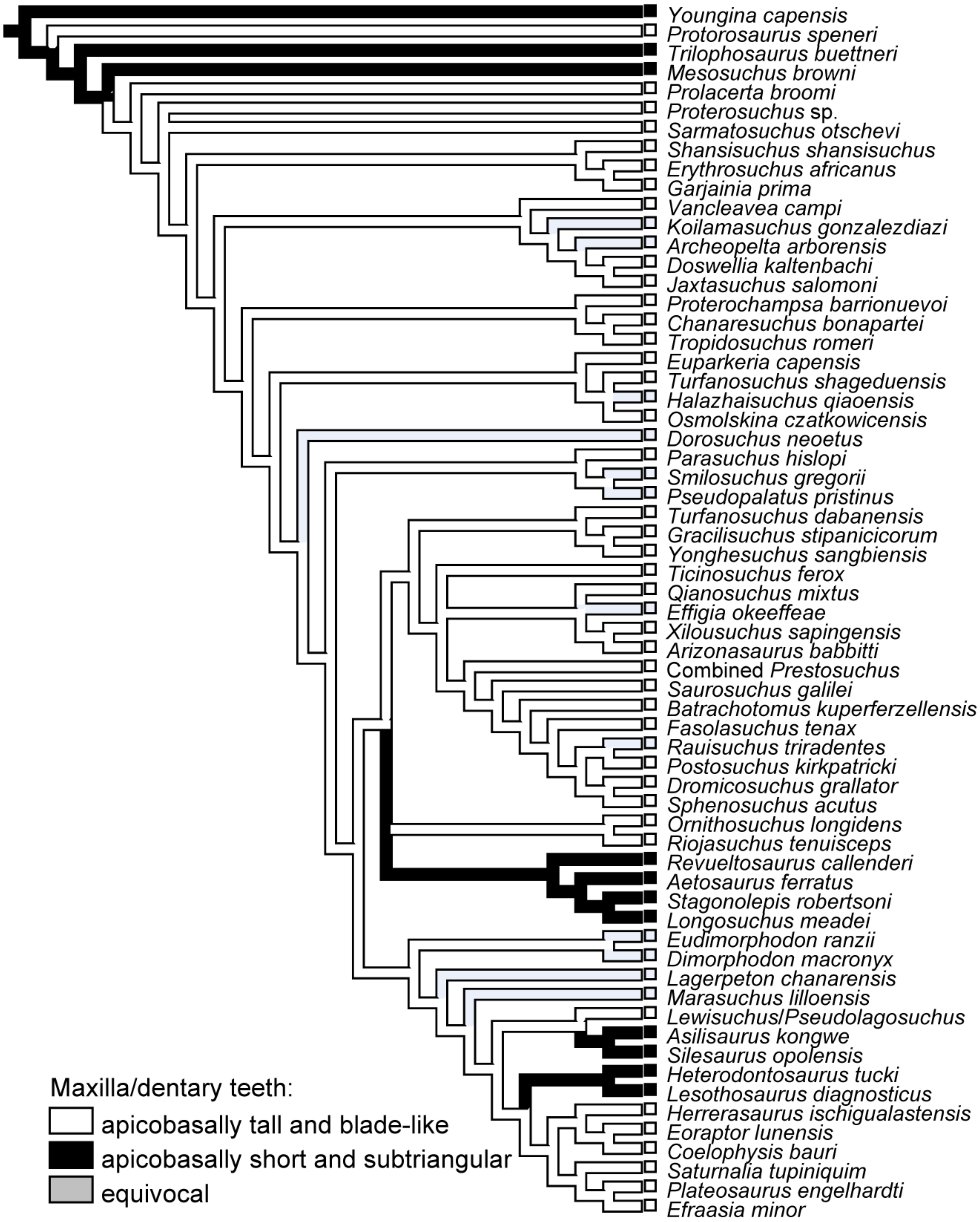

Figure S8. Strict consensus tree from the analysis presented in Chapter 6, showing optimization of height and shape of maxilla/dentary teeth (character 164 in the phylogenetic matrix) using squared change parsimony.

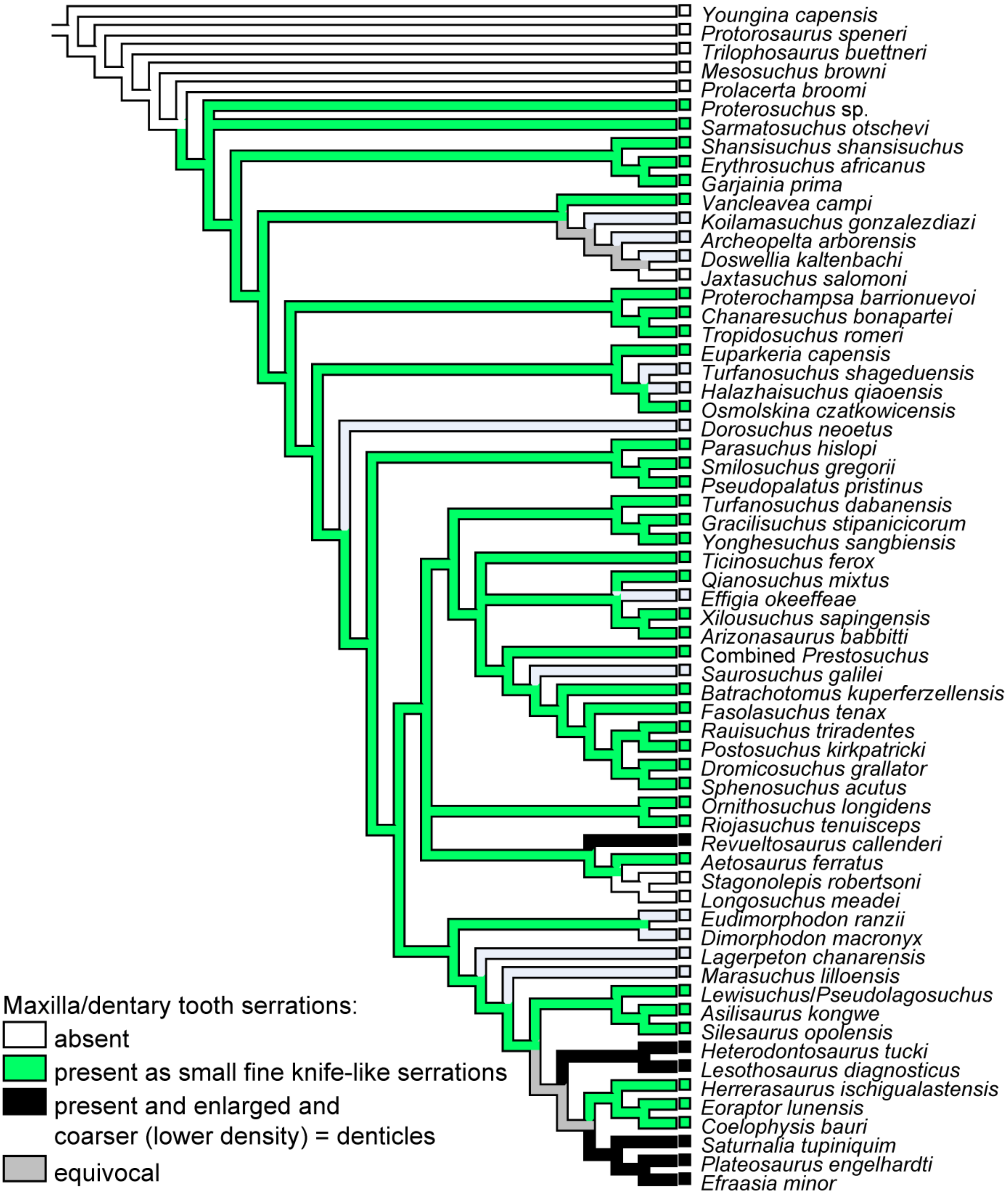

Figure S9. Strict consensus tree from the analysis presented in Chapter 6, showing optimization of serrations on maxilla/dentary teeth (character 160 in the phylogenetic matrix) using squared change parsimony.

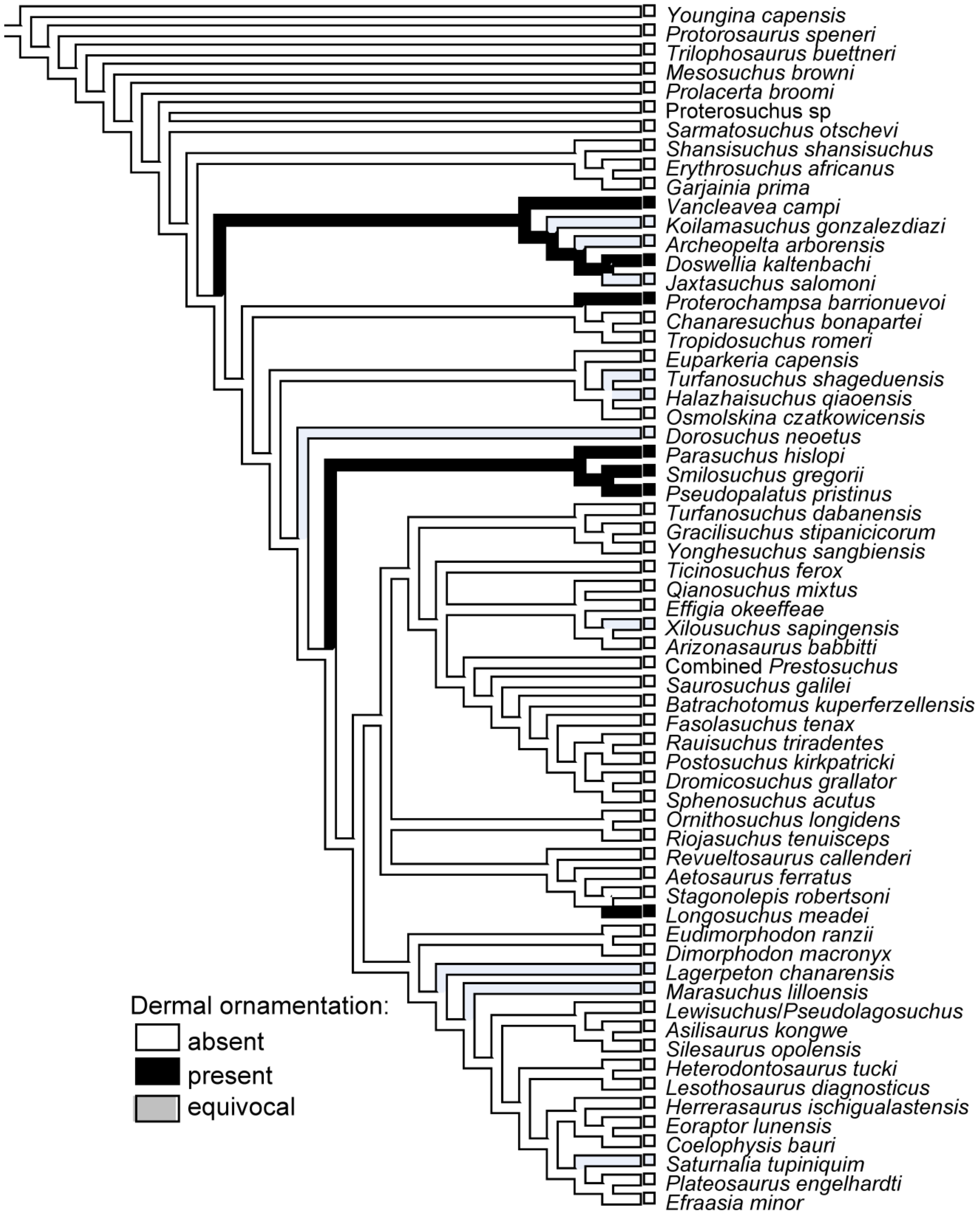

Figure S10. Strict consensus tree from the analysis presented in Chapter 6, showing optimization of presence of dermal ornamentation (character 33 in the phylogenetic matrix) using squared change parsimony.

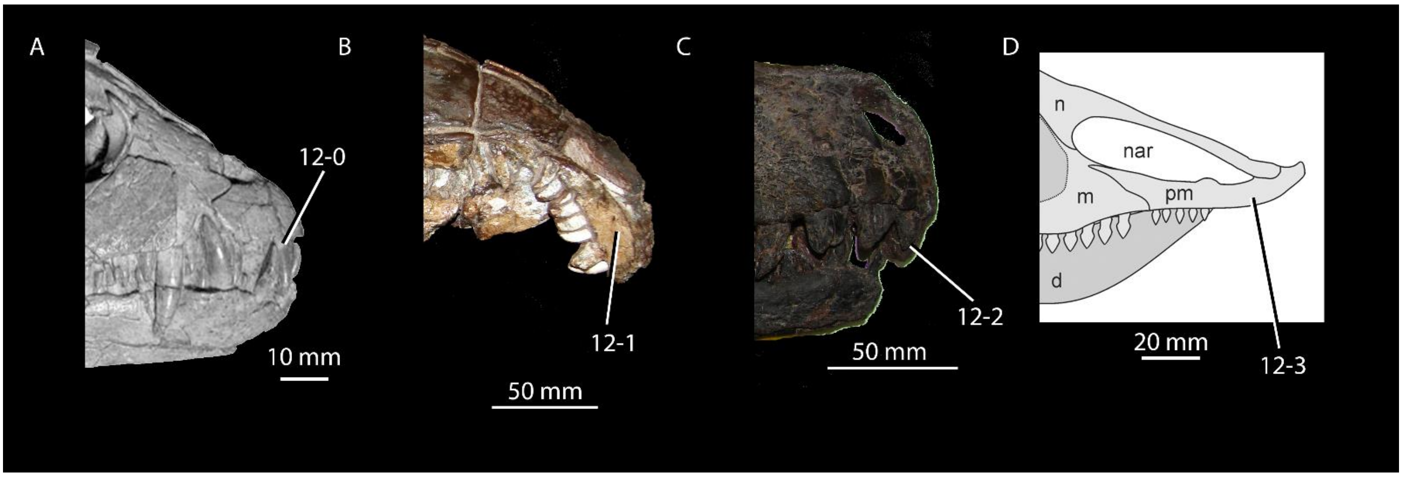

Figure S11. Examples of character states for character 12 – procumbent premaxilla. (A) articulated right premaxilla of *Vancleavea campi* GR 138 in lateral view (from Nesbitt et al. 2009); (B) articulated right premaxilla of *Proterosuchus fergusi* SAM-PK-11208 in lateral view; (C) articulated right premaxilla of *Herrerasaurus ischigualastensis* PVSJ 407 in lateral view; (D) reconstruction of right premaxilla in articulation of *Stagonolepis robertsoni* in lateral view (from Schoch 2007). Photographs B and C courtesy of M. D. Ezcurra. 12-0, 12-1, 12-2 and 12-3 indicate character states 0-3 of character 12.

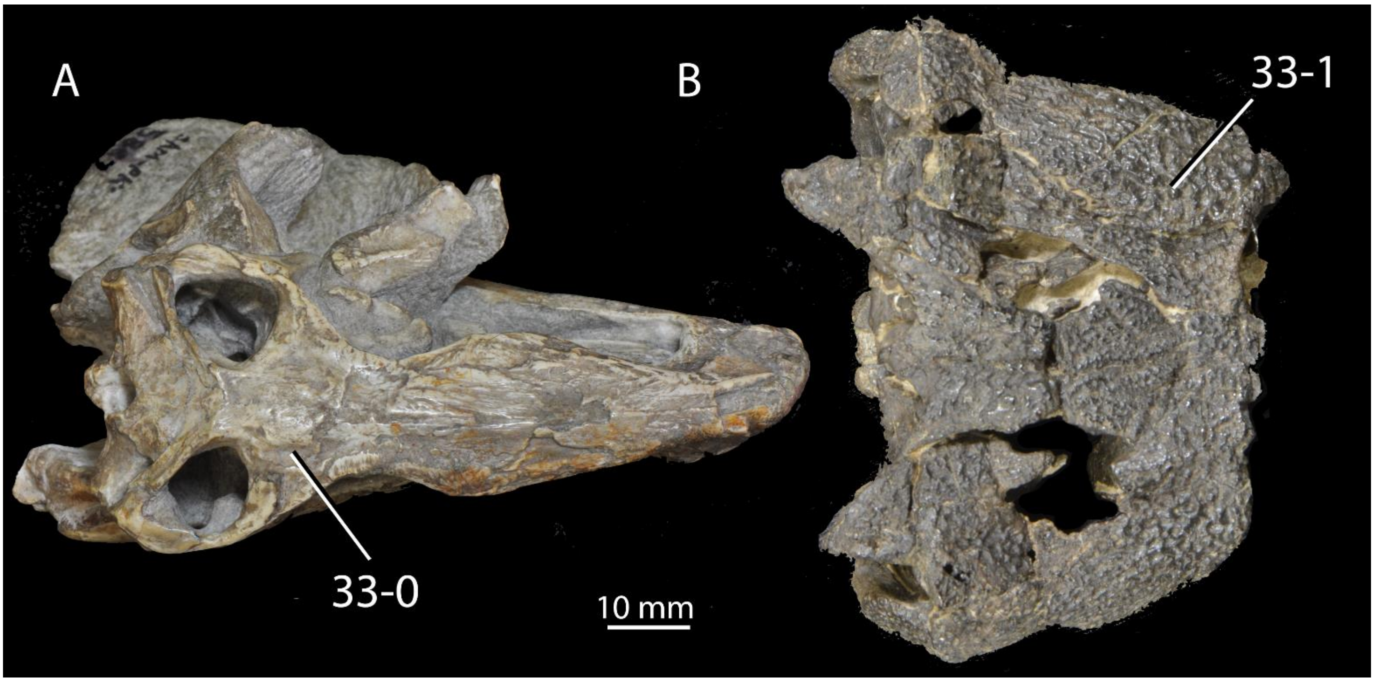

Figure S12. Examples of character states for character 33 – presence of pit and ridge ornamentation on dermal bones of skull. (A) *Euparkeria capensis* SAM-PK-5867 in dorsal view; (B) *Doswellia kaltenbachi* USNM 437574 in dorsal view. 33-0 and 33-1 indicate character state 0 and 1 respectively of character 33.

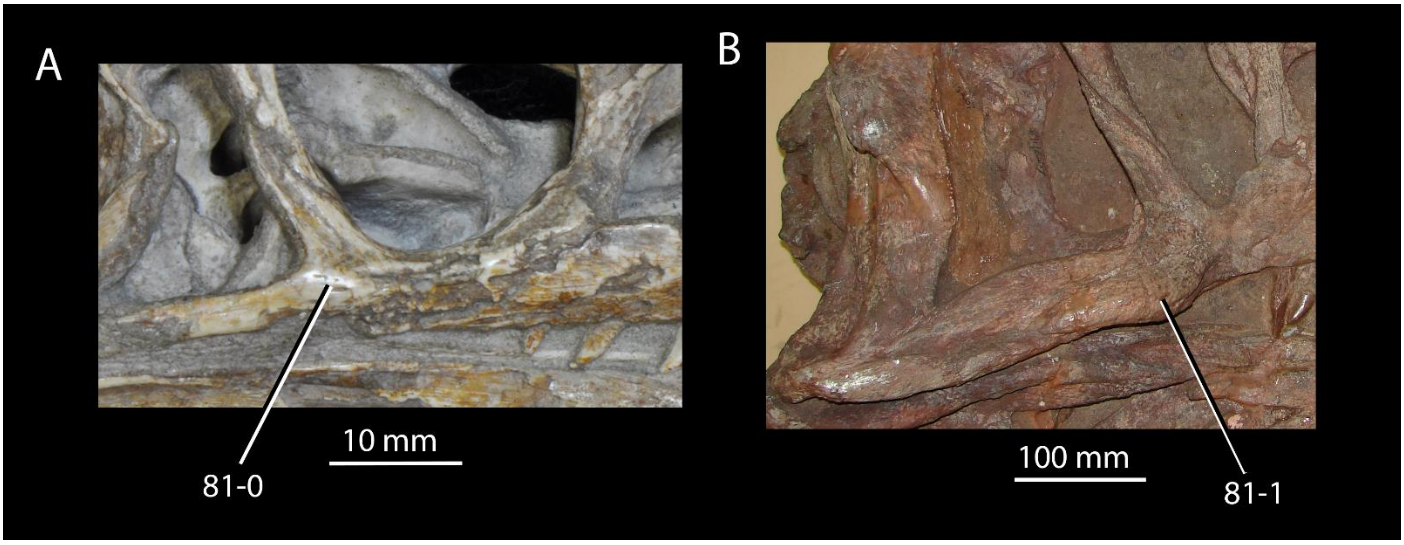

Figure S13. Examples of character states for character 81 – presence of ventral expansion of jugal at base of postorbital bar. (A) *Euparkeria capensis* SAM-PK-5867 in right lateral view; (B) *Erythrosuchus africanus* BPI/1/5207 in right lateral view. 81-0 and 81-1 indicate character state 0 and 1 respectively of character 81.

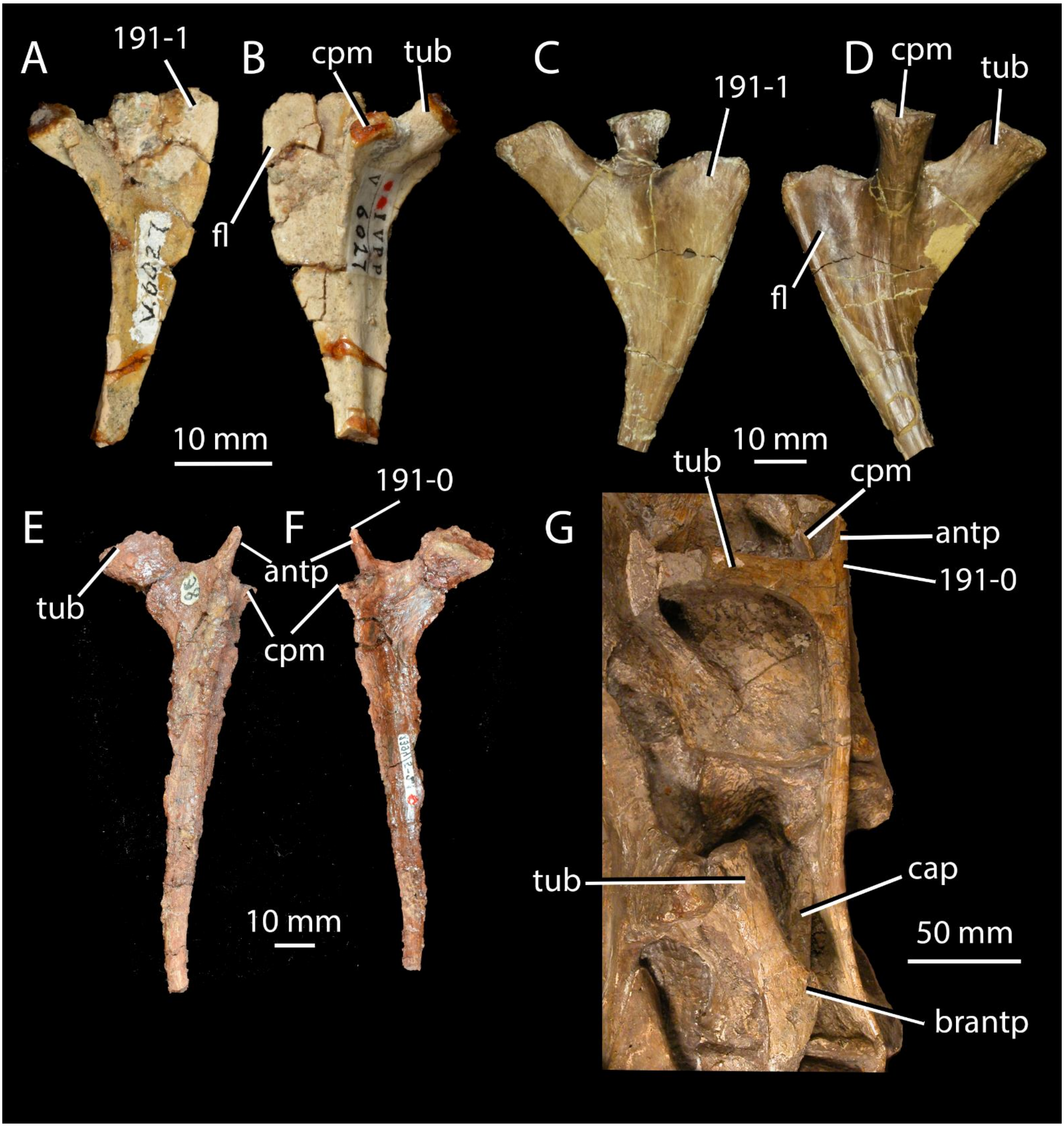

Figure S14. Examples of character states for character 191 – flange on cervical ribs. Right cervical rib of *Halazhaisuchus qiaoensis* IVPP V6027-9 in dorsal (A) and ventral (B) views; left cervical rib (image mirrored for comparison) of *Batrachotomus kupferzellensis* SMNS 91046 in dorsal (C) and ventral (D) views; left cervical rib (image mirrored for comparison) of *Garjainia prima* PIN 2394/5 in dorsal (E) and ventral (F) views; (G) right cervical ribs of *Plateosaurus engelhardti* SMNS 13200 in dorsal view. ap, anterior process of rib; bap, broken anterior process of rib; cap, capitulum; fl, flange; tub, tuberculum. 191-0 and 191-1 indicate character state 0 and 1 respectively of character 191.

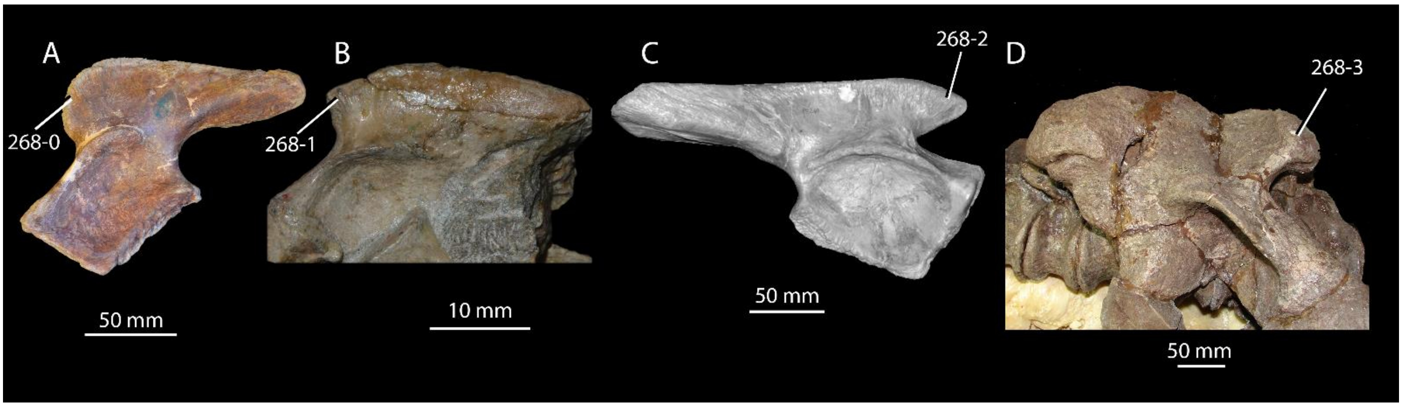

Figure S15. Examples of character states for character 268 – shape of preacetabular process. (A) left ilium of *Garjainia prima* PIN 951/8 in lateral view; (B) left ilium of *Euparkeria capensis* SAM-PK-6049 in lateral view; (C) right ilium of *Batrachotomus kupferzellensis* SMNS 80269 in lateral view; (D) right ilium of *Herrerasaurus ischigualastensis* in lateral view. 268-0,-1,-2 and -3 indicate character states 0-3 of character 268.

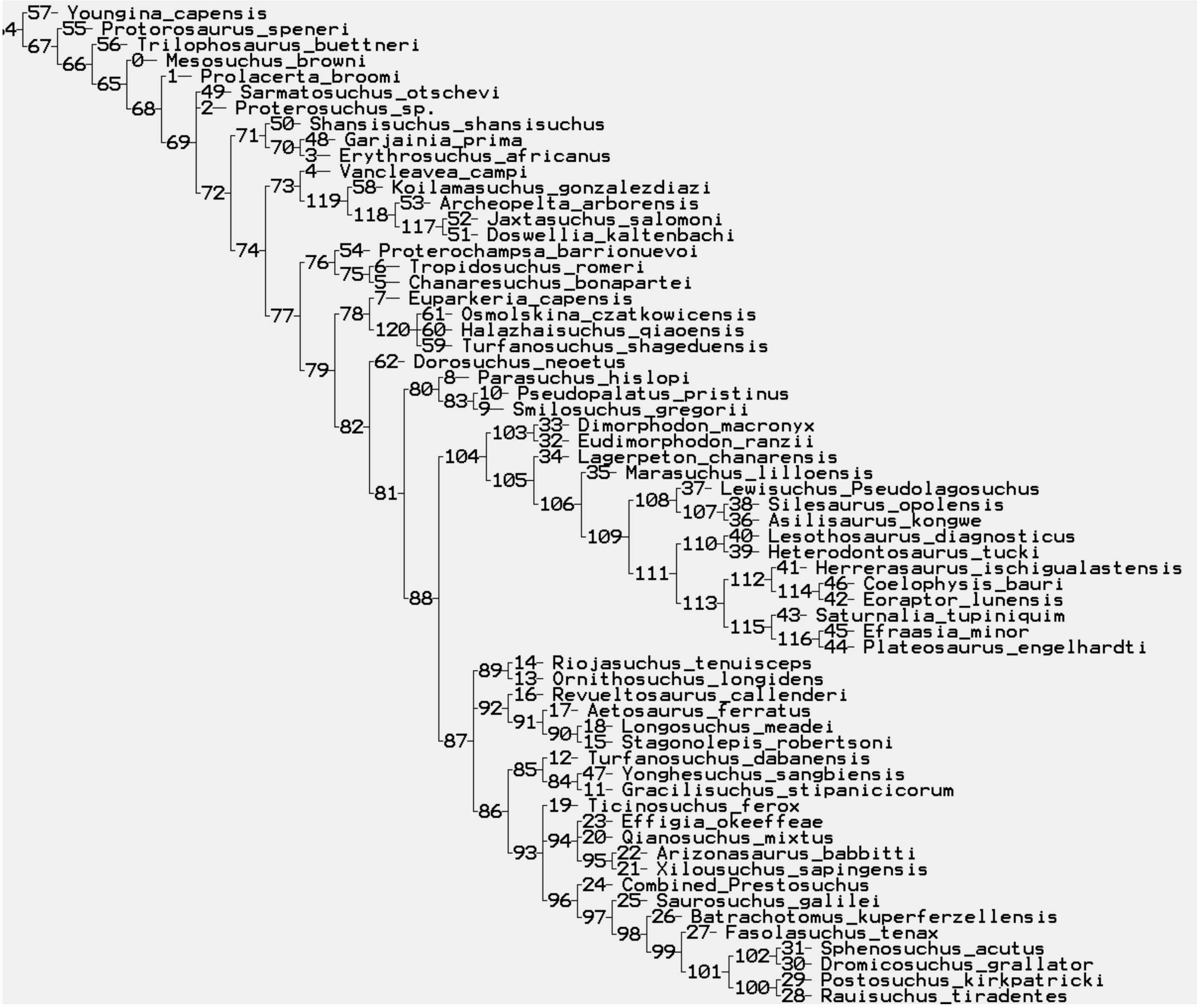

Figure S16. Strict consensus tree showing node numbers to which those in the synapomorphy list refer.

Supplementary references

Barberena, M.C. 1978. A huge thecodont skull from the Triassic of Brazil. Pesquisas 9: 62–75.

Bonaparte, J.F. 1971. Los tetrápodos del sector superior de la Formación Los Colorados, La Rioja, Argentina (Triásico Superior). Primera Parte. Opera Lilloana 22: 1–183.

Camp, C.L. 1945a. *Prolacerta* and the protorosaurian reptiles. Part I. American Journal of Science 243: 17–32.

Colbert, E.H. 1989. The Triassic dinosaur *Coelophysis*. Museum of Northern Arizona Bulletin 57: 1–174.

Chatterjee, S. 1978. A primitive parasuchid (phytosaur) reptile from the Upper Triassic Maleri Formation of India. Palaeontology 21: 83-127.

Colbert, E.H. 1947. Studies of the phytosaurs *Machaeroprosopus* and *Rutiodon*. Bulletin of the American Museum of Natural History 88: 53-96.

Cruickshank ARI. 1972b. The proterosuchian thecodonts. In: Joysey, K.A., Kemp, T.S. (Eds.) Studies in Vertebrate Evolution. Oliver and Boyd, Edinburgh, pp. 89–119.

Cruickshank, A.R.I. 1979. The ankle joint in some early archosaurs. South African Journal of Science 75: 168–178.

Gottmann-Quesada, A., Sander, P.M. 2009. A redescription of the early archosauromorph *Protorosaurus speneri* Meyer, 1832, and its phylogenetic relationships. Paläontolographica, Abteilung A, Paläontologie und Stratigraphie 287: 4-6.

Langer, M.C., Benton, M.J. 2006. Early dinosaurs: a phylogenetic study. Journal of Systematic Palaeontology 4: 309–358.

Mehl, M.G. 1928. *Pseudopalatus pristinus* a new genus and species of phytosaurs from Arizona. The University of Missouri Studies 3: 7-22.

Novas, F.E. 1993. New information on the systematics and postcranial skeleton of *Herrerasaurus ischigualastensis* (Theropoda: Herrerasauridae) from the Ischigualasto Formation (Upper Triassic) of Argentina. Journal of Vertebrate Paleontology 13: 400-423.

Ochev, V.G. 1981. Ob *Erythrosuchus* (*Garjainia*) *primus* (Ochev). Voprosy Geologii YuzhnogoUrala I Povolzh'ya 22: 3-22.

Ochev, V.G. 1958. Novye dannye po psevdozukhiyam SSSR. Dokaldy Akademii Nauk SSSR 123: 749-751.

Padian, K. 1983. Osteology and functional morphology of *Dimorphodon macronyx* (Buckland) (Pterosauria: Rhamphorhynchoidea) based on new material in the Yale Peabody Museum. Postilla 189: 1-44.

Romer, A.S. 1972b. The Chañares (Argentina) Triassic reptile fauna. XII. The postcranial skeleton of the thecodont *Chanaresuchus*. Breviora 385: 1–21.

Sill, W.D. 1967. *Proterochampsa barrionuevoi* and the early evolution of the Crocodilia. Bulletin of the Museum of Comparative Zoology 135: 415-446.

Spielmann, J.A., Lucass, S.G., Rinehart, L.F., Heckert, A.B. 2008. The Late Triassic archosauromorph *Trilophosaurus*. New Mexico Museum of Natural History and Science Bulletin 43: 1-177.

Walker, A D. 1961. Triassic reptiles from the Elgin area: *Stagonolepis*, *Dasygnathus*, and their allies. Philosophical Transactions of the Royal Society of London B 244: 103-204.

Yates, A.M. 2003. The species taxonomy of the sauropodomorph dinosaurs from the Lowenstein Formation (Norian, Late Triassic) of Germany. Palaeontology 46: 317–337.

Synapomorphy list

Unambiguous synapomorphies optimizing at all nodes and autapomorphies of all taxa are listed below. Node numbers refer to those in Fig. S16 and taxa are listed as given in the same figure.

Mesosuchus_browni :

All trees:

Char. 1: 0 --> 1

Char. 38: 0 --> 1

Char. 61: 0 --> 1

Char. 160: 0 --> 1

Char. 178: 0 --> 1

Prolacerta_broomi :

All trees:

Char. 120: 1 --> 0

Char. 184: 0 --> 1

Some trees:

Char. 168: 1 --> 0

Proterosuchus_sp. :

All trees:

Char. 80: 0 --> 1

Some trees:

Char. 76: 0 --> 3

Char. 103: 0 --> 1

Char. 133: 0 --> 1

Char. 168: 1 --> 0

Char. 335: 1 --> 0

Erythrosuchus_africanus :

All trees:

Char. 77: 0 --> 1

Char. 189: 0 --> 1

Some trees:

Char. 7: 1 --> 0

Vancleavea_campi :

All trees:

Char. 16: 0 --> 1

Char. 158: 0 --> 1

Char. 178: 0 --> 1

Char. 265: 1 --> 0

Char. 401: 0 --> 1

Char. 402: 0 --> 1

Char. 404: 0 --> 2

Chanaresuchus_bonapartei :

All trees:

Char. 15: 0 --> 1

Char. 308: 0 --> 1

Char. 402: 0 --> 1

Tropidosuchus_romeri :

All trees:

Char. 41: 0 --> 1

Char. 174: 0 --> 1

Char. 353: 0 --> 1

Euparkeria_capensis :

All trees:

Char. 113: 1 --> 0

Some trees:

Char. 331: 1 --> 0

Parasuchus_hislopi :

All trees:

Char. 217: 1 --> 0

Char. 219: 0 --> 1

Smilosuchus_gregorii :

All trees:

Char. 114: 1 --> 0

Pseudopalatus_pristinus :

All trees:

Char. 77: 0 --> 1

Gracilisuchus_stipanicicorum :

All trees:

Char. 5: 2 --> 1

Char. 25: 0 --> 1

Char. 30: 2 --> 1

Char. 76: 0 --> 1

Turfanosuchus_dabanensis :

All trees:

Char. 45: 0 --> 1

Char. 57: 0 --> 2

Char. 58: 0 --> 1

Char. 97: 2 --> 0

Char. 114: 1 --> 0

Char. 192: 0 --> 1

Char. 353: 0 --> 1

Char. 356: 1 --> 0

Ornithosuchus_longidens :

All trees:

Char. 308: 0 --> 1

Char. 387: 0 --> 1

Some trees:

Char. 399: 2 --> 1

Riojasuchus_tenuisceps :

All trees:

Char. 27: 0 --> 1

Char. 52: 0 --> 1

Char. 185: 0 --> 1

Char. 205: 0 --> 1

Char. 224: 0 --> 1

Stagonolepis_robertsoni :

All trees:

Char. 24: 0 --> 1

Char. 93: 0 --> 1

Char. 251: 0 --> 1

Revueltosaurus_callenderi :

All trees:

Char. 3: 0 --> 3

Char. 13: 0 --> 3

Char. 21: 0 --> 1

Char. 24: 0 --> 1

Char. 25: 0 --> 2

Char. 46: 0 --> 1

Char. 158: 0 --> 1

Char. 159: 1 --> 2

Char. 182: 1 --> 2

Aetosaurus_ferratus :

All trees:

Char. 5: 2 --> 1

Char. 37: 0 --> 1

Char. 83: 0 --> 1

Char. 113: 1 --> 0

Longosuchus_meadei :

All trees:

Char. 4: 0 --> 1

Char. 32: 0 --> 1

Ticinosuchus_ferox :

Some trees:

Char. 280: 1 --> 0

Qianosuchus_mixtus :

All trees:

Char. 5: 2 --> 3

Char. 15: 0 --> 1

Char. 30: 0 --> 1

Char. 190: 1 --> 0

Some trees:

Char. 11: 0 --> 2

Char. 25: 0 --> 1

Char. 280: 1 --> 0

Char. 284: 2 --> 1

Char. 291: 1 --> 0

Char. 333: 1 --> 0

Xilousuchus_sapingensis :

All trees:

Char. 103: 0 --> 1

Char. 115: 1 --> 0

Char. 178: 0 --> 1

Char. 194: 1 --> 0

Arizonasaurus_babbitti :

All trees:

No autapomorphies:

Effigia_okeeffeae :

All trees:

Char. 5: 2 --> 4

Char. 24: 0 --> 1

Char. 29: 1 --> 0

Char. 46: 0 --> 1

Char. 47: 0 --> 1

Char. 53: 1 --> 0

Char. 60: 0 --> 1

Char. 62: 0 --> 2

Char. 79: 1 --> 0

Char. 84: 0 --> 1

Char. 95: 1 --> 0

Char. 102: 0 --> 1

Char. 112: 0 --> 1

Char. 147: 0 --> 1

Char. 148: 0 --> 1

Char. 149: 1 --> 0

Char. 155: 0 --> 1

Char. 157: 0 --> 2

Char. 177: 0 --> 1

Char. 180: 0 --> 1

Char. 181: 0 --> 1

Char. 192: 0 --> 1

Char. 193: 0 --> 1

Char. 202: 0 --> 1

Char. 205: 0 --> 1

Char. 217: 1 --> 0

Char. 225: 1 --> 0

Char. 227: 0 --> 1

Char. 263: 0 --> 1

Char. 266: 0 --> 2

Char. 272: 0 --> 1

Char. 275: 0 --> 1

Char. 289: 1 --> 2

Char. 296: 1 --> 3

Char. 298: 0 --> 1

Char. 307: 0 --> 1

Char. 316: 0 --> 1

Char. 389: 2 --> 1

Some trees:

Char. 71: 0 --> 1

Char. 208: 1 --> 0

Char. 209: 0 --> 1

Char. 210: 2 --> 0

Char. 286: 0 --> 2

Char. 292: 1 --> 0

Char. 323: 0 --> 1

Char. 366: 2 --> 1

Char. 380: 0 --> 1

Char. 381: 0 --> 1

Combined_Prestosuchus :

All trees:

Char. 26: 0 --> 1

Char. 31: 0 --> 1

Char. 48: 0 --> 1

Char. 114: 1 --> 0

Char. 120: 1 --> 0

Char. 210: 2 --> 1

Char. 286: 0 --> 1

Char. 343: 1 --> 0

Saurosuchus_galilei :

All trees:

Char. 47: 0 --> 1

Char. 141: 0 --> 1

Batrachotomus_kuperferzellensis :

All trees:

Char. 8: 0 --> 1

Char. 17: 0 --> 1

Char. 22: 0 --> 1

Char. 23: 0 --> 1

Char. 25: 1 --> 0

Char. 35: 0 --> 1

Char. 200: 0 --> 1

Char. 204: 0 --> 1

Char. 240: 0 --> 1

Char. 291: 1 --> 0

Fasolasuchus_tenax :

All trees:

No autapomorphies:

Rauisuchus_tiradentes :

All trees:

Char. 80: 0 --> 1

Char. 400: 0 --> 1

Postosuchus_kirkpatricki :

All trees:

Char. 185: 0 --> 1

Dromicosuchus_grallator :

All trees:

Char. 1: 1 --> 0

Char. 11: 0 --> 2

Sphenosuchus_acutus :

All trees:

Char. 61: 0 --> 1

Char. 62: 1 --> 2

Char. 64: 0 --> 1

Char. 68: 1 --> 0

Eudimorphodon_ranzii :

All trees:

Char. 5: 2 --> 1

Char. 44: 0 --> 1

Char. 84: 0 --> 1

Char. 130: 1 --> 0

Char. 183: 0 --> 1

Char. 380: 0 --> 1

Dimorphodon_macronyx :

All trees:

Char. 134: 0 --> 2

Lagerpeton_chanarensis :

All trees:

Char. 298: 0 --> 1

Char. 310: 0 --> 1

Char. 319: 0 --> 1

Char. 383: 1 --> 0

Some trees:

Char. 368: 0 --> 2

Marasuchus_lilloensis :

All trees:

Char. 267: 2 --> 3

Asilisaurus_kongwe :

All trees:

Char. 164: 1 --> 0

Char. 292: 0 --> 1

Lewisuchus_Pseudolagosuchus :

All trees:

Char. 71: 0 --> 1

Char. 167: 1 --> 0

Char. 194: 1 --> 0

Char. 390: 0 --> 1

Silesaurus_opolensis :

All trees:

Char. 145: 0 --> 1

Char. 204: 0 --> 1

Char. 269: 0 --> 1

Char. 297: 0 --> 2

Char. 307: 1 --> 0

Char. 322: 0 --> 1

Char. 323: 0 --> 1

Some trees:

Char. 368: 0 --> 2

Heterodontosaurus_tucki :

All trees:

Char. 12: 0 --> 1

Char. 20: 0 --> 1

Char. 62: 0 --> 2

Char. 79: 0 --> 1

Char. 90: 0 --> 1

Char. 102: 0 --> 1

Char. 158: 0 --> 1

Char. 160: 0 --> 1

Char. 191: 0 --> 2

Char. 244: 0 --> 1

Char. 250: 0 --> 1

Char. 267: 2 --> 3

Char. 323: 0 --> 1

Char. 333: 1 --> 0

Char. 360: 0 --> 1

Lesothosaurus_diagnosticus :

All trees:

Char. 83: 0 --> 1

Char. 161: 0 --> 1

Char. 162: 0 --> 1

Char. 237: 1 --> 0

Char. 269: 0 --> 1

Char. 324: 1 --> 2

Herrerasaurus_ischigualastensis :

All trees:

Char. 173: 1 --> 0

Char. 181: 0 --> 1

Char. 194: 1 --> 0

Char. 210: 0 --> 1

Char. 241: 1 --> 0

Char. 287: 0 --> 1

Char. 298: 0 --> 1

Eoraptor_lunensis :

All trees:

Char. 37: 0 --> 1

Char. 152: 1 --> 0

Char. 167: 1 --> 0

Char. 257: 1 --> 0

Char. 302: 2 --> 1

Char. 305: 1 --> 0

Char. 326: 1 --> 0

Saturnalia_tupiniquim :

All trees:

Char. 232: 1 --> 0

Char. 269: 0 --> 1

Char. 271: 2 --> 1

Char. 323: 0 --> 1

Plateosaurus_engelhardti :

All trees:

Char. 25: 0 --> 1

Char. 146: 0 --> 1

Char. 161: 0 --> 1

Char. 194: 1 --> 0

Char. 203: 0 --> 1

Char. 211: 1 --> 0

Char. 379: 1 --> 0

Efraasia_minor :

All trees:

Char. 174: 0 --> 1

Char. 281: 1 --> 0

Coelophysis_bauri :

All trees:

Char. 0: 0 --> 1

Char. 2: 0 --> 1

Char. 4: 0 --> 1

Char. 22: 0 --> 1

Char. 72: 0 --> 1

Char. 102: 0 --> 1

Char. 174: 0 --> 1

Char. 176: 0 --> 1

Char. 177: 0 --> 1

Char. 198: 0 --> 1

Char. 203: 0 --> 1

Char. 204: 0 --> 1

Char. 263: 0 --> 1

Char. 266: 0 --> 1

Char. 269: 1 --> 2

Char. 273: 0 --> 1

Char. 275: 0 --> 1

Char. 292: 0 --> 1

Char. 311: 1 --> 0

Char. 325: 0 --> 1

Char. 341: 1 --> 0

Char. 360: 0 --> 1

Char. 375: 0 --> 1

Yonghesuchus_sangbiensis :

All trees:

Char. 11: 1 --> 2

Char. 13: 0 --> 1

Char. 79: 1 --> 0

Char. 398: 0 --> 1

Garjainia_prima :

All trees:

Char. 28: 0 --> 1

Char. 29: 0 --> 1

Char. 67: 1 --> 0

Char. 119: 1 --> 0

Sarmatosuchus_otschevi :

Some trees:

Char. 183: 0 --> 1

Shansisuchus_shansisuchus :

All trees:

Char. 3: 0 --> 3

Char. 48: 0 --> 1

Char. 274: 1 --> 0

Char. 314: 0 --> 1

Char. 330: 0 --> 1

Char. 333: 0 --> 1

Char. 334: 0 --> 1

Char. 345: 0 --> 1

Char. 352: 0 --> 1

Some trees:

Char. 5: 2 --> 3

Doswellia_kaltenbachi :

All trees:

Char. 202: 0 --> 1

Char. 205: 0 --> 1

Char. 308: 0 --> 1

Char. 314: 0 --> 1

Char. 395: 0 --> 2

Jaxtasuchus_salomoni :

All trees:

Char. 174: 0 --> 1

Char. 176: 0 --> 1

Char. 300: 1 --> 2

Archeopelta_arborensis :

All trees:

Char. 120: 1 --> 0

Char. 294: 0 --> 1

Char. 296: 0 --> 1

Char. 297: 1 --> 0

Char. 298: 0 --> 1

Char. 319: 0 --> 1

Proterochampsa_barrionuevoi :

All trees:

Char. 29: 0 --> 1

Char. 32: 0 --> 1

Char. 55: 0 --> 1

Char. 61: 0 --> 1

Char. 63: 0 --> 1

Char. 82: 0 --> 1

Char. 114: 1 --> 0

Char. 166: 0 --> 1

Char. 183: 0 --> 1

Char. 192: 0 --> 1

Char. 193: 0 --> 1

Some trees:

Char. 399: 0 --> 1

Protorosaurus_speneri :

All trees:

Char. 31: 1 --> 0

Char. 41: 0 --> 1

Char. 61: 0 --> 1

Char. 62: 0 --> 1

Char. 72: 0 --> 1

Char. 150: 0 --> 1

Char. 163: 1 --> 0

Char. 165: 0 --> 2

Char. 184: 0 --> 1

Char. 188: 0 --> 1

Char. 191: 0 --> 1

Char. 193: 0 --> 1

Char. 246: 0 --> 1

Char. 259: 0 --> 1

Char. 300: 1 --> 2

Char. 315: 0 --> 1

Trilophosaurus_buettneri :

All trees:

Char. 46: 0 --> 1

Char. 62: 0 --> 2

Char. 67: 0 --> 1

Char. 69: 0 --> 1

Char. 70: 0 --> 1

Char. 88: 0 --> 1

Char. 109: 0 --> 1

Char. 127: 0 --> 1

Char. 147: 0 --> 1

Char. 167: 0 --> 1

Char. 169: 0 --> 1

Char. 187: 0 --> 1

Char. 197: 0 --> 1

Char. 241: 0 --> 1

Char. 245: 01 --> 2

Char. 321: 0 --> 1

Char. 334: 0 --> 1

Youngina_capensis :

All trees:

No autapomorphies:

Koilamasuchus_gonzalezdiazi :

All trees:

Char. 197: 1 --> 0

Char. 235: 0 --> 1

Turfanosuchus_shageduensis :

All trees:

No autapomorphies:

Halazhaisuchus_qiaoensis :

Some trees:

Char. 179: 0 --> 1

Osmolskina_czatkowicensis :

Some trees:

Char. 323: 0 --> 1

Char. 334: 0 --> 1

Dorosuchus_neoetus :

All trees:

Char. 96: 1 --> 0

Char. 267: 1 --> 0

Node 65 :

All trees:

Char. 91: 0 --> 1

Char. 166: 1 --> 0

Char. 215: 1 --> 0

Char. 234: 1 --> 0

Char. 236: 0 --> 1

Char. 257: 1 --> 0

Char. 300: 1 --> 0

Char. 379: 1 --> 0

Node 66 :

All trees:

Char. 0: 1 --> 0

Char. 3: 2 --> 0

Char. 4: 1 --> 0

Char. 18: 1 --> 0

Char. 22: 1 --> 0

Char. 235: 1 --> 0

Char. 249: 0 --> 1

Char. 312: 1 --> 0

Char. 366: 1 --> 0

Node 67 :

All trees:

No synapomorphies

Node 68 :

All trees:

Char. 31: 1 --> 0

Char. 66: 1 --> 0

Char. 98: 1 --> 0

Char. 163: 1 --> 0

Char. 164: 0 --> 1

Char. 288: 1 --> 0

Some trees:

Char. 191: 0 --> 1

Node 69 :

All trees:

Char. 67: 0 --> 1

Char. 94: 0 --> 1

Char. 128: 0 --> 1

Char. 130: 0 --> 1

Char. 152: 0 --> 1

Char. 159: 0 --> 1

Char. 274: 0 --> 1

Some trees:

Char. 7: 0 --> 12

Char. 114: 0 --> 1

Node 70 :

All trees:

Char. 13: 0 --> 2

Char. 80: 0 --> 1

Char. 308: 0 --> 1

Node 71 :

All trees:

Char. 120: 1 --> 0

Char. 167: 0 --> 1

Char. 194: 1 --> 0

Char. 228: 0 --> 1

Char. 235: 0 --> 1

Some trees:

Char. 183: 0 --> 1

Node 72 :

All trees:

Char. 95: 0 --> 1

Char. 96: 0 --> 1

Char. 165: 0 --> 1

Char. 217: 0 --> 1

Char. 220: 0 --> 1

Some trees:

Char. 28: 1 --> 0

Char. 119: 0 --> 1

Char. 137: 0 --> 1

Char. 156: 0 --> 1

Char. 191: 1 --> 0

Char. 197: 0 --> 1

Char. 201: 0 --> 1

Char. 215: 0 --> 1

Char. 221: 1 --> 0

Char. 265: 0 --> 1

Char. 337: 0 --> 1

Char. 359: 0 --> 1

Char. 371: 0 --> 1

Char. 383: 0 --> 1

Node 73 :

All trees:

Char. 32: 0 --> 1

Char. 130: 1 --> 0

Node 74 :

All trees:

Char. 98: 0 --> 1

Char. 171: 0 --> 1

Char. 312: 0 --> 1

Char. 390: 0 --> 1

Some trees:

Char. 7: 1 --> 0

Node 75 :

All trees:

Char. 0: 0 --> 1

Char. 38: 0 --> 1

Char. 51: 0 --> 1

Char. 59: 0 --> 1

Char. 74: 0 --> 1

Node 76 :

All trees:

Char. 10: 0 --> 1

Char. 31: 0 --> 1

Char. 39: 0 --> 1

Char. 43: 0 --> 1

Char. 44: 0 --> 1

Char. 52: 0 --> 1

Char. 79: 0 --> 1

Char. 150: 0 --> 1

Char. 151: 0 --> 1

Char. 170: 0 --> 1

Char. 391: 1 --> 0

Node 77 :

All trees:

Char. 53: 0 --> 1

Char. 333: 0 --> 1

Char. 355: 0 --> 1

Char. 367: 0 --> 1

Node 78 :

All trees:

Char. 5: 2 --> 1

Char. 28: 0 --> 1

Char. 114: 1 --> 0

Char. 138: 1 --> 0

Char. 392: 0 --> 1

Some trees:

Char. 399: 0 --> 1

Node 79 :

All trees:

Char. 149: 0 --> 1

Char. 182: 0 --> 1

Char. 191: 0 --> 1

Char. 335: 1 --> 0

Char. 350: 0 --> 1

Char. 366: 0 --> 1

Node 80 :

All trees:

Char. 5: 2 --> 3

Char. 9: 0 --> 1

Char. 15: 0 --> 1

Char. 18: 0 --> 1

Char. 32: 0 --> 1

Char. 50: 0 --> 1

Char. 81: 0 --> 1

Char. 131: 0 --> 1

Char. 142: 0 --> 1

Char. 153: 0 --> 1

Char. 158: 0 --> 1

Char. 226: 0 --> 1

Char. 233: 1 --> 0

Char. 329: 0 --> 1

Char. 396: 0 --> 1

Some trees:

Char. 331: 1 --> 0

Char. 368: 0 --> 1

Node 81 :

All trees:

Char. 100: 0 --> 1

Node 82 :

All trees:

Char. 167: 0 --> 1

Node 83 :

All trees:

Char. 104: 0 --> 1

Char. 131: 1 --> 2

Char. 330: 0 --> 1

Char. 376: 0 --> 1

Node 84 :

All trees:

Char. 70: 0 --> 1

Char. 77: 0 --> 1

Char. 102: 0 --> 1

Node 85 :

All trees:

Char. 3: 0 --> 3

Char. 11: 0 --> 1

Char. 30: 0 --> 2

Char. 37: 0 --> 1

Char. 46: 0 --> 1

Char. 365: 0 --> 1

Char. 393: 0 --> 1

Some trees:

Char. 71: 0 --> 1

Node 86 :

All trees:

Char. 190: 0 --> 1

Char. 343: 0 --> 1

Char. 392: 0 --> 1

Char. 402: 0 --> 1

Char. 403: 1 --> 0

Some trees:

Char. 284: 0 --> 2

Node 87 :

All trees:

Char. 238: 0 --> 1

Char. 327: 0 --> 1

Some trees:

Char. 115: 0 --> 1

Char. 321: 0 --> 1

Node 88 :

All trees:

Char. 29: 0 --> 1

Char. 116: 0 --> 1

Char. 118: 0 --> 1

Char. 129: 1 --> 2

Char. 225: 0 --> 1

Char. 237: 0 --> 1

Char. 243: 0 --> 1

Char. 296: 0 --> 1

Char. 356: 0 --> 1

Char. 367: 1 --> 2

Node 89 :

All trees:

Char. 5: 2 --> 0

Char. 7: 0 --> 2

Char. 11: 0 --> 1

Char. 14: 0 --> 1

Char. 33: 0 --> 1

Char. 86: 0 --> 1

Char. 134: 0 --> 2

Char. 153: 0 --> 1

Char. 181: 0 --> 1

Char. 194: 1 --> 0

Char. 277: 0 --> 1

Char. 303: 0 --> 1

Char. 358: 1 --> 2

Some trees:

Char. 65: 0 --> 1

Char. 183: 0 --> 1

Char. 267: 2 --> 1

Char. 329: 0 --> 1

Node 90 :

All trees:

Char. 78: 0 --> 1

Char. 159: 1 --> 0

Char. 219: 0 --> 1

Node 91 :

All trees:

Char. 6: 0 --> 1

Char. 11: 0 --> 3

Char. 22: 0 --> 1

Char. 23: 0 --> 1

Char. 30: 0 --> 1

Char. 49: 0 --> 1

Char. 64: 0 --> 1

Char. 84: 0 --> 1

Char. 135: 0 --> 1

Char. 146: 0 --> 2

Char. 147: 0 --> 1

Char. 148: 0 --> 1

Char. 151: 0 --> 1

Char. 157: 0 --> 1

Char. 162: 0 --> 1

Some trees:

Char. 65: 0 --> 1

Node 92 :

All trees:

Char. 17: 0 --> 1

Char. 58: 0 --> 1

Char. 70: 0 --> 1

Char. 76: 0 --> 2

Char. 163: 0 --> 1

Char. 240: 0 --> 1

Char. 394: 0 --> 1

Char. 400: 0 --> 1

Char. 401: 0 --> 1

Char. 404: 0 --> 2

Some trees:

Char. 71: 0 --> 1

Char. 118: 1 --> 2

Char. 233: 1 --> 0

Char. 241: 0 --> 1

Char. 362: 0 --> 1

Node 93 :

All trees:

Char. 187: 0 --> 1

Char. 289: 0 --> 1

Char. 294: 0 --> 1

Some trees:

Char. 124: 0 --> 1

Char. 208: 0 --> 1

Char. 291: 0 --> 1

Char. 292: 0 --> 1

Char. 308: 0 --> 1

Char. 362: 0 --> 1

Node 94 :

All trees:

Char. 0: 0 --> 1

Char. 4: 0 --> 1

Char. 22: 0 --> 1

Char. 23: 0 --> 1

Char. 191: 1 --> 0

Char. 200: 0 --> 1

Char. 204: 0 --> 1

Char. 271: 0 --> 2

Char. 285: 0 --> 1

Char. 335: 0 --> 1

Some trees:

Char. 175: 0 --> 1

Char. 189: 1 --> 0

Char. 297: 1 --> 0

Char. 313: 0 --> 1

Char. 317: 0 --> 1

Node 95 :

All trees:

Char. 98: 1 --> 0

Char. 186: 0 --> 1

Node 96 :

All trees:

Char. 183: 0 --> 1

Char. 277: 0 --> 1

Char. 330: 0 --> 1

Char. 361: 1 --> 2

Some trees:

Char. 1: 0 --> 1

Char. 5: 2 --> 1

Char. 25: 0 --> 1

Char. 56: 0 --> 1

Char. 57: 0 --> 1

Char. 134: 0 --> 1

Char. 284: 2 --> 1

Char. 331: 1 --> 0

Char. 386: 0 --> 1

Node 97 :

All trees:

Char. 45: 0 --> 1

Char. 106: 0 --> 1

Char. 139: 0 --> 1

Char. 348: 0 --> 1

Node 98 :

All trees:

Char. 13: 0 --> 1

Char. 34: 0 --> 1

Char. 37: 0 --> 1

Char. 46: 0 --> 1

Char. 54: 0 --> 1

Char. 62: 0 --> 1

Char. 89: 0 --> 1

Char. 123: 0 --> 1

Char. 133: 0 --> 1

Char. 136: 0 --> 1

Char. 313: 0 --> 1

Char. 393: 0 --> 1

Char. 399: 0 --> 1

Char. 402: 1 --> 0

Char. 403: 0 --> 1

Node 99 :

All trees:

Char. 306: 0 --> 1

Char. 308: 1 --> 0

Char. 334: 0 --> 1

Char. 365: 0 --> 1

Node 100 :

All trees:

Char. 35: 0 --> 1

Char. 57: 1 --> 2

Char. 79: 1 --> 2

Node 101 :

All trees:

Char. 146: 0 --> 2

Char. 191: 1 --> 0

Char. 331: 0 --> 1

Node 102 :

All trees:

Char. 3: 0 --> 1

Char. 12: 0 --> 1

Char. 41: 0 --> 1

Char. 47: 0 --> 1

Char. 57: 1 --> 0

Char. 59: 0 --> 1

Char. 83: 0 --> 1

Char. 134: 1 --> 0

Char. 194: 0 --> 1

Char. 223: 0 --> 1

Char. 372: 0 --> 1

Node 103 :

All trees:

Char. 0: 0 --> 1

Char. 22: 0 --> 1

Char. 23: 0 --> 1

Char. 126: 0 --> 1

Char. 158: 0 --> 1

Char. 174: 0 --> 1

Char. 209: 0 --> 1

Char. 223: 0 --> 1

Char. 242: 0 --> 1

Char. 261: 0 --> 1

Char. 266: 0 --> 1

Char. 377: 0 --> 1

Char. 386: 0 --> 1

Node 104 :

All trees:

Char. 182: 1 --> 0

Char. 191: 1 --> 0

Char. 229: 0 --> 1

Char. 257: 0 --> 1

Char. 258: 0 --> 1

Char. 295: 0 --> 1

Char. 338: 0 --> 1

Char. 339: 0 --> 1

Char. 347: 0 --> 1

Char. 351: 0 --> 1

Char. 372: 0 --> 1

Char. 390: 1 --> 0

Char. 404: 0 --> 1

Some trees:

Char. 184: 0 --> 1

Node 105 :

All trees:

Char. 301: 0 --> 1

Char. 320: 0 --> 1

Char. 373: 0 --> 1

Char. 379: 0 --> 1

Char. 387: 0 --> 1

Char. 388: 0 --> 2

Node 106 :

All trees:

Char. 192: 0 --> 1

Char. 280: 0 --> 1

Char. 283: 0 --> 1

Char. 284: 0 --> 1

Char. 293: 0 --> 1

Char. 294: 0 --> 1

Char. 303: 0 --> 1

Char. 305: 0 --> 1

Char. 328: 0 --> 1

Char. 346: 0 --> 1

Node 107 :

All trees:

Char. 147: 0 --> 1

Char. 157: 0 --> 1

Char. 162: 0 --> 1

Char. 163: 0 --> 1

Char. 205: 0 --> 1

Char. 304: 0 --> 1

Char. 318: 0 --> 1

Node 108 :

All trees:

Char. 110: 0 --> 1

Char. 121: 0 --> 1

Char. 174: 0 --> 1

Char. 308: 0 --> 1

Char. 379: 1 --> 0

Node 109 :

All trees:

Char. 101: 0 --> 1

Char. 172: 0 --> 1

Char. 264: 0 --> 2

Char. 277: 0 --> 1

Char. 289: 0 --> 1

Char. 302: 0 --> 1

Char. 324: 0 --> 1

Char. 332: 0 --> 1

Char. 374: 0 --> 1

Char. 381: 0 --> 1

Char. 385: 0 --> 1

Node 110 :

All trees:

Char. 15: 0 --> 1

Char. 21: 0 --> 1

Char. 46: 0 --> 1

Char. 66: 0 --> 1

Char. 139: 0 --> 1

Char. 143: 0 --> 1

Char. 152: 1 --> 0

Char. 154: 0 --> 1

Char. 155: 0 --> 2

Char. 163: 0 --> 1

Char. 193: 0 --> 1

Char. 202: 0 --> 1

Char. 203: 0 --> 1

Char. 204: 0 --> 1

Char. 266: 0 --> 1

Char. 278: 0 --> 1

Char. 279: 0 --> 1

Char. 283: 1 --> 0

Char. 287: 0 --> 1

Char. 303: 1 --> 2

Char. 305: 1 --> 0

Node 111 :

All trees:

Char. 112: 0 --> 1

Char. 136: 0 --> 1

Char. 178: 0 --> 1

Char. 211: 0 --> 1

Char. 228: 0 --> 1

Char. 241: 0 --> 1

Char. 293: 1 --> 2

Char. 310: 0 --> 1

Char. 311: 0 --> 1

Char. 320: 1 --> 2

Char. 326: 0 --> 1

Char. 352: 0 --> 1

Some trees:

Char. 368: 0 --> 2

Node 112 :

All trees:

Char. 16: 1 --> 0

Char. 79: 0 --> 1

Char. 209: 0 --> 1

Char. 230: 0 --> 1

Char. 244: 0 --> 1

Char. 250: 0 --> 1

Char. 258: 2 --> 3

Char. 260: 0 --> 1

Char. 322: 0 --> 1

Some trees:

Char. 282: 0 --> 1

Node 113 :

All trees:

Char. 13: 0 --> 1

Char. 40: 0 --> 1

Char. 179: 0 --> 1

Char. 206: 0 --> 1

Char. 245: 0 --> 1

Char. 253: 0 --> 1

Char. 254: 0 --> 1

Char. 281: 0 --> 1

Char. 291: 0 --> 2

Char. 302: 1 --> 2

Char. 316: 0 --> 1

Char. 341: 0 --> 1

Char. 342: 0 --> 1

Node 114 :

All trees:

Char. 12: 0 --> 1

Char. 24: 0 --> 1

Char. 73: 0 --> 1

Char. 269: 0 --> 1

Char. 270: 0 --> 1

Char. 276: 0 --> 1

Char. 291: 2 --> 1

Node 115 :

All trees:

Char. 127: 0 --> 1

Char. 162: 0 --> 1

Char. 235: 0 --> 1

Char. 292: 0 --> 1

Char. 308: 0 --> 1

Char. 325: 0 --> 1

Char. 336: 1 --> 0

Node 116 :

All trees:

Char. 305: 1 --> 0

Char. 373: 1 --> 0

Node 117 :

All trees:

Char. 95: 1 --> 0

Char. 191: 0 --> 1

Char. 192: 0 --> 1

Char. 398: 0 --> 1

Node 118 :

All trees:

Char. 193: 0 --> 1

Char. 275: 0 --> 1

Char. 396: 0 --> 2

Char. 397: 0 --> 1

Char. 400: 0 --> 1

Node 119 :

All trees:

Char. 274: 1 --> 0

Node 120 :

All trees:

Char. 219: 0 --> 1
